# Assessing the implementation fidelity, feasibility, and sustainability of community-based house improvement for malaria control in southern Malawi: a mixed-methods study

**DOI:** 10.1186/s12889-024-18401-4

**Published:** 2024-04-02

**Authors:** Tinashe A. Tizifa, Alinune N. Kabaghe, Robert S. McCann, Steven Gowelo, Tumaini Malenga, Richard M. Nkhata, Yankho Chapeta, William Nkhono, Asante Kadama, Willem Takken, Kamija S. Phiri, Michele van Vugt, Henk van den Berg, Lucinda Manda-Taylor

**Affiliations:** 1 Center for Tropical Medicine and Travel Medicine, Department of Infectious Diseases, Division of Internal Medicine, University of Amsterdam, Amsterdam University Medical Center, Location Academic Medical Center, Amsterdam, The Netherlands; 2 School of Global and Public Health, Kamuzu University of Health Sciences, Blantyre, Malawi; 3 Laboratory of Entomology, Wageningen University & Research, Wageningen, The Netherlands; 4 Centre for Vaccine Development and Global Health, University of Maryland School of Medicine, Baltimore, USA; 5 National TB and Leprosy Elimination Programme, Ministry of Health, Lilongwe, Malawi; 6 Biological Sciences Department, Mzuzu University, Mzuzu, Malawi

**Keywords:** Malaria, House Improvement, Implementation, Fidelity, Feasibility, Sustainability, Malawi

## Abstract

**Background:**

Despite significant success in the fight against malaria over the past two decades, malaria control programmes rely on only two insecticidal methods: indoor residual spraying and insecticidal-treated nets. House improvement (HI) can complement these interventions by reducing human-mosquito contact, thereby reinforcing the gains in disease reduction. This study assessed the implementation fidelity, which is the assessment of how closely an intervention aligns with its intended design, feasibility, and sustainability of community-led HI in southern Malawi.

**Methods:**

The study, conducted in 22 villages (2730 households), employed a mixed-methods approach. Implementation fidelity was assessed using a modified framework, with longitudinal surveys collecting data on HI coverage indicators. Quantitative analysis, employing descriptive statistics, evaluated the adherence to HI implementation. Qualitative data came from in-depth interviews, key informant interviews, and focus groups involving project beneficiaries and implementers. Qualitative data were analysed using content analysis guided by the implementation fidelity model to explore facilitators, challenges, and factors affecting intervention feasibility.

**Results:**

The results show that HI was implemented as planned. There was good adherence to the intended community-led HI design; however, the adherence could have been higher but gradually declined over time. In terms of intervention implementation, 74% of houses had attempted to have eaves closed in 2016-17 and 2017-18, compared to 70% in 2018–19. In 2016–17, 42% of houses had all four sides of the eaves closed, compared to 33% in 2018–19. Approximately 72% of houses were screened with gauze wire in 2016-17, compared to 57% in 2018-19. High costs, supply shortages, labour demands, volunteers’ poor living conditions and adverse weather were reported to hinder the ideal HI implementation. Overall, the community described community-led HI as feasible and could be sustained by addressing these socioeconomic and contextual challenges.

**Conclusion:**

Our study found that although HI was initially implemented as planned, its fidelity declined over time. Using trained volunteers facilitated the fidelity and feasibility of implementing the intervention. A combination of rigorous community education, consistent training, information, education and communication, and intervention modifications may be necessary to address the challenges and enhance the intervention’s fidelity, feasibility, and sustainability.

**Supplementary Information:**

The online version contains supplementary material available at 10.1186/s12889-024-18401-4.

## Introduction

Mass distribution of insecticide-treated nets (ITNs) and indoor residual spraying (IRS) have been prioritised as the primary methods for large-scale deployment of malaria vector control worldwide [1]. Modelling studies suggest that these approaches have had a major impact on malaria cases in sub-Saharan Africa [2]. However, it is acknowledged that further reduction and progress toward malaria elimination will necessitate innovative and complementary approaches [3]. Even with the best ITN coverage, residual malaria parasite transmission may continue because malaria vectors can feed before sleeping times, indoors and outdoors, when ITNs are not used [4]. Furthermore, some vector species may enter houses to feed and then exit to rest outdoors, avoiding fatal contact with insecticide-treated surfaces [5]. Additional challenges to the long-term sustainability of ITNs and IRS include the development of insecticide resistance in vector populations to pyrethroids and other classes of insecticides [6].

The use of additional supplemental interventions such as house improvement (HI) has been recommended as a potential strategy to further contribute to malaria control, especially in low and middle-income countries (LMIC). People with low income may live in houses that allow easy mosquito entry [1, 7]. HI includes a range of related interventions such as the screening of doors and windows, closing or screening eaves, patching cracks in walls, and installing ceilings, to reduce contact between malaria vectors and humans by preventing mosquito entry into houses or making houses less amenable for resting mosquitoes and thus may reduce malaria parasite transmission [8]. HI contributed to eliminating malaria from parts of North America and Europe before developing insecticides such as dichlorodiphenyltrichloroethane (DDT) in the 1950s, and HI was neglected after that [9].

Building on this historical perspective, in the current landscape of malaria control, several studies have shown that house improvements can reduce mosquito bites and lower the risk of house occupants contracting malaria [10–12]. HI trials in Africa have yielded compelling results, underscoring the pivotal role of housing conditions. For instance, studies have consistently shown that simple enhancements, such as screening windows and doors and closing eaves, can significantly reduce vector density indoors, thereby mitigating the risk of malaria incidence and related complications [13–18]. Noteworthy evidence from trials in various African countries affirms the sustainability and efficacy of house screening in preventing mosquito entry [16–20]. Additionally, a study in southern Malawi specifically focusing on HI, involving the closure of eaves using locally available materials, revealed a notable decrease in malaria vectors within houses, with variations based on the degree of eave closure [21].

Unlike ITNs and IRS, which depend on insecticides for impregnation and spraying, respectively, and are often expensive in many settings, HI presents a distinctive economic advantage. While ITNs and IRS involve recurrent costs for insecticides and application, HI typically incurs upfront costs primarily related to the procurement of construction materials [22, 23]. This unique characteristic distinguishes HI from other malaria control interventions, offering a potential cost-saving aspect over the long term once the initial investment is made.

The success of vector control programmes depends on the entomological and epidemiological effects of the proposed interventions, the access to the interventions by the target population, and the uptake and appropriate use. To determine whether interventions will be adopted and valuable, it is necessary first to understand the social, cultural and contextual factors that may influence the implementation outcomes of an intervention [24, 25]. The perceptions and acceptability of community-based HI have been demonstrated in previous studies [26, 27]. However, there is a lack of evidence and experience regarding the routine implementation of community-based HI. Analysing the feasibility and its fidelity is crucial to understanding the specific reasons contributing to the success or failure of the intervention [28–31]. Such information is essential in offering feedback aimed at enhancing the implementation process. Fidelity is the degree to which an intervention was implemented as intended, planned, and designed [25, 28, 32, 33]. Several studies in the field of medical research have shown that programmes with high fidelity are associated with better outcomes than programmes with lower fidelity [34–40]. Feasibility is the success of implementing an intervention within a specified setting and the extent to which a new treatment or intervention can be successfully carried out [25]. In practice, human, financial, and material resources are required to implement an intervention [41]. The sustainability of implementation is a critical aspect as far as any programme’s lifecycle is concerned. It is defined as the extent to which a newly implemented programme or intervention is maintained or institutionalised within a service setting’s ongoing, stable operations [25].

To address this gap, this study uniquely assesses the implementation fidelity, feasibility, and sustainability of community-led HI in the Majete Wildlife Reserve (MWR) communities in Chikwawa district, southern Malawi. What sets this investigation apart is its focus on evaluating the performance of community-led HI within the context of routine uptake. This distinctive approach allows for a comprehensive understanding of how the intervention aligns with everyday practices, providing valuable insights into the practicality and potential longevity of the intervention in the context of malaria control.

## Methods

### Study design

This study used a mixed-methods approach with the following components: (i) a qualitative study with focus group discussions (FGDs), in-depth interviews (IDIs), and key informant interviews (KIIs) to assess the feasibility, fidelity, and sustainability of the community-led implementation of HI. The methodology of the qualitative component was adapted from a previous study, as it involved the same study setting and population [27]; (ii) Assessment of the quality of house structures, using HI coverage indicators, acquired through longitudinal surveys (2016–2019) to determine the standard and quality at which HI was carried out at household level in the villages [42]. Differences in house modification between HI and non-HI villages were used to estimate changes in house structure attributable to the intervention.

### Study setting

The study was conducted in the communities surrounding the MWR perimeter located in Chikwawa district. The MWR perimeter is host to about 90,000 people. The study area has been described in detail elsewhere [43]. Agriculture is the key livelihood activity in this district [44]. Domestic animal rearing cattle, goats, sheep, pigs, and chickens is a secondary and supplemental income-generating activity [44].

The primary public health problems in the area are malaria, diarrhoea, acute respiratory infections (including pneumonia), skin infections, common injuries and wounds, and sexually transmitted diseases [44]. Historically, this area has had high malaria transmission rates [45]. Malaria transmission in this area is predominantly by *Anopheles arabiensis* and *Anopheles funestus*, with a small proportion of *Anopheles gambiae s.s* [46–48]. Malaria control in the district follows the National Malaria Control Programme (NMCP) strategy and is implemented through the Chikwawa District Health Office. Between 2015 and 2018, the malaria control strategy included providing ITNs to pregnant women and children under 5 years old, conducting mass distribution campaigns of ITNs, offering intermittent preventative therapy for pregnant women, and diagnosing and treating malaria cases with artemisinin-based combination therapy [48]. During this period, the last mass distribution of ITNs in the district took place in April 2016 and included PermaNet® 2.0 (Vestergaard Frandsen, Lausanne, Switzerland), Olyset® Net (Sumitomo Chemical Company, Tokyo, Japan), and Royal Sentry® (Disease Control Technologies, USA) [48]. The NMCP implemented IRS in Chikwawa District in 2010 and 2012, using alphacypermethrin. However, IRS was not conducted in the district during the study period [48].

### The HI intervention

HI was implemented in 22 villages as part of the Majete Malaria Project (MMP), a five-year community-led malaria control project implemented to investigate the effect of community-driven larval source management (LSM) and HI on malaria transmission when added to the standard malaria control strategies [42, 49, 50]. From August 2014 to February 2015, we carried out an enumeration exercise [42]. During this period, we gathered information on the name, gender, date of birth, and relationship to the head of the household for every household member. In this context, a household was defined as “a social group composed of individuals sharing meals from the same pot [42].” The trial was conducted from May 2016 through May 2018 and continued until April 2019 as a rolled-out intervention in the rest of the villages within the MWR perimeter. The trial interventions, HI and LSM, were implemented as supplementary to the recommended interventions by the NMCP. The research setting was part of an intensive community education and engagement programme to enhance community participation in malaria control [42, 48, 49].

In this trial, HI was referred to as the material modification of houses designed to prevent the entry of malaria vectors. The intervention design and rationale were iteratively developed through a review of the literature, consultation with the communities, and training of implementers to create activities that met local requirements and had the potential for long-term sustainability [42, 49]. In summary, the intervention involved specific activities aimed at mobilising the community to implement effective house improvements. The agreed-upon modifications consisted of the following: closing all eaves (i.e., where a wall meets the overhang of the roof) using local materials similar to those used in house construction (i.e., bricks and extra mud for most houses); closing all holes in the wall not used for ventilation using the same materials used for closing eaves; covering windows and other openings used for ventilation with aluminium screens that allow airflow; and modifying doors to fully cover doorways when closed (Additional file 1). All these activities were conducted by the local community. The MMP conducted capacity building for local volunteers and provided locally procured mosquito wire mesh and basic hand tools (e.g., scissors and a tape measure). All other materials were provided by the local community. The implementation of HI in the 22 selected villages was a continuous process initiated during the pre-trial period from April 2015 to April 2016. By September 2016, field visits to the study villages indicated successful HI implementation in all villages, with each village having at least one demonstration house featuring properly sealed eaves and screened windows [49]. The intervention and its implementation process are further described in detail in the main articles [42, 49].

**Fig. 1:**
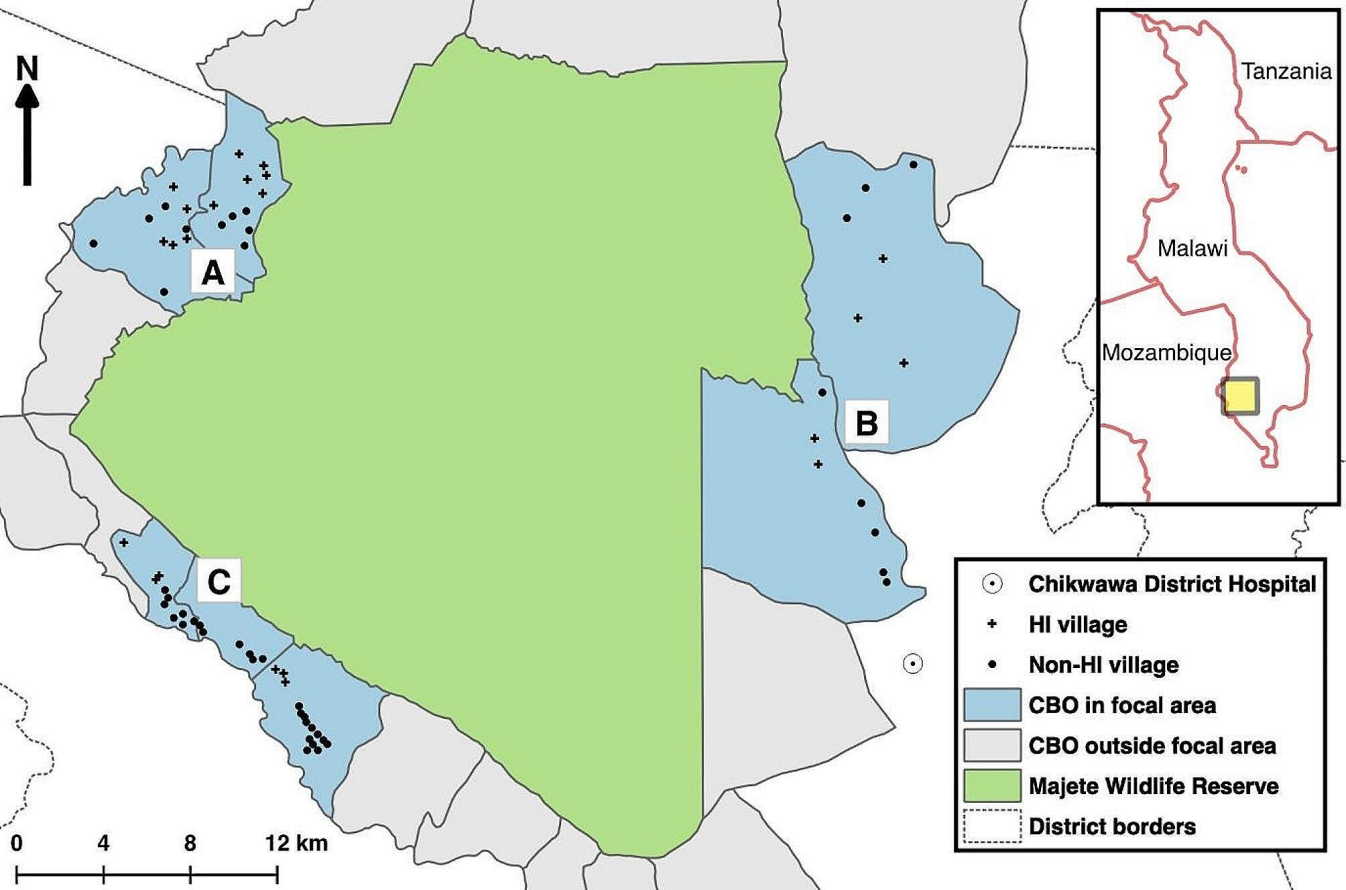
Map of Majete Wildlife Reserve and Majete Perimeter, comprising 19 groups of villages known as community-based organisations (CBOs). The map shows three focal areas labelled A, B, and C, each with its respective villages (HI and non-HI villages) participating in the community-led cluster-randomised controlled HI-LSM trial. Adapted with permission from [42, 43, 49]

All MMP trial interventions were conducted at the village level, with the trial consisting of four arms. Villages were randomly assigned to one of these four arms: (a) a control arm, (b) HI, (c) LSM, and (d) HI + LSM [42, 48]. All arms used interventions recommended by the NMCP and community engagement [42, 48]. A two-stage randomisation process occurred within each focal area during a community event in June 2015. The randomisation process of the trial, which includes the allocation of villages across the study arms in the focal areas, is further described in detail in these articles [42, 48, 49]. The qualitative component of this study included all 22 villages participating in the HI intervention, i.e., the HI and HI + LSM arms. Non-HI villages consisted of villages from both the control arm and the LSM arm. There were 31 non-HI villages, comprising 20 LSM villages and 11 control villages. To minimise the risk of contamination between different treatment arms, twelve villages were excluded from the treatment arm allocation. As a result, a total of 53 villages were assigned to the four trial arms in June 2015 [48]. Thus, in total, the trial catchment area contained 65 villages. The quantitative component of the study included both HI and non-HI villages as a basis for comparison to determine if the introduction of HI was significant to the house modifications. The study villages were divided into three sub-regions, referred to as focal areas (A, B, and C), spaced evenly around the perimeter of the MWR, covering a total population of about 25,000 people in 65 villages and approximately 6,600 households (Fig. 1).

### Study population

In the qualitative component of this study, all participants resided in 22 HI villages located across the three focal areas. Table 1 below presents the five different groups of participants identified in this study along with their respective functions.

**Table 1:** Participant groups for the qualitative component and their respective functions

Participant Group	Description
Health animators (HAs)	Volunteers specially trained in healthcare, holding an honorary position, dedicated to improving community health outcomes. Selected based on literacy skills, leadership potential, and motivation levels. Received education and training in community-led malaria control from MMP and The Hunger Project.
HI committee members	Volunteers from villages, 8 to 10 individuals per committee, were selected during community meetings. Responsible for conducting HI activities, storing equipment, distributing materials, and coordinating community participation in HI implementation.
Members from the broader community	Responsible for implementing HI in their houses, including full eave closure, sealing open spaces, and placement of gauze wire on windows.
Health Surveillance Assistants (HSAs)	Paid healthcare providers deployed by the government to urban, rural, and hard-to-reach areas. Tasked with promoting the implementation of HI as an intervention for malaria control.
Village Chiefs	Primary gatekeepers of communities, are selected based on their role in facilitating development. Tasked with overseeing HI implementation in their respective villages.

The roles of health animators (HA model) are further described elsewhere [27, 50–52]. Other potential study participants such as HI committee drop-outs (e.g., individuals who once were part of the HI committees but relinquished their positions due to other circumstances) and non-participants (individuals who refused to implement HI) of the trial were considered. However logistical challenges, including relocations and refusals, prevented their inclusion in the interviews.

In the quantitative component of this study, households were sampled from both HI and non-HI villages located across the three focal areas. The households in these villages were assessed during this phase through the administration of a questionnaire checklist to the household owners. In the context of this study, the intervention’s implementation was community-led, involving trained volunteers and other community stakeholders.

### Determination of implementation outcomes

Through qualitative surveys, the feasibility of implementing HI was determined based on the community’s perceptions of the workload involved, the available resources needed to implement HI, time constraints, and their willingness to pay for the materials required to support the malaria intervention. Using the conceptual framework for implementation fidelity, we determined implementation fidelity through house-level monitoring, using HI coverage indicators, and gathering the community’s perspectives on adhering to HI implementation standards and the quality with which HI was implemented in their villages. Sustainability was determined in this study based on the community’s views regarding noticeable changes through their reflections before and after the introduction of HI, the potential for recommending the intervention to other areas, and whether or not the community would continue implementing HI if external support from MMP were to be terminated. Table 2 shows the study’s outcome variables and data sources under the MMP.

### The conceptual framework for implementation fidelity

**Fig. 2:**
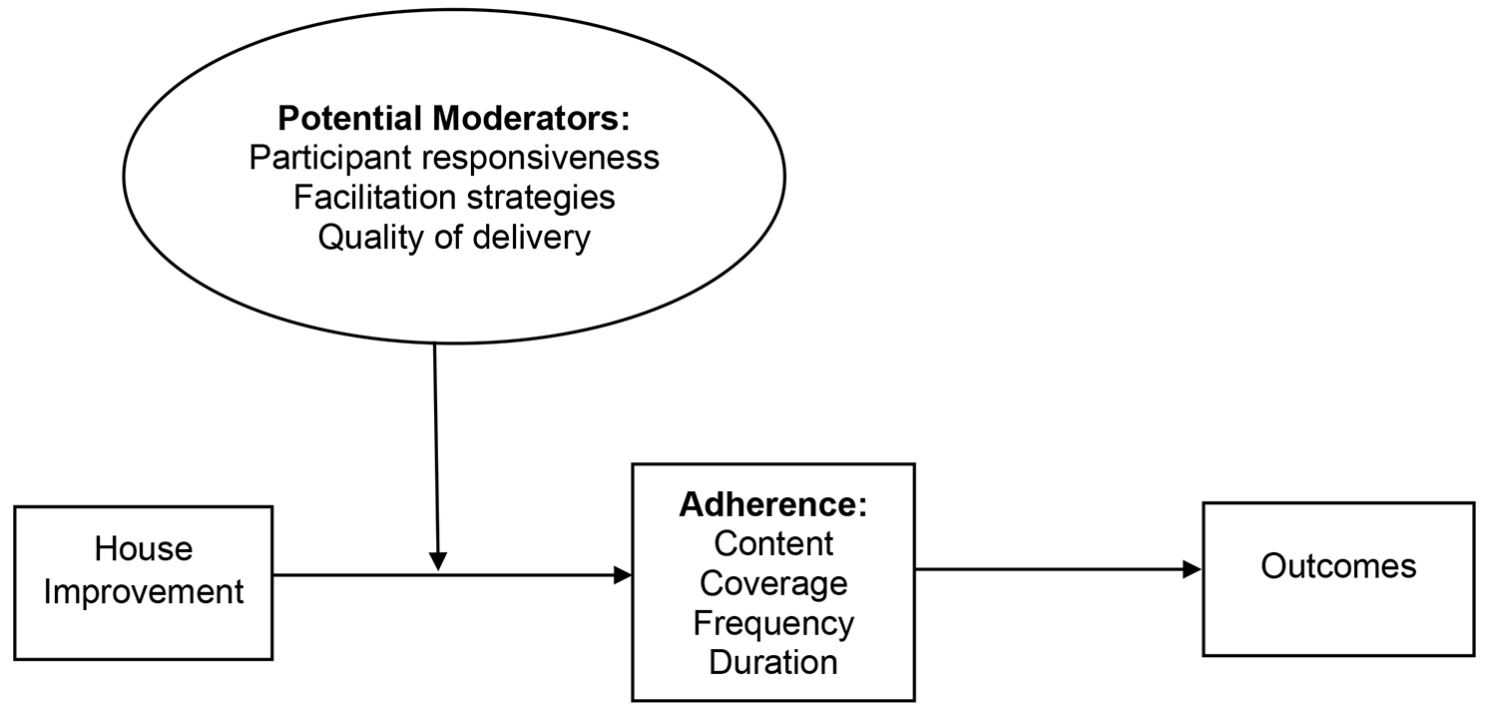
Assessment of fidelity and moderating factors in the present study following the modified version of the conceptual framework for implementation fidelity (originally proposed by Carroll et al.) [28]

To evaluate the implementation fidelity of the HI intervention, we used the conceptual framework based on the work of Carroll et al. [28] and amended by Hasson [53]. This framework was selected to guide the evaluation because it is based on prior work on intervention fidelity and has proved its value [54]. We adapted four common elements of implementation fidelity: adherence to intervention design; exposure to the intervention; quality of delivery; and participant responsiveness [28]. Carroll et al. conceptualised adherence as the main measurement of fidelity, while quality, participant responsiveness, and other elements serve as moderators [28]. We employed this classification in our analysis and interpretation of the findings. This framework is also helpful for evaluating complex interventions [54]. The modified framework is presented in Fig. 2. Using this framework, we defined adherence as the extent to which community-based HI was implemented as it was designed. Adherence has four subcategories namely content, frequency, duration, and coverage. However, these subcategories can also fall under the other elements of implementation fidelity [28, 53, 55]. Employing these subcategories facilitated the assessment of whether the activities were carried out as intended or designed (content) and if both the number of planned activities and the designated area were adhered to (coverage). Exposure was defined as the amount of intervention (community-based HI) received by the participants, encompassing the consistency originally envisioned by its designers and its occurrence over a specific timeframe (frequency and duration). Furthermore, exposure encompassed coverage, indicating the number of households that actively engaged in implementing HI. Quality of delivery refers to how well the stakeholders delivered the intervention. The fidelity assessment used a mixed methods approach employing both qualitative and quantitative methods. In general, this mixed methods approach focuses on implementation research questions that provide insight into whether any programme modifications are necessary before conducting further evaluations and replicating on a larger scale.

**Table 2:** Outcome variables and data sources for the HI evaluation study under the Majete Malaria Project

Outcome	Definition	Approach	Population	Data Sources
Feasibility	The success of implementing an intervention within a specified setting and the extent to which a new treatment or intervention can be successfully carried out	Qualitative	Participants residing in HI villages	**Qualitative** In-depth interviews Key informant interviews Focus Group Discussions
Fidelity	The degree to which an intervention was implemented as intended, planned and designed	Mixed-methods	Participants residing in HI villages	**Quantitative** Household surveys (HI coverage indicators) **Qualitative** In-depth interviews Key informant interviews Focus Group Discussions
HI Coverage (Fidelity Component)	The extent to which a (sub)population that is qualified to benefit from an intervention (HI) actually receives it.	Quantitative	Participants residing in HI and non-HI villages. Involved assessment of the households for various parameters on HI coverage.	**Quantitative** HI Coverage indicators
Sustainability	The extent to which a newly implemented programme or intervention is maintained or institutionalised within a service setting’s ongoing, stable operations	Qualitative	Participants residing in HI villages	**Qualitative** In-depth interviews Key informant interviews Focus Group Discussions

### The sample size for the qualitative survey

The qualitative component of this study included IDIs, KIIs, and FGDs. We chose IDIs due to their depth of understanding of a social phenomenon [56]. We used FGDs to elicit varying participant responses for data triangulation [57]. The IDIs with community members and KIIs supplemented the content of the FGDs. In the study villages, twenty IDIs were conducted with members of the general community. Twenty-three key informant interviews with traditional leaders and HSAs were undertaken in their respective villages or workstations. Nine mixed-village focus groups (involving both men and women) were held with community members, HAs, and HI committee members from various HI villages. These did not include IDI participants.

### Quantitative assessment

A repeated cross-sectional survey sampling framework was employed for the collection of epidemiological data and adult mosquitoes during the trial period (May 2016 through May 2018). These cross-sectional surveys continued until April 2019. During this process, a sample of households was chosen from a demographic database that encompassed the study area every 2 months for the epidemiological survey. In the epidemiological surveys, demographics, malaria control intervention practices and socioeconomic data were collected. In addition to this, HI coverage indicator data were collected.

Therefore, to assess the coverage of HI, the house-level observations included the following: roof type, wall type, window type, floor type, open eaves, number and size of openings, coverage of windows with aluminium screens (gauze wire), and the condition of the door. These observations were conducted by research assistants using a checklist that contained the mentioned variables. This checklist was applied in all sampled houses (both HI and non-HI) during the survey (Additional file 2). These data were used to verify how HI was implemented at the household level and whether the standards outlined by the project had been adhered to over time.

### Sampling strategy

The KIIs, FGD and IDI participants were purposefully selected for the qualitative interviews. The purpose of purposive sampling was to facilitate the identification and selection of participants with ample information (information-rich cases) about the topic of interest. Studying these cases yields insights and in-depth understanding rather than empirical generalisations [58]. Community participants, HAs, and members of the HI committees were purposively selected to participate in the FGDs. Three separate group discussions, one for each of these groups, were arranged within each of the three focal areas. Each group consisted of a minimum of six and a maximum of ten members. The FGDs included group reflections and experiences that shed more light on the fidelity and feasibility of HI as a malaria prevention intervention. The study participants’ perspectives on the sustainability of HI, which had been implemented in their respective villages, were also solicited.

For the quantitative survey, all houses in the study villages were eligible for sampling, irrespective of whether they had been selected in a previous round. In situations where families were large, households could include multiple houses. In each selection round, 270 houses (90 from each focal area) were sampled using a randomised inhibitory spatial random sampling procedure [59]. Inhibitory spatial random sampling for households was preferred over simple random sampling because it enables the achievement of approximately uniform coverage of each of the enumerated focal areas in the study area. This sampling strategy was chosen to ensure that the selection of villages within each enumerated focal area was not biased towards specific geographic or spatial patterns. Furthermore, this strategy helps minimise the potential impact of clustering or uneven distribution of villages, thereby enhancing the overall representativeness of the selected sample. A subset of houses, specifically 195 representing 72%, from the epidemiological surveys, was further randomly selected for adult mosquito sampling [42]. House-level observations were conducted during adult mosquito sampling to determine the coverage of HI (In these scenarios, HI coverage indicators were assessed in all houses where people resided). Detailed descriptions of the epidemiological surveys, adult mosquito sampling, and the process of determining HI coverage are provided in these articles [42, 49]. A total of 14 rounds were conducted during this period. Hence, the estimated sample size for households required in assessing coverage indicator estimates during the 14 rounds of the study was 2730 households. The qualitative assessment was conducted in HI villages only, while the quantitative assessment covered both HI and non-HI villages.

### Recruitment and training of data collectors

The qualitative interviews were conducted by four postgraduates (first, fourth, sixth, and seventh authors) who were research associates, with assistance from six MMP research assistants (Diploma holders). Before the interviews, all data collectors received intensive training for one week under the supervision of the last author. The highly interactive training included an overview of the study, emphasising the study’s primary objective, the study design, qualitative interviewing techniques and strategies, and participants were encouraged to ask pertinent questions throughout. Data collectors were also given consent forms and interview guides in English, which were translated into *Chichewa*, the local language. The research associates did the translation. The consenting process and the use of digital voice recorders were taught to the data collectors. To ensure that the data collection tools were clear, relevant, and comprehensive, they were piloted on individuals in non-intervention villages. Individuals from non-intervention villages exhibited characteristics similar to those from intervention villages.

Questions that were found to be ambiguous were changed, and questions that were found irrelevant to answering the primary study objectives were omitted. The initial interview questions for the various interview participants focused on the participants’ experience, impressions, and challenges in implementing house improvement. Other questions included inquiries related to health problems in the community, the community’s response to malaria, and health promotion. However, questions on the role/influence of the position were restricted to the chief and HSA KIIs because they were technical and pertained to community leadership, whether administratively for the leaders or health-related for the HSAs. Specific questions focusing on the feasibility, fidelity, and sustainability of HI as an intervention for malaria prevention were directed to all the stakeholders (Additional file 3).

### Data collection procedures

Before the interviews, all study participants were scheduled for face-to-face meetings on the specified date, time, and location. The interviews were conducted at various community meeting points. The FGDs, IDIs, and KIIs were conducted privately in a classroom or private room at the community epicentres.^1^ All participants were adults of 18 years and above, of both sexes and different age groups. Administration of IDIs and KIIs lasted about thirty minutes, whereas FGD interviews ranged from 1.5 to 2 hours in duration. All the interviews were conducted in *Chichewa*, the local language. All the potential study participants contacted for qualitative interviews agreed to participate. Forty-seven females and 65 males participated in the FGDs, IDIs, and KIIs. We recorded field notes and then shared and discussed them with the research team when we completed each day’s task. Qualitative data collection took place between 18 March and 20 April 2019.

For the quantitative assessment, the project staff recorded all the HI coverage indicators in a phone-based software called the Open Data Kit (ODK) (Additional files 4 and 5). These data were collected in HI and non-HI villages as part of repeated cross-sectional surveys conducted in randomly selected houses bi-monthly, from May 2016 at the inception of the cluster-randomised controlled trial [48] to April 2019.

### Quantitative data analysis

Demographic data was entered in Microsoft Excel, cleaned, and checked for errors. Data on household characteristics (roof type, wall type, door type, and floor type) were summarised and presented descriptively in tables to capture the different materials used by the community members to construct their houses. The HI coverage indicators were checked for inconsistencies, such as duplicates and missing variables, and cleaned before analysis using a statistical z-test of proportion. Frequencies and associated proportions for each category of variables were analysed to assess the differences between groups (e.g., HI and non-HI Houses) and changes in HI coverage indicators across the years of implementation, using the comparison of proportions. The 95% confidence intervals of the proportions were obtained. Data were analysed using open-source software R version 4.2.0.

### Qualitative data analysis

The audio recordings of the FGDs, IDIs, and KIIs were transcribed verbatim and subsequently translated into English by the first author and research assistants. Field notes were continuously recorded, shared, and discussed with research team members as reflective insights to inform preliminary data analysis during the daily briefing meetings at the end of each day. First, the first author listened to the audio and read the transcripts multiple times to understand the issues raised. The first author familiarised himself with the whole dataset to ensure the data was clean and flowed smoothly. Secondly, we employed thematic analysis to analyse the data. The first author coded the transcripts sent to the last author for comment and agreement on a common coding framework. A codebook was developed using inductive and deductive coding methods (Additional file 6). The inductive approach is bottom-up with codes derived from the data, i.e., using the participants’ words to code the data (Additional file 7). At the same time, the deductive method was based on a predefined set of principles, which guided the coding process [60, 61]. The translated excerpts were coded using Nvivo 12 Plus (QSL International, Victoria, Australia), (Additional file 8). Key themes in the coding framework included the feasibility, fidelity, and sustainability of community-led HI. All audio recordings and transcripts were saved in a password-protected computer, with the researchers only granted access. The chosen quotes represented the most comprehensive feedback on the topic. Input from different stakeholders was incorporated into that theme to create a balanced representation of the quotes.

We evaluated implementation fidelity by drawing on our quantitative and qualitative data to assess content adherence, the facilitation strategies to support the implementation of HI (the usefulness of the training workshops and the training manuals), and the quality of delivery (whether the intervention was delivered appropriately to achieving what was intended) and finally participant responsiveness (stakeholder views on the relevance of the intervention) [28]. Sustainability was inferred from participants’ responsiveness to the intervention and their opinions about maintaining HI when the project is closed out.

### Data integration

Although most data sources in this study were qualitative, equal importance was placed on both data types. The researchers collected, analysed, and combined quantitative and qualitative data simultaneously to obtain a comprehensive understanding of the level of fidelity achieved and to enhance the credibility of their findings and inferences [62, 63].

### Ethical considerations

Before study implementation, the University of Malawi’s College of Medicine Research and Ethics Committee granted ethical approval (COMREC protocol numbers P.07/18/2442 and P.05/15/1731). The Chikwawa District Health and Social Services (DHSS) office permitted data collection in the study villages. Before recruiting participants, we communicated the study objectives through local village heads liaising with HAs. Written informed consent was obtained from all participants during data collection. All the participants were men and women aged 18 years and above.

## Results

### Socio-demographic characteristics of participants

One hundred twelve (112) participants participated in the 52 interview sessions: 43 IDIs and 9 FGDs (Table 3). Three FGDs were conducted per focal area: community members, HAs, and HI committee members. Most participants were aged 25 to 44 (58.0%) and reported primary education as their highest level of formal education (51.8%). More males (58.0%) than females (42.0%) participated in the interviews.

**Table 3:** Demographics of study participant

Characteristic	Focal Area (n)			Total Participants [n, (%)]
	Focal area	Focal area	Focal area	
	A (39)	B (32)	C (41)	112 (100.0%)
**Gender**				
Male	25	19	21	65 (58.0%)
Female	14	13	20	47 (42.0%)
**Age**				
18–24	8	8	9	25 (22.3%)
25–44	21	18	26	65 (58.0%)
≥ 45	10	6	6	22 (19.6%)
**Education**				
None	21	10	4	35 (31.3%)
Primary	13	14	31	58 (51.8%)
Secondary	5	8	6	19 (17.0%)
Tertiary	-	-	-	-
**Session**				
FGD	3	3	3	9 (17.3%)
IDI	6	3	11	20 (38.5%)
KII	10	5	8	23 (44.2%)

### Characterising house structures in HI villages and non-HI villages

During the 12 rounds in the trial period (May 2016 through May 2018) and the 2 rounds in the post-trial period (September 2018 through April 2019), a total of 3613 households (3240 during the trial and 373 post-trial) were selected for epidemiological surveys. Households were sampled from all villages during the study period, and some were selected multiple times in different rounds. A total of 2056 unique household visits occurred during the study period (1844 during the trial and 212 post-trial). Only the unique household visits were included in the analysis. These visits encompassed households that were substituted by the nearest neighbour when the initially selected household was absent. Out of the 3613 households selected for epidemiological surveys, 2717 (75.2%) were chosen for adult mosquito sampling and assessment of HI coverage indicators. These were included in the analysis presented here. Table 4 below shows house characteristics in HI and non-HI houses obtained through the quantitative assessment. There were 1128 HI households visited as shown in Table . In these villages, 55.4% of the households had closed eaves on all four sides. There were 1589 non-HI houses visited where 27.1% of the houses had eaves closed on all four sides. In HI villages, 73.6% of houses had windows screened with aluminium mesh, whereas, in non-HI villages, 6.7% of houses had aluminium mesh window screening. The presence of screened windows in non-HI villages was primarily a proactive measure taken by household owners. This initiative was independent of the MMP, as the MMP did not provide materials or support for window screening in these areas. The household owners utilised their own local materials and resources for this purpose, and the screening was implemented after the trial was already in place. Furthermore, 91.7% of the HI houses used wood as door material and 87.8% of non-HI houses. Most HI and non-HI houses used natural material for roofing, at 64.5% for HI houses and 64.8% for non-HI houses. Around 71.4% of HI houses and 68.3% of non-HI houses had fire-baked brick as the wall material. 87.6% of HI houses used mud/sand/dung material for flooring compared to 89.6% of non-HI houses (Additional file 9).

**Table 4:** House characteristics in HI and non-HI houses

House Characteristics		Total		Total
		HI		Non-HI
**Houses visited**		1128		1589
	Number of Houses	Proportion positive (95% CI)	Number of Houses	Proportion positive (95% CI)
**Eaves**				
Houses that closed their eaves (i.e., cumulatively but to varying extent)	831	0.74 (0.71, 0.76)	793	0.50 (0.47, 0.52)
Houses with eaves completely closed on all 4 sides	625	0.55 (0.52, 0.58)	430	0.27 (0.25, 0.29)
**Windows**				
Houses with windows	972	0.86 (0.84, 0.88)	1215	0.76 (0.74, 0.79)
Houses with no windows	156	0.14 (0.12, 0.16)	374	0.24 (0.21, 0.26)
Houses with windows that can be closed*	791	0.81 (0.79, 0.84)	707	0.58 (0.55, 0.61)
Houses with screened windows with gauze wire*	715	0.74 (0.71, 0.76)	82	0.07 (0.05, 0.08)
**Door Material**				
Wood	1034	0.92 (0.90, 0.93)	1395	0.88 (0.86, 0.89)
Reed	85	0.08 (0.06, 0.09)	181	0.11 (0.10, 0.13)
No Covering	3	0.003 (0.0005, 0.008)	12	0.008 (0.004, 0.013)
Other material	6	0.005 (0.002, 0.01)	4	0.003 (0.001, 0.006)
Houses with doors containing spaces	429	0.38 (0.35, 0.41)	500	0.31 (0.29, 0.34)
**Roof Material**				
Natural material	727	0.64 (0.62, 0.67)	1029	0.65 (0.62, 0.67)
Iron Sheets	399	0.35 (0.33, 0.38)	556	0.35 (0.33, 0.37)
Iron and Tiles	2	0.002 (0.0002, 0.006)	4	0.003 (0.001, 0.006)
Cement	0	0 (0)	0	0 (0)
**Wall Material**				
Mud/dung	158	0.14 (0.12, 0.16)	116	0.07 (0.06, 0.09)
Sun-dried brick	160	0.14 (0.12, 0.16)	384	0.24 (0.22, 0.26)
Fire-baked brick	805	0.71 (0.69, 0.74)	1085	0.68 (0.66, 0.71)
Iron Sheets	2	0.002 (0.0002, 0.006)	1	0.0006 (0, 0.004)
Wood	0	0 (0)	1	0.0006 (0, 0.004)
Other material	7	0.006 (0.003, 0.01)	1	0.0006 (0, 0.004)
**Floor Material**				
Dirt/mud/sand/dung	988	0.88 (0.86, 0.89)	1425	0.90 (0.88, 0.91)
Wood/ Plank	2	0.002 (0.0002, 0.006)	6	0.004 (0.001, 0.008)
Cement	137	0.12 (0.10, 0.14)	157	0.10 (0.08, 0.11)
Tiles for main floor	1	0.0009 (0, 0.005)	1	0.0006 (0, 0.004)

*Excluding houses with no windows

### Adherence of standards to HI implementation

Table 5 presents the results regarding the standard of HI implementation over the three years from 2016 to 2019. We evaluated adherence to each component separately, including closing eaves, screening windows, and fitting doors. This approach enabled the analysis and understanding of adherence to each specific HI component independently, providing a more detailed and nuanced assessment of compliance with the intervention. There was a total of 510 house visits in the villages implementing HI in 2016-17, 456 in 2017-18, and 162 in 2018-19. The small sample size in 2019 was attributed to heavy rains and floods that affected households. In 2016-17 and 2017-18, 378 and 337 houses, respectively, closed their eaves to varying degrees (i.e., there was a clear attempt by the household owners to close the eaves), representing 74%. However, there was a decline in 2018-19, with only 70% of the houses closing their eaves. In 2016-17, 42% of houses had completely closed eaves on all sides. In 2017-18, this increased to 48% of houses having their eaves completely closed. However, in 2018-19, there was a decline, with only 33% of houses completely closing their eaves on all sides. Of note is that there has been a decrease in various parameters over the years. There was a slight increase in the number of houses with open eaves over the years. In 2016-17, 56 houses, representing 11%, had eaves open on all four sides. In 2017-18, this number increased to 57 houses, representing 16%, with eaves open on all sides. In 2018-19, 16% of houses had eaves open on all sides. Regarding window screening with gauze wire, in 2016-17, 72% of the houses screened their windows with gauze wire. This percentage slightly decreased to 71% in 2017-18 and further dropped to 57% in 2018-19. A similar pattern emerged when considering houses with door spaces. In 2016-17, 39% of houses had door spaces, while this decreased to 36% in 2017-18, and then increased to 42% in 2018-19.

**Table 5:** Houses with HI implemented according to standard

				Year		
		2016–2017		2017–2018		2018–2019
**Number of HI houses visited**		510		456		162
**Characteristic**	Number of Houses	Proportion positive (95% CI)	Number of Houses	Proportion positive (95% CI)	Number of Houses	Proportion positive (95% CI)
**Eaves**						
Houses that closed their eaves (i.e., cumulatively but to varying extent)	378	0.74 (0.70, 0.78)	337	0.74 (0.70, 0.78)	113	0.70 (0.62, 0.77)
Houses that did not close their eaves	132	0.26 (0.22, 0.30)	119	0.26 (0.22, 0.30)	49	0.30 (0.23, 0.38)
Houses with eaves completely closed on all four sides*	158	0.42 (0.37, 0.47)	163	0.48 (0.43, 0.54)	38	0.34 (0.25, 0.43)
Houses with eaves closed but with gaps 10 cm or more*	87	0.23 (0.19, 0.28)	84	0.25 (0.20, 0.30)	16	0.14 (0.08, 0.22)
Houses with eaves closed but with gaps 5–10 cm*	39	0.10 (0.07, 0.14)	30	0.09 (0.06, 0.12)	14	0.12 (0.07, 0.20)
Houses with eaves closed but with gaps 1–5 cm*	59	0.16 (0.12, 0.20)	41	0.12 (0.09, 0.16)	22	0.19 (0.13, 0.28)
Houses with eaves partly closed, e.g., one side of the house^†^	35	0.09 (0.06, 0.13)	19	0.06 (0.03, 0.09)	23	0.20 (0.13, 0.29)
Houses with eaves open on all four sides^†^	56	0.11 (0.08, 0.14)	57	0.13 (0.10, 0.16)	26	0.16 (0.11, 0.23)
**Windows**						
Houses screened with gauze wire (Excluding those without windows)	314	0.72 (0.68, 0.77)	284	0.71 (0.66, 0.76)	87	0.57 (0.49, 0.65)
**Doors**						
Houses with doors containing spaces	201	0.39 (0.35, 0.43)	167	0.36 (0.32, 0.41)	70	0.42 (0.34, 0.50)

### Qualitative assessment

Table 6 below summarises the key themes drawn through the inductive and deductive methods emerging from the study: the fidelity of HI implementation by the local community, the feasibility of HI implementation by the community, and the sustainability of HI as an intervention for preventing malaria.

**Table 6:** Main themes from the qualitative study

Themes	Sub-themes	Researcher’s interpretive summary
Fidelity of HI implementation by the community	Content adherence Quality of delivery	Stakeholders’ perspectives on the standard of HI implementation and activities carried out as planned Stakeholders’ perceptions of the quality of HI houses within their villages
Feasibility of HI implementation by the local community	Barriers and enablers to implementing HI Stakeholder responsiveness to the intervention	Participant views on barriers and enablers to implementing HI Stakeholders’ perceptions of the interest, need, and commitment to receiving or delivering HI
Sustainability of HI as an intervention for preventing malaria	Facilitation strategies Significant changes experienced through HI implementation Continuation of HI duties by various players Future recommendations on HI	Stakeholders’ perspectives on strategies implemented to improve fidelity Participants’ positive or negative experiences and opportunities available for sustainability The willingness to continue to implement HI. Community members’ ideas on how best HI could be implemented and rolled out into other areas

### Fidelity of HI implementation by the local community

Under this theme, the fidelity of implementing HI revealed the intervention’s complexity. Overall, HI was implemented throughout the designated villages within the MWR perimeter. Community stakeholders were all involved in implementing and promoting HI. There was good adherence to the intended HI implementation. However, as shown in Table 5 and explained by community stakeholders, adherence declined over time. In other words, achieving high implementation fidelity was influenced by several factors outlined below. Firstly, the workload required to close the eaves in the houses; secondly, the availability and affordability of building materials; and thirdly, the quality and timely supply of gauze wire. Additionally, adverse weather conditions affected the HI implementation during the trial period, disrupting supervision and posing challenges for both HI committee members and HAs to assess implementation fidelity.

### Content adherence

The essential elements of the HI intervention were delivered as planned. However, there were variations in adherence to the standard within the villages. The community-taught delivery standard included complete eave closure, screening windows with gauze wire, and closing all small spaces/gaps on the wall, door, or roof. Members of the HI committee, whose primary role was to educate fellow community members and lead the implementation process, had the general impression that people followed and adhered to the recommended HI standards, even though adherence differed across houses.

*“People adhere to the standards because we have taught them well. When the mud used to close eaves and gaps in most houses begins to crack, we tell them to repeat the process. After repeating this procedure, we instruct them to enter the house and check for open spaces. When we go to the villages for supervision, we find what is supposed to be done.”***(FGD, HI committee, FA-A)**.

In contrast, some participants believed that adherence needed to be consistent and observed significant variation across communities. Some of the challenges reported with compliance were that the task was laborious, and the materials needed to be more affordable and easily accessible.

“*This work, like any other, is laborious. Generally, the type of house usually determines the extent of the work. Some houses have a lot of open spaces that were left unfinished when the house was built. As a result, when you tell owners to close the eaves and other gaps, they become reluctant because they will have to make bricks and fetch for other materials*.” **(KII, HSA, FA-A)**.

*“Some HI materials are difficult to access by most people. For example, they may have to travel long distances to obtain a tool such as a shovel. Such materials are not available to everyone but only to builders. It is sometimes difficult because the builder uses it when you want to borrow.”***(FGD, HA, FA-A)**.

“*We cannot afford to buy gauze wire on our own because several issues plague our community. The first is a serious hunger problem in our community, and second, it isn’t easy to earn money. Hence people’s priorities would be on the more serious issues they are facing, so as it is, we cannot afford to buy gauze wire on our own. It is expensive.*” **(IDI, Community participant, FA-C)**.

Other factors that significantly impacted fidelity and adherence included weather conditions. Strong winds and heavy rains disrupted the implementation process, leading affected houses to redo the implementation. As demonstrated in Table 5, there has been a decline in HI implementation over time, which could be attributed to the necessity of redoing the exercise due to issues caused by natural disasters. Certain areas became inaccessible during the rainy season, making the distribution of materials and supervision challenging.

*“The houses are being improved. However, we should not hide that many houses were destroyed during the heavy rains in 2019. So, we cannot say that houses are not properly improved when, for example, a brick house with a collapsed wall resulting from a disaster is replaced with a grass-thatched wall. Different designs determine the quality of the HI.”***(FGD, Community participants, FA-A)**.

Secondly, the presence of various house designs within the community posed challenges in adhering to a uniform standard. Not all houses were built using the same materials, such as bricks, concrete, and others. The utilisation of diverse materials in house construction became a factor influencing the standard and quality of HI across different villages. As indicated in Table 4, walls, roofs, and doors were constructed using different materials, largely determined by affordability.

*“There are houses we do not have a problem with, especially those with roofs made of corrugated iron sheets. When HI is implemented in such houses, the condition is excellent unless something terrible occurs, such as natural disasters, including strong winds. However, grass-thatched houses tend to have open spaces that must be maintained regularly. These are the types of houses that give animators so much work when it comes to monitoring HI.”***(FGD, HA, FA-B)**.

Thirdly, pets and domestic animals within households were mentioned as another reason for poor adherence to quality and standards. For instance, one health animator stated the following:

“*Some houses were greatly improved in terms of eaves closure. However, some had not improved. Some people stated that there were issues with animals, such as goats getting into the house. When they planned to chase the goats inside the house, they would escape through the screened window, damaging the gauze wire.”***(FGD, HA, FA-B)**.

### Quality of HI

When participants were asked about their perception of the quality of HI houses within their villages, they had the following to say:

*“We are pleased with the HI quality in our village. Following implementation, the HI committee visits for spot checks. They look around the house to see if there are any open spaces. They inspect that HI has been done correctly, and the committee approves the house when there are no spaces. If there are still open spaces in a house, the committee instructs the owners to close the spaces properly.”***(FGD, Community participants, FA-A)**.

However, some HSAs reported poor implementation of HI because of how some windows are installed, which compromised the quality.

*“Some houses have poor HI quality, perhaps because of the designs of the windows. There are houses with windows that open from the inside, while others open from the outside. So, it is difficult to install gauze wire. Despite this, they still install the gauze wire.”***(KII, HSA, FA-B)**.

Other issues raised concern the quality of the gauze wire material, which compromised the delivery of the intervention. For example, most stakeholders in the community said that the materials supplied to support those implementing the intervention lacked durability and longevity. In addition, the inconsistent and unpredictable supply of materials was a source of the complaint.

*“The concern is that the first gauze wire we received did not last long. It was so easily damaged by rust. It takes longer to send a report that we have run out of gauze wire. People complain because they are not safe during this time. When you receive a report that the gauze wire is needed, try your best to consider our requests as soon as possible to address the people’s concerns in the villages.”***(FGD, Community participant, FA-B)**.

### Feasibility of HI implementation by the local community

#### Barriers and enablers

Feasibility was inferred from stakeholder views on barriers and enablers to implementing HI as a malaria control intervention. Some members of the community reported that the work was relatively easy. They emphasised collaboration among all partners to make the workload manageable.

“*What we could do to make this work easier is work together, and everyone must participate. Both the HI committee member and the animator must participate. Then the work will be easier. If there are problems, we must determine what caused those problems and address the problem. If these issues are addressed, everything will be fine*.” **(FGD, HA, FA-B)**.

The participants were also able to identify alternatives for resources that were not easily accessible.

“*Firstly, the owner of the house needs to look for bricks. After the bricks have been organised, the eaves can be closed, and we must ensure that the walls are in contact with the roof. We also need mud to close the eaves. We need nails to secure the gauze wire to the windows. As an alternative to nails which could not be easily accessible, bamboo or reeds can be used to secure the gauze wire to the window frame so that it does not blow away due to strong winds*.” **(FGD, HA, FA-C)**.

Challenges with the implementation of HI included the personal sacrifice that people had to make to reconstruct their houses and particularly how the implementation of HI disrupted their primary tasks, such as farming.

“*HI work has prevented us from performing our daily duties, but as volunteers, we were determined to complete it because the intervention came to our villages. Numerous activities have been impacted, but we will only mention a few, such as farming. We attempted to balance them so that we would do farming in the morning, and in the afternoon, we would do the other work*.” **(FGD, HI Committee, FA-B)**.

### Stakeholder responsiveness to HI

Stakeholder responsiveness was evaluated by participants reporting on enthusiasm, commitment to receiving or delivering the intervention, and how far all stakeholders perceived the intervention to be helpful. The community believed that HI contributed to reducing malaria cases and related mortality. Another change that the health animators noticed was a change in attitude among community members. Initially, most community members had been antagonistic towards HI and other malaria prevention methods. Furthermore, the role played by the community volunteers in leading the implementation of HI and educating and training the communities helped foster trust with the community stakeholders.

“*The main difference is that previously, many people suffered from malaria regularly. Furthermore, the majority of the people were suffering from severe malaria. When some people begin to feel sick in the morning, they lose consciousness by the afternoon. However, with the advent of the house improvement initiative, most malaria cases are now mild*.” **(FGD, Community participants, FA-B)**.

“*People’s attitudes have changed, so there has been progress in my village. Previously, people had negative attitudes toward the use of mosquito nets. They now recognise the value of sleeping under a mosquito net and having HI, thus acting responsibly. When they attend malaria village meetings, they ask and answer questions and are very interested in what is happening there.”***(FGD, HA, FA-C)**.

*“I was explaining that before HI was introduced, we used to suffer from various diseases because mosquitoes would bite us. It was possible to wake up in the morning with swellings, indicating mosquito bites. Since HI was introduced, there has been a significant change. Now, most people understand what the HI committee is saying, including myself; I now understand what the committee says and trust that it’s true. I have also noticed some changes; there is a difference compared to the past. The village is changing.”***(KII, Chiefs, FA-A)**.

There was a widespread perception among the participants on how to get the community to be fully involved in the ongoing HI activities. Enthusiasm and commitment were demonstrated when some participants reported that they intend to inspire others in other villages to undertake this intervention by teaching and training them about the advantages of HI. They had the following to say:

“*I would encourage them that house improvement is very good and simple to implement. Once the HI committee mentions that they want to improve your house, it takes some time because the HI committee contains many people. Just encourage the people to accept so they do not frequently suffer from malaria.*” **(IDI, Community participants, FA-C)**.

“*We can encourage people from other villages where the intervention will be scaled up to understand the importance of closing eaves and installing gauze wire on windows. They mostly would be unaware because the intervention would be new to them. They will teach the people in their village what they learned from me. We can educate the people by asking the chief to gather them and then explain the significance of installing gauze wire on windows and closing eaves*.” **(FGD, Community participants, FA-B)**.

### The sustainability and the scalability of community-led HI

#### Facilitation strategies

Training workshops for HAs and HI committee members, training manuals to improve knowledge on malaria and HI as a prevention tool, and community sensitisation and engagement meetings were implemented to optimise fidelity.

Community sensitisation meetings and education were powerful tools to get ideas across. Most HAs and HI committee members mentioned that offering education through village workshops and community sensitisation meetings helped their communities change their thinking.

*“Through sensitisation, with support from the Epicentre Project Officer, health surveillance assistants and animators from other villages, people understood what the intervention was all about”***(FGD, HA, FA-C)**.

Establishing HAs and HI committees as advocates for HI was another valuable strategy to achieve fidelity. However, there are times when the HAs and HI committees reported that they encountered resistance and uncooperativeness from the community. Such acts prompted them to seek intervention from the chiefs, who are the gatekeepers in the community. Chiefs knew several hiccups occurred in the HI implementation process over the years. They echoed a need for constant community engagement to mitigate the problems or adapt to the implementation process.

*“Do not give up on the residents of certain villages if delivery of HI standard is poor. Visit them, educate and enlighten them on the importance of house improvement using the materials provided, and demonstrate how it is done. Discuss with them what needs to change or not. They will fall into line and be able to follow in this manner.”***(KII, Chiefs, FA-B)**.

Some health animators did suggest that having model houses built to standard would have also helped set the standard for the community to construct quality houses that prevent mosquito entry.

“*People in the village will use the demonstration house as an example of how their houses should be improved after seeing it. We made certain that the demonstration houses belonged to committee members because we wanted the houses to be accessible and to belong to someone who was familiar with the project and could improve their houses accordingly.*” **(FGD, HA, FA-C)**.

We solicited views from the participants on the possible recommendations for the scale-up of community-led HI into other villages. The study participants expressed varied opinions in the discussions. Most believed collaboration among all community stakeholders was essential to ensuring a smooth intervention rollout.

“*As communities implementing HI, we could first request to meet with the chiefs before meeting with the rest. Our main message would be that there is a need for collaboration among all stakeholders in the community, including leaders, to ensure the successful implementation of HI*.” **(FGD, HI Committee, FA-B)**.

*“We need to encourage people from other villages where HI will be rolled out in the future to understand the importance of closing eaves and installing gauze wire on windows. They may be unaware because it will be a new intervention. Another consideration is that the animators that will be chosen in such villages need to be well-organised. For example, they may meet someone like me, and I can converse with them if I am knowledgeable enough. They can teach the people in their village after I teach them. They can educate the people by asking the chief to gather the people and then explaining the significance of HI.”***(FGD, Community participants, FA-B)**.

HSAs and chiefs stated that they could sustain the programme by supplementing the efforts of HAs and HI committees. One leader remarked that the initiative was unique because many village members were involved. The community participants’ capacity was built at various levels, emphasising malaria prevention and control significance. This resonated well with the community, which experienced a significant reduction in the malaria burden in the area. The following sentiments were expressed:

“*As a chief, I am confident HI is here to stay. Other projects have previously been implemented in my village; for example, a certain organisation introduced an aquaculture project with only a few community members benefiting from the training. Ponds were built in the village, but as I speak, the organisation left, all donor support was seized, all project-related activities were halted, and the ponds are now empty and without fish*. *This is not the case with HI, where everyone is involved.*” **(KII, Chiefs, FA-B)**.

Health surveillance assistants expressed their hope that HI would continue. Their observation has been that there has been a reduction in malaria cases in the villages they supervise, as evidenced by malaria data collected from village clinics and health facilities.

“*I believe that HI will continue regardless of whether or not the project receives external funding. This is because residents in the community I supervise have seen the value of having this intervention. As an HSA who compiles data for various health indicators in the communities where I provide support, I have noticed a drop in malaria cases since these activities were implemented. This situation differs from what we saw 2–3 years ago before HI.*” **(KII, HSA, FA-A)**.

## Discussion

This is the first study in Malawi to evaluate the implementation fidelity, feasibility, and sustainability of training volunteers and local community stakeholders implementing a community-led HI strategy. HI was implemented as a complementary intervention for malaria control in southern Malawi. The study provides evidence supporting the practical use of trained volunteers to engage communities in malaria control efforts. The research context highlights the feasibility of involving HAs and HI committee members (HA model) as advocates for the intervention, leading to high levels of fidelity. As shown in previous studies, it is feasible to introduce health animation in rural setups [51]. However, it is important to recognise that the success of implementing the programme is strongly influenced by several factors, such as the population’s characteristics, support from community leadership, training, and provision of necessary educational and construction materials for the volunteers. These factors support implementation fidelity, feasibility and impact on sustainability. We discuss these elements below.

### Factors supporting fidelity in HI implementation

Fidelity assessment was relevant given the need for existing programmes to study adherence to the designed intervention to determine whether the intervention was implemented as planned; understanding this level of fidelity was required before the results could be attributed to the intervention and its effectiveness confirmed [33, 64]. Like previous research [65–68], the quantitative and qualitative results aligned to demonstrate how various factors can affect implementation fidelity.

Several factors came together to support the implementation of HI. Firstly, the implementation and maintenance of HI fidelity in preventing malaria were facilitated by training workshops for HAs and HI committee members and the availability of training manuals to improve their knowledge on the subject. Secondly, community sensitisation and engagement meetings were instrumental in achieving these goals. Initial and subsequent refresher training facilitated by the MMP was conducted with the community volunteers. Usually, training end-users motivates providers to deliver services with high adherence or fidelity [69]. The design of the intervention and its implementation strategy through the MMP was well-established. It involved creating training modules, providing manuals, and conducting regular follow-up meetings through village workshops. These approaches contributed significantly to the implementation process. Therefore, although complex interventions tend to have a lower level of fidelity [70], the methods used under the MMP alongside our study findings demonstrate that maintaining fidelity is achievable when programmes are firmly rooted in communities and implementers understand their role and the reasons for HI for the control of malaria [71].

Thirdly, the characteristics of implementers (HI committees, HAs), especially their proficiency, expertise, motivation, and understanding of the intervention, facilitated implementation and helped maintain fidelity. Establishing these volunteer platforms strengthened community sensitisation for the use and acceptance of HI. This is consistent with previous studies highlighting community groups’ crucial role in promoting accurate knowledge about malaria control and healthcare utilisation [72, 73]. Additionally, since these individuals were residents of the same community, it was effortless to establish a rapport with the community, fostering trust. The importance of trust among community groups, as found in this study, corroborated that of a study conducted in Blantyre, southern Malawi, where implementing the volunteer system fostered trust among the volunteers and within the community [74]. However, careful reflection must be considered when utilising a system involving volunteers, particularly when these volunteers hold varying hierarchical positions within the study or intervention implementation. This is crucial because tensions and conflicts may arise, potentially compromising the sustainability of the community-based intervention [74]. During the study conducted in Blantyre, a situation emerged where tensions and conflicts arose because of imbalanced power dynamics among the volunteers. Specifically, one group assumed a watchdog role over the other, leading to a deterioration of trust [74]. This situation had negative implications for the volunteers’ well-being and social relationships. Trust plays a crucial role in health systems and development as it forms the foundation for cooperation within the system, vital for promoting health and building a strong society [75].

Community leadership, particularly through the village chiefs, is known to significantly influence the success of such interventions [76–78]. The volunteers recognised the chiefs’ impact as crucial in motivating community members to engage in HI activities. Community leaders’ participation effectively addressed the issues related to HI implementation beyond the volunteers’ capacity. This finding mirrors findings in a study conducted in the same area and within southern Malawi, where local authorities successfully built trust in the volunteers and impacted the community during the study implementation [74, 79].

### Factors affecting fidelity in HI implementation

Community members were aware of the value of an improved house in reducing the risk of malaria transmission. However, this study found that the quality of community-led HI implementation had declined with time. Overall, we found that the first two years had better content adherence to the intended HI implementation strategy than the third year (2018–2019) of the implementation fidelity of HI and found varying degrees of commitment.

Findings from this study further showed the socioeconomic difficulties associated with implementing community-led projects that rely on volunteer participation. A notable example of these challenges is the coexistence of humans and domestic animals within the same household, illustrating community residents’ economic constraints in managing human and animal dwellings. Additionally, the volunteers’ work on HI was unremunerated and conflicted with the volunteers’ other chores at the family and community levels and potential paid employment. These elements affected the programme’s fidelity and posed significant doubts about its long-term viability. These findings correlate with previous research in the same area and other parts of sub-Saharan Africa, where a lack of financial compensation and non-financial material incentives have been reported to impede the delivery and sustainability of volunteer work [50, 79–86].

Furthermore, other socioeconomic and contextual factors were found to affect the fidelity of HI implementation. The implementation of HI was considered labour-intensive and time-consuming, impacting the regular duties and activities of certain stakeholders. HI activities, such as closing eaves and fixing gauze wire on windows, had to be repeated over the years due to the destruction of some houses by natural disasters such as heavy rains (floods) and strong winds. The destruction of these houses was primarily attributed to the use of building materials, such as mud, that lacked durability. People construct houses using various materials based on their financial situation. Moreover, the gauze wire procured by the project at the inception of the trial exhibited signs of corrosion in some locations. A magnet test confirmed the presence of iron in the gauze wire [49]. By February 2017, corrosion had led to broken screens in several houses, mainly due to the rains and unfavourable humid conditions within the first two years of the trial [49]. These results are also consistent with a study on LSM conducted in the same area, which demonstrated that some reasons for reduced participation and adherence to LSM activities were the labour and time demands associated with those activities [50].

These findings underscore the importance of incorporating technical solutions that would improve the quality and uptake of the intervention while decreasing labour demands. Among the technological solutions are eave tubes, which have also proven to be cost-effective [87–89]. Furthermore, utilising non-corrosive aluminium wire gauze is a better solution, and the MMP adopted this type of wire mesh by June 2017 [49]. In the longer term, when additional organisational resources become available in LMICs, the involvement of government sectors, such as lands and housing, in housing and infrastructure developments in rural communities will be crucial. At a minimal extra expense, upcoming infrastructure and housing initiatives can be planned, designed, and developed with vector control as a central consideration [90].

### Barriers and enablers to the feasibility of HI implementation

Collaboration among community stakeholders was cited as an enabler in ensuring the HI activities were done accordingly. In this study, non-specialist local community volunteers (HAs and HI committee members), who received theory and practical training from the MMP, could autonomously and regularly lead in implementing HI, working consistently with their community counterparts and existing community leadership structures. Within this setup, a collaborative network was formed. This platform helped strengthen stakeholder relationships to foster mutual support and joint ownership of solutions. It enabled the exchange of ideas and collaborative problem-solving among all stakeholders, ensuring the work was feasible and successful in HI implementation. The findings are consistent with previous community-based malaria control studies that demonstrated the importance of active community participation in creating a conducive environment for intervention ownership and knowledge utilisation [73, 91–94].

However, the feasibility of HI implementation could have been improved by the perceived barriers of the high cost associated with materials like gauze wire and the disturbance of work and daily routines caused by HI activities. Costs of HI are justified in a separate study within the same setting [95]. The expenses of community-led HI for malaria control were higher than in previous house improvement studies [95]. This finding implies that the current strategy for implementing HI could be more expensive, which may pose challenges in terms of sustainability. Capacity building of local communities and the availability of essential tools and materials are required to enable the local communities to carry out their malaria control programmes [96, 97]. These need to be considered when considering feasibility issues.

### Conditions for sustainability

The study proves that the HA model can effectively convey information, education, and communication (IEC) to supplement malaria control interventions. This finding is consistent with a study conducted in the same area that investigated the experiences of being a HA in a rural setting [79]. To enhance comprehension of the intervention and address concerns among communities using HI as a potential tool for malaria control in Malawi, it is essential to provide targeted messages and health education. By focusing on specific messaging and educational approaches, we can foster a better understanding of HI and alleviate any apprehensions held by these communities. The development of educational methods should be considered for advocating HI as a complementary approach to malaria control in Malawi.

The general perception among study participants was that community-led HI could be sustained, but this depends on active community participation. Community participation, including establishing structures such as village committees, has been recognised as an essential strategy for effective community-based programmes [98]. One unique feature of the MMP project was the collaborative partnership between the research team and THP. The Hunger Project’s epicentre strategy supported and facilitated community participation and ownership. Collaborative approaches like these boost commitment and programme sustainability [99]. This approach aligns with the World Health Organization’s (WHO) recommendations. The WHO emphasises the significance of identifying strategies that are acceptable to the people they affect and can be seamlessly integrated into their daily lives and community structures [100]. This approach is crucial for achieving effective community participation.

Although programme models may exist, fidelity levels lower than 100% may require some adaptation of the intervention to the local context [28]. Our findings reveal that although some of the materials needed for implementing HI were expensive or not readily available, the community found ways to substitute the materials by using locally available resources that were easily accessible and affordable for the community. Their willingness to improve their dwellings demonstrates a commitment to reducing their risk of malaria, as some authors argue that when communities make such adaptations to interventions, it improves the chances of success [35]. A relevant example comes from Rwanda, where Ingabire et al. reported that adapting interventions led to enhanced community acceptance and utilisation of vector control strategies and improved coverage of community-based health insurance [73]. These findings further emphasise the significance of active community participation through volunteer platforms, which create an enabling environment for programme ownership and knowledge utilisation at both individual and community levels [73, 91, 93, 94].

In our case, HI could be slightly modified using cheaper and locally available materials to ensure it is well implemented. Previously used ITNs could be a viable option for window screening to improve the community uptake of the intervention, given its cost-effectiveness and suitability for the current context [101, 102]. Other items, such as polyvinyl chloride (PVC) coated fibreglass netting material, could also be a feasible alternative for enhancing the acceptance of the intervention within the community, considering their affordability and compatibility [8, 18, 103–15].

### Strengths and limitations

This study highlights the significance of utilising a mixed-method design to evaluate implementation fidelity, especially when dealing with the complexity of public health interventions [25, 66, 105]. The triangulation of qualitative and quantitative data sources, along with the rigorous methodology used for data analysis, resulted in a deeper understanding and enhanced the internal validity of our findings. Additionally, the study’s participatory approach fostered an environment of rapport and trust during the interviews. The credibility of the qualitative component was further enhanced by the presence of two data analysts who engaged in an iterative process during data analysis, maintained ongoing communication, and reached a consensus on a shared coding framework. However, there are several important limitations to this study. We did not conduct qualitative interviews among all the desired participant groups, specifically HI committee drop-outs and trial non-participants, or in non-HI villages. Participants in these first two groups either refused consent (Not interested to participate in the interviews) or were unavailable due to relocation. This information would have been valuable in obtaining a sense of the participants’ experience in this study for the significance of having alternative viewpoints. Furthermore, it could have been beneficial to obtain qualitative information from the non-HI villages as well. This could have helped explain why some households, even without the intervention, showed significant levels of HI adoption, as indicated by the quantitative data. Additionally, the study was conducted by a team of investigators affiliated with the MMP. It could be possible that the investigators’ backgrounds may have influenced participant responses. The purposive selection of participants with sufficient knowledge of the topic may have biased the results, which may not have been representative of the population. This might have resulted in confirmation bias, where researchers could have selected participants who were more likely to confirm their preconceived notions [106]. Furthermore, by excluding certain individuals based on specific criteria, researchers may inadvertently omit important perspectives that could offer a more comprehensive understanding of the research topic. Lastly, the measure of the quality component of fidelity of HI implementation was from stakeholders’ perspectives which means the study did not consider the researcher’s perspective and direct observation.

### Recommendations

This assessment was relevant for informing policymakers working on malaria control about the essential resources (human, material, and financial) required for the successful implementation of HI or other community-based interventions. It also highlighted the obstacles and challenges that need to be considered when executing the programme as planned in order to achieve the most significant possible impact. Understanding these implementation challenges and outcomes is crucial for anticipating the effectiveness and sustainability of the intervention [25, 33, 64]. These results further illustrate the importance of performing fidelity assessments during pilot studies as a crucial component of the evaluation process to assess the effectiveness of an intervention, improve it, and transfer it with the best possible evidence to other contexts. With significant projected population increases in Sub-Saharan Africa, mosquitoes’ behavioural adaptation to the current control strategies, and the already documented emergence of resistance to pyrethroid insecticides, national malaria control programme managers should consider HI as a complementary measure towards the elimination of malaria in Malawi by 2030. Malawi has a range of well-established policies aimed at addressing the malaria burden. These policies include the malaria communication strategy and the community-based primary care policies [107, 108], which seek to strengthen and provide guidance for efforts aimed at both preventing malaria and addressing other health needs at the community level. Policymakers should build upon existing policies by ensuring that these policies are community-centred, and future interventions should be framed in ways that fundamentally empower communities. Future and further research should consider evaluating community-based HI’s potential to be replicated and scaled up in other settings and adopting a multidisciplinary approach to assess each location’s unique contextual factors.

## Conclusion

This is the first study in Malawi to evaluate the fidelity of implementing HI for malaria control. Community-led HI is an essential intervention for addressing the malaria burden and improving behaviour change in rural settings. The study showed that the community-led HI implementation was well executed and adopted in the rural area of Chikwawa in Malawi. There was good adherence to the intervention; however, there was a decline in implementation fidelity over time. Using trained volunteers and the local community stakeholders facilitated the fidelity and feasibility of implementing the community-led HI strategy. Being residents of the same community, it was easier to foster trust among these groups, thereby contributing to the successful implementation. However, contextual challenges such as adverse weather conditions, high cost, material unavailability and inaccessibility, insufficient capacity building, and the volunteers’ inadequate living conditions could negatively influence the implementation and success of the HI strategy. Active community participation, IEC, and intervention adaptation are vital components for achieving sustainability. Additionally, the presence of effective leadership and robust local governance structures play a significant role in ensuring long-term viability, particularly in rural and marginalised communities.

### Electronic supplementary material

Below is the link to the electronic supplementary material.

Supplementary Material 1

Supplementary Material 2

Supplementary Material 3

Supplementary Material 4

Supplementary Material 5

Supplementary Material 6

Supplementary Material 7

Supplementary Material 8

Supplementary Material 9

## Data Availability

The datasets used and analysed during the current study are available from the corresponding author upon reasonable request.

## Footnotes

1 A community epicentre is a building constructed by the Hunger Project (THP) and managed by the local communities. It houses a health clinic, microcredit bank, food bank, lecture room, library and sanitary facilities.

## Abbreviations

DDT: Dichlorodiphenyltrichloroethane
FGD: Focus Group Discussion
HA: Health Animator
HI: House Improvement
HSA: Health Surveillance Assistant
IDI: In-Depth Interview
IEC: Information Education Communication
IRS: Indoor Residual Spraying
ITN: Insecticide-Treated Net
KII: Key-Informant Interview
LMIC: Low and Middle-Income Countries
LSM: Larval Source Management
MMP: Majete Malaria Project
MWR: Majete Wildlife Reserve
NMCP: National Malaria Control Programme
THP: The Hunger Project
WHO: World Health Organization
