# Supplementary material for: Assessing the implementation fidelity, feasibility, and sustainability of community-based house improvement for malaria control in southern Malawi: a mixed-methods study
Source: BMC Public Health. 2024 Apr 2;24:951. doi: 10.1186/s12889-024-18401-4 (PMC10988826; doi:10.1186/s12889-024-18401-4)

**Assessing the implementation fidelity, feasibility, and sustainability of community-based house improvement for malaria control in southern Malawi: a mixed-methods study**

**Supplementary file 1: HI House and HI implementation (describes the process of HI implementation and provides an illustration of a typical rural house where HI was carried out).)**

The aim of HI is to modify houses to eliminate any openings where mosquitoes could enter the house. The diagrams below illustrate how this process was conducted.

**Figure S1: HI house containing window screening and closing of eaves**


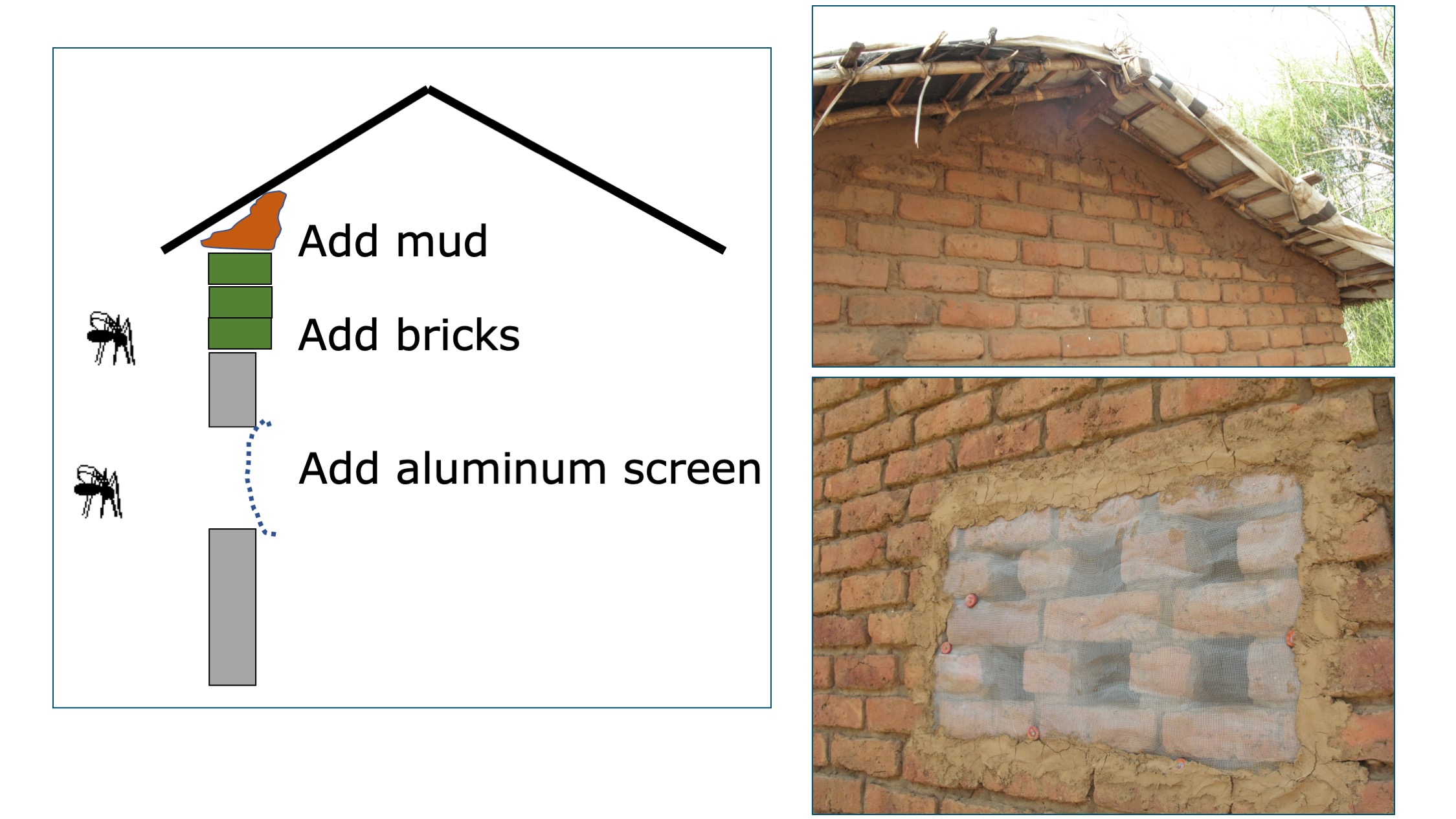


**Figure S2: Members of the HI committee conducting HI implementation**


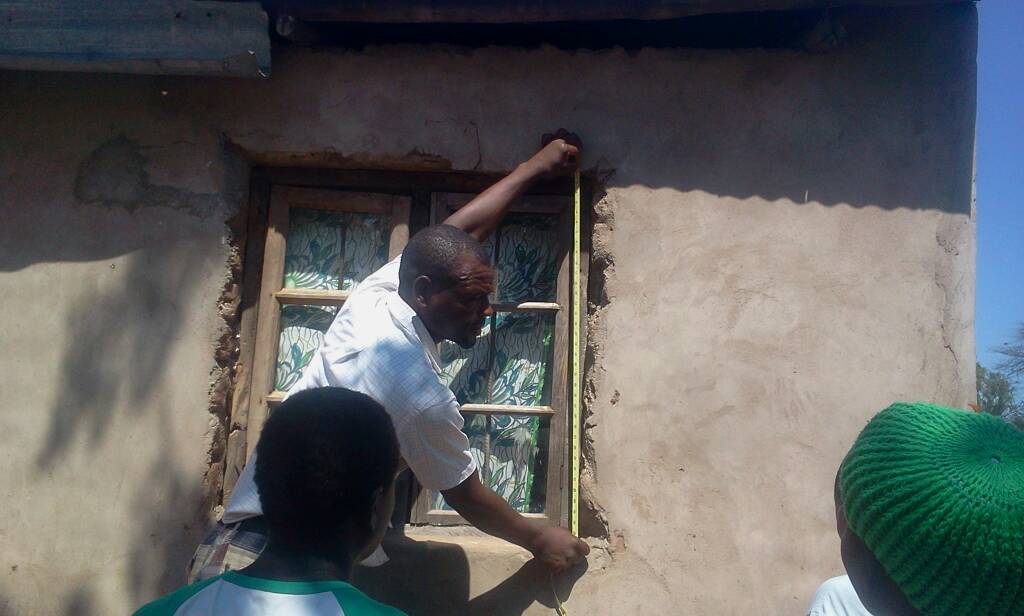

Supplement: Supplementary file 1 — Supplementary Material 1 [file 12889_2024_18401_MOESM1_ESM.docx]
