# Supplementary material for: Assessing the implementation fidelity, feasibility, and sustainability of community-based house improvement for malaria control in southern Malawi: a mixed-methods study
Source: BMC Public Health. 2024 Apr 2;24:951. doi: 10.1186/s12889-024-18401-4 (PMC10988826; doi:10.1186/s12889-024-18401-4)

**Assessing the implementation fidelity, feasibility, and sustainability of community-based house improvement for malaria control in southern Malawi: a mixed-methods study**

**Supplementary File 2: A typical rural non-HI house**

**Figure S3: non-HI house showing open eaves, unscrewed vents and windows**


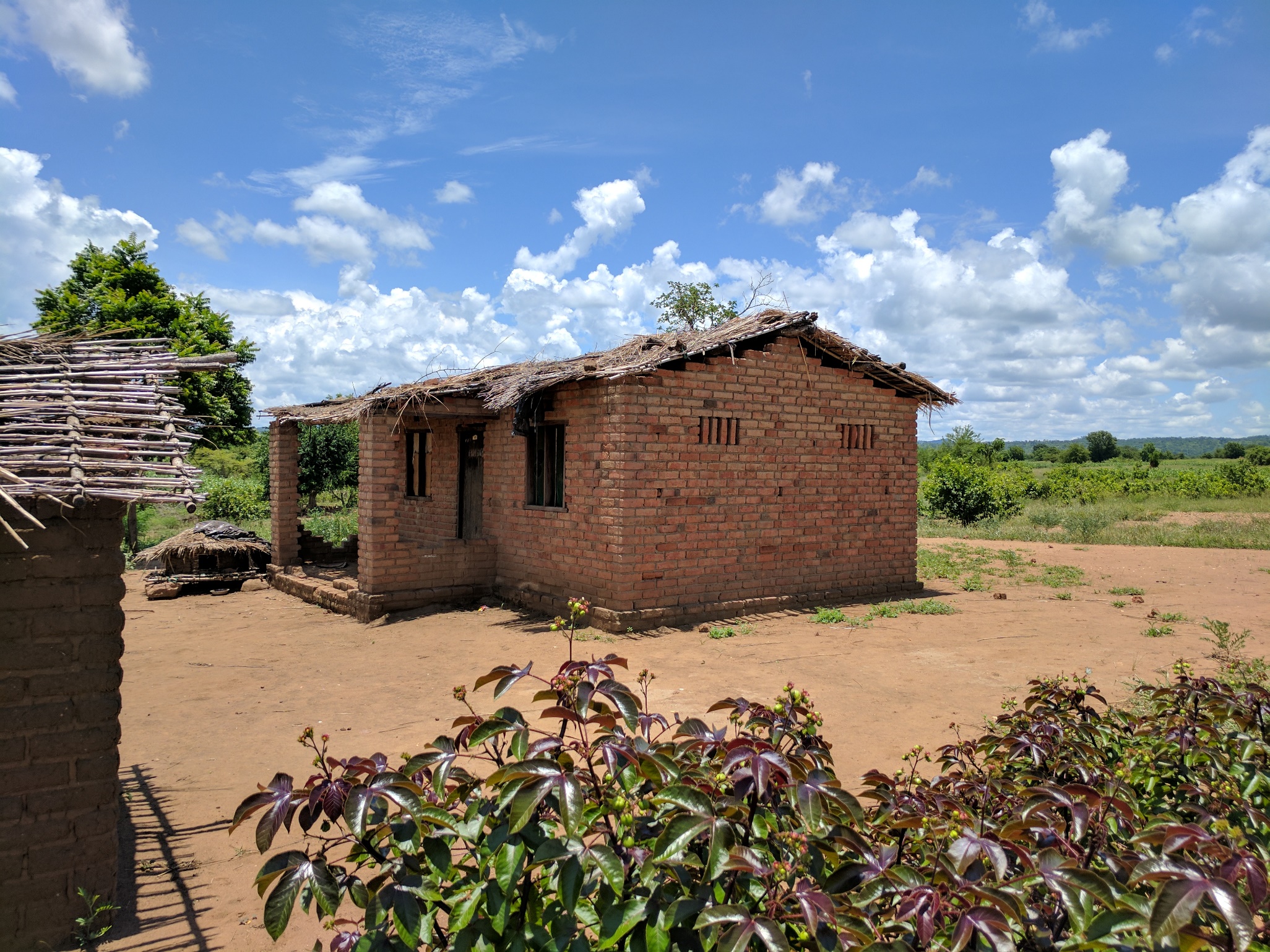


**Figure S4: non-HI house at close range with open eaves and unscreened vents**


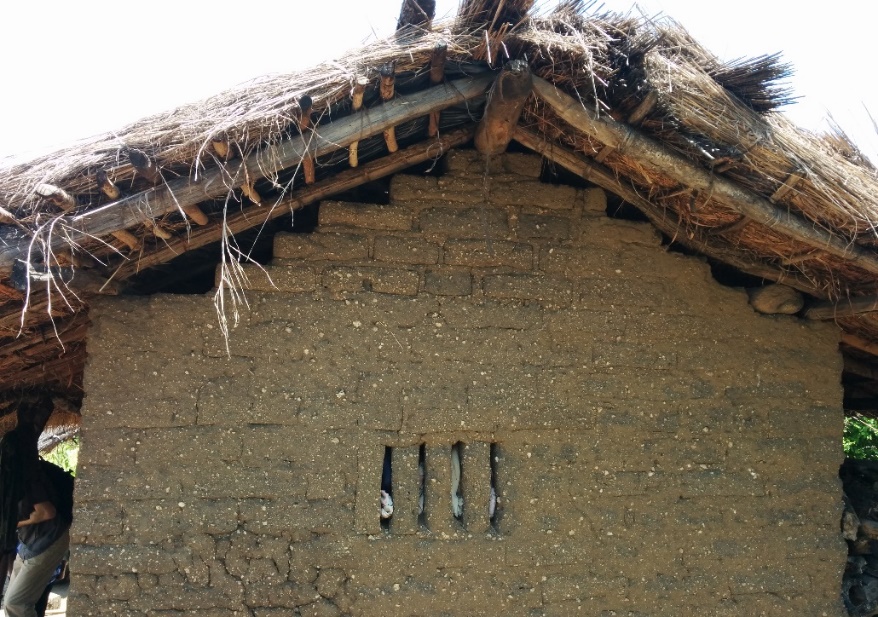

Supplement: Supplementary file 2 — Supplementary Material 2 [file 12889_2024_18401_MOESM2_ESM.docx]
