# Supplementary material for: Assessing the implementation fidelity, feasibility, and sustainability of community-based house improvement for malaria control in southern Malawi: a mixed-methods study
Source: BMC Public Health. 2024 Apr 2;24:951. doi: 10.1186/s12889-024-18401-4 (PMC10988826; doi:10.1186/s12889-024-18401-4)
Supplement: Supplementary file 3 — Supplementary Material 3 [file 12889_2024_18401_MOESM3_ESM.docx]

**Assessing the implementation fidelity, feasibility and sustainability of community-based house improvement for malaria control in southern Malawi: a mixed-methods study**

**Supplementary File 3: Focus group discussion and In-depth interview guides**

**Guide 1A: Focus Group Discussion with Health Animators**

**Qualitative Phase**

1. **Focus Group Discussion**

**Topic Guide – Health Animators – English**

1. First, I would like everyone to introduce themselves.
2. I would like you to have a few minutes to think about your knowledge, experience and perceptions of House Improvement as a malaria control intervention, and finally your participation in the ongoing HI activities in your village. Your participation is of paramount importance to the study. Is everyone ready to share their experience?

**Warm-up: Roles of Health Animators**

1. How would you describe your experience working as a health animator in your village?
2. What is your impression of house improvement as a method for preventing malaria?
3. What do you understand your role to be?
4. Which rewards/benefits did you experience by being a Health animator?
5. What challenges did you encounter during your exercise as Health animators?

[**Probe**: May you please describe each challenge in detail]

1. How did you deal with each challenge?
2. How would you describe your moments of being an animator?

[**Probe**: What are the motivating factors of working as a health animator?]

[**Probe**: What are the demotivating factors of working as a health animator?]

**To determine the feasibility (practicality) of implementing house improvement as a malaria prevention intervention**

1. How can you explain the workload/activities involved in the implementation of HI?

[**Probe**: Are the activities involving? If yes, what factors make the activities demanding? How would you suggest the activities to be approached?]

1. What resources are needed for house improvement and how available are they? [E.g., Wire gauze, bricks and mud, timber, nails, amount of time devoted to implementing HI]

[**Probe**: Anything else?]

[**Probe**: If not, what potential solutions are there to making them available?]

[**Probe**: Do you have time for obtaining these commodities? Do you have access to these commodities?]

[**Probe**: Do you have any alternative materials that you can think of?]

1. What duties/chores are foregone by engaging in HI activities?
2. What is it that could be required to have a modern house (Iron sheets, cement, window glasses, closed eaves) compared to having a traditional house (Grass thatched, mud and bricks) [**Probe**: (if you have a modern house): What made you decide on a modern house; (if you have a traditional house:) Why?]
3. Are people in the community willing to pay for improved quality housing for preventing malaria?
4. Do we have any reasons why some people change from having traditional houses to modern houses?

**To determine the fidelity (adherence to the standards of implementing house improvement and quality of the program delivery) of implementing house improvement as a malaria prevention intervention**

1. Is House Improvement done to standard in all the houses? (Full eave closure, windows covered in wire gauze, wooden doors) [**Probe**: describe the standard for HI]
2. Does the community stick to these standards?

[**Probe**: Do you think the standards of improving the houses can be adhered to? What would make the community follow these standards?]

1. How would you describe the quality at which HI is being implemented in your village?

**Sustainability**

1. Reflecting on the situation before and after the introduction of the intervention, what changes if, any, have you noticed in your communities with regards to the intervention?

[**Probe**: What has been the most obvious change for your community?]

1. Do you have any recommendation on how to involve your community into HI activities more effectively?

[**Probe**: What specific actions could be taken to improve community involvement?]

If you were asked to recommend this intervention to other communities, what would you say?

[**Probe**: How best could the implementation process be carried out]

1. Will you be able to continue with your duties as health animators after the end of the project?

[**Probe**: What are your plans?]

1. Do you think that the HI committees will continue maintaining house improvement activities after the end of the project?

[**Probe**: What are their plans?]

**Guide 1B: Focus Group Discussion with HI Committee members**

**Qualitative Phase**

1. **Focus Group Discussion**

**Topic Guide – HI Committee – English**

1. First, I would like everyone to introduce themselves.
2. I would like you to have a few minutes to think about your knowledge, experience and perceptions of House Improvement as a malaria control intervention, and finally your participation in the ongoing HI activities in your village. Your participation is of paramount importance to the study. Is everyone ready to share their experience?

**Warm-up: Roles of HI Committee Members**

1. How would you describe your experience working as a member of the HI committee?
2. What is your impression of house improvement as a method for preventing malaria?
3. What do you understand your role to be?
4. Which rewards/benefits did you experience by being a HI committee member?
5. What challenges did you encounter during your exercise as HI committee members? [**Probe**: May you please describe each challenge in detail]
6. How did you deal with each challenge?
7. How would you describe your moments of being on the HI committee?

[**Probe**: What are the motivating factors of working in the HI committee?]

[**Probe**: What are the demotivating factors of working in the HI committee?]

**To determine the feasibility (practicality) of implementing house improvement as a malaria prevention intervention**

1. How can you explain the workload/activities involved in the implementation of HI?

[**Probe**: If yes, what factors make the activities demanding? How would you suggest the activities to be approached?]

1. What resources are needed for house improvement and how available are they? [E.g. Wire gauze, bricks and mud, timber, nails, amount of time devoted to implementing HI]

[**Probe**: Anything else?]

[**Probe**: If not, what potential solutions are there to making them available?]

[**Probe**: Do you have time for obtaining these commodities? Do you have access to these commodities?]

[**Probe**: Do you have any alternative materials that you can think of?]

1. What duties/chores are foregone by engaging in HI activities?
2. What is it that could be required to have a modern house (Iron sheets, cement, window glasses, closed eaves) compared to having a traditional house (Grass thatched, mud and bricks) [**Probe**: (if you have a modern house): What made you decide on a modern house; (if you have a traditional house: ) Why?]
3. Are people in the community willing to pay for improved quality housing for preventing malaria?
4. Do we have any reasons why some people change from having traditional houses to modern houses?

**To determine the fidelity (adherence to the standards of implementing house improvement and quality of the program delivery) of implementing house improvement as a malaria prevention intervention**

1. Is House Improvement done to standard in all the houses? (Full eave closure, windows covered in wire gauze, wooden doors) [**Probe**: describe the standard for HI]
2. Does the community stick to these standards?

[Probe: Do you think the standards of improving the houses can be adhered to? What would make the community follow these standards?]

1. How would you describe the quality at which HI is being implemented in your village?

**Sustainability**

1. Reflecting on the situation before and after the introduction of the intervention, what changes if, any, have you noticed in your communities with regards to the intervention?

[**Probe**: What has been the most obvious change for your community?]

1. Do you have any recommendation on how to involve your community into HI activities more effectively?

[**Probe**: What specific actions could be taken to improve community involvement?]

If you were asked to recommend this intervention to other communities, what would you say?

[**Probe**: How best could the implementation process be carried out]

1. Do you think that the committees will continue maintaining house improvement activities after the end of the project?

[**Probe**: What are their plans?]

**Closing Remarks**

1. Is there anything we have not talked about that you think is important?
2. Do you have any questions or comments?

**Conclusion**

1. Thank you for participating in this discussion. Your opinions will be a valuable asset to the study.

**Guide 1C: Focus Group Discussion with community participants**

**Qualitative Phase**

1. **Focus Group Discussion**

**Topic Guide – Community Participants – English**

1. First, I would like everyone to introduce him or herself.
2. I would like you to have a few minutes to think about your knowledge, experience and perceptions of House Improvement as a malaria control intervention, and finally your participation in the ongoing HI activities in your village. Your participation is of paramount importance to the study. Is everyone ready to share their experience?

**Warm-up: Community Experiences with implementing HI**

1. How would you describe your experience in implementing HI in your village?
2. What is your impression of house improvement as a method for preventing malaria?
3. What challenges do you encounter during your exercise of implementing HI?

[**Probe**: May you please describe each challenge in detail]

1. How do you deal with each challenge?
2. Do you have any reasons why people build houses with open eaves?

**To determine the feasibility of implementing house improvement as a malaria prevention intervention**

1. How would you describe the process of installing items required for house improvement and the implementation of House improvement activities on a monthly basis (i.e., Schedule, Maintenance after rains and in general, how responsibilities are shared)?

[**Probe**: Elaborate your thoughts concerning these activities? If demanding, what factors make these activities demanding? How would you suggest the activities to be approached? Do you ask for help/assistance on HI?]

1. What resources are needed for house improvement and how readily available are they? [E.g., Wire gauze, bricks and mud, timber, nails, amount of time devoted to implementing HI]

[**Probe**: Anything else?]

[**Probe**: If not, what potential solutions are there to making them available?]

[**Probe**: Do you have time for obtaining these commodities? Do you have access to these commodities?]

[**Probe**: Do you have any alternative materials that you can think of?]

1. What duties/chores are foregone by engaging in HI activities?
2. What is it that could be required to have a modern house (Iron sheets, cement, window glasses, closed eaves) compared to having a traditional house (Grass thatched, mud and bricks) [Probe: (if you have a modern house): What made you decide on a modern house; (if you have a traditional house :) Why?]
3. Are people in the community willing to pay for improved quality housing for preventing malaria?

**To determine the fidelity (adherence to the standards of implementing house improvement and quality of the program delivery) of implementing house improvement as a malaria prevention intervention**

1. Is House Improvement done to standard in all the houses? (Full eave closure, windows, wooden doors, covered in wire gauze) [**Probe**: describe the standard for HI]
2. Does the community stick to these standards?

[**Probe**: Do you think the standards of improving the houses can be adhered to? What would make the community follow these standards?]

1. How would you describe the quality at which HI is being implemented in your village?

**Sustainability**

1. Reflecting on the situation before and after the introduction of the intervention, what changes if, any, have you noticed in your communities with regards to the intervention?

[**Probe**: What has been the most obvious change for your community? For example….?]

1. Do you have any recommendations on how to involve your community into HI activities more effectively?

[**Probe**: What specific actions could be taken to improve community involvement?]

1. If you were asked to recommend this intervention to other communities, what would you do?

[**Probe**: How best could the implementation process be carried out]

**Closing Remarks**

1. Is there anything we have not talked about that you think is important?
2. Do you have any questions or comments?

**Guide 1D: Topic Guide (IDI) Community Participants (Male and Female) – English**

**Qualitative Phase**

1. **In Depth Interviews**

**Topic Guide – IDI – English**

**Introductory Remarks**

1. First, I would like you to introduce yourself.
2. I would like you to have a few minutes to think about your knowledge, experience and perceptions of House Improvement as a malaria control intervention, and finally your participation in the ongoing HI activities in your village. Your participation is of paramount importance to the study. Are you ready to share your experience?

**Warm-up: Understanding your role and general principles of malaria**

1. Is malaria considered a serious health problem in this community?

[**Probe**: Why? Why not?]

1. What kind of things do people in this community usually do to protect themselves from malaria?
2. Do you know that your village is participating in malaria control through HI?
3. What is your impression of house improvement as a method for preventing malaria?
4. How would you describe your experience in implementing HI in your village?
5. In what ways have you engaged with the intervention?

[**Probe**: What are the motivating factors for participating in these activities?

What are the demotivating factors from participating in HI activities?]

1. What challenges do you encounter during your exercise of implementing HI?

[**Probe**: May you please describe each challenge in detail]

1. How do you deal with each challenge?
2. Do you have any reasons why people build houses with open eaves? Do eaves have any intended use?

**To determine the feasibility of implementing house improvement as a malaria prevention intervention**

1. How can you explain the workload/activities involved in the implementation of HI?

[**Probe**: Are the activities involving? If yes, what factors make the activities demanding? How would you suggest the activities to be approached?]

1. What resources are needed for house improvement and how available are they? [E.g., Wire gauze, bricks and mud, timber, nails, amount of time devoted to implementing HI]

[**Probe**: Anything else?]

[**Probe**: If not, what potential solutions are there to making them available?]

[**Probe**: Do you have time for obtaining these commodities?

[**Probe**: Do you have any alternative materials that you can think of?

1. What duties/chores are foregone by engaging in HI activities?
2. What is it that could be required to have a modern house (Iron sheets, cement, window glasses, closed eaves) compared to having a traditional house (Grass thatched, mud and bricks)?
3. Are people in the community willing to pay for improved quality housing for preventing malaria?
4. Do we have any reasons why some people change from having traditional houses to modern houses?

**To determine the fidelity (adherence to the standards of implementing house improvement and quality of the program delivery) of implementing house improvement as a malaria prevention intervention**

1. Is House Improvement done to standard in all the houses? (Full eave closure, windows covered in wire gauze) [**Probe**: describe the standard for HI]
2. Does the community stick to these standards?

[**Probe**: Do you think the standards of improving the houses can be adhered to? What would make the community follow these standards?]

1. How would you describe the quality at which HI is being implemented in your village?

**Closing Remarks:**

1. Is there anything we have not talked about that you think is important for me to know?
2. Do you have any questions or comments for me?

**Conclusion**

1. Thank you for participating in this discussion. Your opinions will be a valuable asset to the study

**Guide 1E: Topic Guide (KII) Key Informant Interviews (HSAs, Chiefs, from HI village) – English**

**Qualitative Phase**

1. **Key Informant Interviews**

**Topic Guide – KII – English**

**Introductory Remarks**

1. First, I would like you to introduce yourself.
2. I would like you to have a few minutes to think about your knowledge, experience and perceptions of House Improvement as a malaria control intervention, and finally your participation in the on-going HI activities in your village. Your participation is of paramount importance to the study. Are you ready to share your experience?

**Warm up: Understanding your role and general principles of malaria**

1. Could you please describe what are considered serious health problems in this community?
2. Is malaria considered a serious health problem in this community?

[Probe: Why? Why not?]

1. What kind of things do people in this community usually do to protect themselves from malaria?
2. How do you understand your role of promoting health in this community to be?
3. Can you please elaborate how critical is your influence towards promoting health in this community?

**To determine the feasibility of implementing house improvement as a malaria prevention intervention**

1. How can you explain the workload/activities involved in the implementation of HI?

**[Probe**: If yes, what factors make the activities demanding? How would you suggest the activities to be approached?]

1. What resources are needed for house improvement and how available are they? [E.g., Wire gauze, bricks and mud, timber, nails, amount of time devoted to implementing HI]

[**Probe**: Anything else?]

[**Probe**: If not, what potential solutions are there to making them available?]

[**Probe**: Do you have time for obtaining these commodities? Do you have access to these commodities?]

[**Probe**: Do you have any alternative materials that you can think of?]

1. What duties/chores are foregone by engaging in HI activities?
2. What is it that could be required to have a modern house (Iron sheets, cement, window glasses, closed eaves) compared to having a traditional house (Grass thatched, mud and bricks)
3. Are people in the community willing to pay for improved quality housing for preventing malaria?

**To determine the fidelity (adherence to the standards of implementing house improvement and quality of the program delivery) of implementing house improvement as a malaria prevention intervention**

1. Is House Improvement done to standard in all the houses? (Full eave closure, windows covered in wire gauze) [**Probe**: describe the standard for HI]
2. Does the community stick to these standards?

[**Probe**: Do you think the standards of improving the houses can be adhered to? What would make the community follow these standards?]

1. How would you describe the quality at which HI is being implemented in your village?

**Closing Remarks:**

1. Is there anything we have not talked about that you think is important for me to know?
2. Do you have any questions or comments for me?

**Conclusion**

1. Thank you for participating in this discussion. Your opinions will be a valuable asset to the study

**Guide 1F: In-Depth Interview with HI Committee Dropout members**

**Qualitative Phase**

1. **In-Depth Interview**

**Topic Guide – IDI HI Committee Dropouts – English**

1. First, I would like everyone to introduce themselves.
2. I would like you to have a few minutes to think about your knowledge, experience and perceptions of House Improvement as a malaria control intervention, and finally your views on the ongoing HI activities in your village. Your participation is of paramount importance to the study. Are you ready to share your experience?

**Warm-up: Experience of your role as former HI Committee Members**

1. How would you describe your previous experience working as a member of the HI committee?
2. What did you understand your role to be? What challenges did you encounter when you participated in house improvement activities as an HI committee member?

[**Probe**: May you please describe each challenge in detail]

1. How did you deal with each challenge?
2. Could you please explain why you decided to leave the HI committee?

[**Probe**: What were the demotivating factors of being on the committee?]

**To determine the feasibility (practicality) of implementing house improvement as a malaria prevention intervention**

1. How can you explain the workload/activities involved in the implementation of HI?

[**Probe**: Are the activities involving? If yes, what factors make the activities demanding? How would you suggest the activities to be approached?]

1. What resources are needed for house improvement and how available are they? [E.g. Wire gauze, bricks and mud, timber, nails, amount of time devoted to implementing HI]

[**Probe**: Anything else?]

[**Probe**: If not, what potential solutions are there to making them available?]

[**Probe**: Do you have time for obtaining these commodities? Do you have access to these commodities?]

[**Probe**: Do you have any alternative materials that you can think of?]

1. What duties/chores are foregone by engaging in HI activities?
2. What is it that could be required to have a modern house (Iron sheets, cement, window glasses, closed eaves) compared to having a traditional house (Grass thatched, mud and bricks) [**Probe**: (if you have a modern house): What made you decide on a modern house; (if you have a traditional house:) Why?]
3. Are people in the community willing to pay for improved quality housing for preventing malaria?
4. Do we have any reasons why some people change from having traditional houses to modern houses?

**Closing Remarks**

1. Is there anything we have not talked about that you think is important?
2. Do you have any questions or comments?

**Conclusion**

1. Thank you for participating in this discussion. Your opinions will be a valuable asset to the study.

**Guide 1G: Topic Guide (IDI) Community Non-Participants (Male and Female)**

**Qualitative Phase**

1. **In-Depth Interviews**

**Topic Guide – IDI – English**

1. First, I would like everyone to introduce themselves.
2. I would like you to have a few minutes to think about your knowledge, experience and perceptions of House Improvement as a malaria control intervention. Your participation is of paramount importance to the study. Is everyone ready to share their experience?

**Warm-up: Roles of the Community on HI**

1. Is malaria considered a serious health problem in this community?

[**Probe**: Why? Why not?]

1. What kind of things do people in this community usually do to protect themselves from malaria?
2. What is your impression of house improvement as a method for preventing malaria?

[**Probe**: What does house improvement involve?]

1. Do you have any reasons why people build houses with open eaves? Do eaves have any intended use?
2. Why did you decide not to participate in the ongoing HI trial?

[**Probe**: What were the demotivating factors of participating in the trial?]

**To determine the feasibility of implementing house improvement as a malaria prevention intervention**

1. How can you explain the workload/activities involved in the implementation of HI?

[**Probe**: Are they too involving? If yes, what factors make the activities demanding? How would you suggest the activities to be approached?]

1. What resources are needed for house improvement and how available are they? [E.g., Wire gauze, bricks and mud, timber, nails, amount of time devoted to implementing HI]

[**Probe**: Anything else?]

[**Probe**: If not, what potential solutions are there to making them available?]

1. Are you able to get these commodities?

[Probe: Are these commodities readily available? Do you have time for obtaining these commodities?

**Closing Remarks**

1. Is there anything we have not talked about that you think is important?
2. Do you have any questions or comments?
