# Supplementary material for: Assessing the implementation fidelity, feasibility, and sustainability of community-based house improvement for malaria control in southern Malawi: a mixed-methods study
Source: BMC Public Health. 2024 Apr 2;24:951. doi: 10.1186/s12889-024-18401-4 (PMC10988826; doi:10.1186/s12889-024-18401-4)
Supplement: Supplementary file 6 — Supplementary Material 6 [file 12889_2024_18401_MOESM6_ESM.docx]

**Assessing the implementation fidelity, feasibility and sustainability of community-based house improvement for malaria control in southern Malawi: a mixed-methods study**

**Supplementary File 6: Codebook**

The description below shows the process of deriving codes and coming up with the codebook.

**Description**:

Multiple codes can be used to code the same statement from participants. The process of developing the codebook was conducted to avoid forgetting the codes.

**Key**:

- Besides applying multiple codes, we should capture sufficient background information from the interviewer’s question.
  - If a participant’s response will be difficult to understand without the question, code the question with the response.
  - A quotation length should be long enough for readers to understand its meaning without context.
- Be careful that the right code is being applied to the right passage.
- Passages with participant responses should not go uncoded.

**Table S6: Shows codes derived along with the definitions**

| **NO** | **CODE** | **DEFINITION** | **INCLUSION** | **EXCLUSION** |
| --- | --- | --- | --- | --- |
|  | **HI Feasibility** | **Parent code:** Explains the views and perceptions of the participants regarding the feasibility of HI's implementation as a malaria preventive intervention. In other words, if a passage has a child code, the parent code must also be applied. ONLY the parent code is applicable IF none of the child codes does. | All participants’ views in regard to the feasibility of HI being implemented in their areas. | Participants' opinions on additional malaria prevention measures implemented in their area. |
|  | **HI Feasibility:** Installation process | Describes participant’s views and perception on the process of installing items required for house improvement and the implementation of HI activities on a monthly basis |  |  |
|  | **HI Feasibility:** Resources needed | Describes participants’ views on the resources required for HI and how readily available are the resources. |  |  |
|  | **HI Feasibility:** Duties forgone | Describes participants’ views and opinions on the duties/chores that are forgone by engaging in HI |  |  |
|  | **HI Feasibility:** Requirement for modern house | Describes participants’ views on what is required to have a modern house in their community and why people would want to change from a traditional house to a modern house |  |  |
|  | **HI Feasibility:** Willingness to pay | Describes participants’ views and opinions on people’s willingness to pay for improved houses for malaria prevention in their community. |  |  |
|  | **HI Fidelity** | **Parent code:** Describes the participants' views and opinions on program quality, program delivery standards, and adherence to HI implementation standards. In other words, if a passage has a child code, the parent code must also be used. ONLY the parent code is applicable IF none of the child codes fit. | All participants’ views in regard to the fidelity of HI being implemented in their areas. | Participants’ views on other interventions being implemented in their area for malaria prevention. |
|  | **HI Fidelity:** Adherence to standards | Describes participants’ views and opinions if the HI was done according to the set standards and if it is done in all houses in their community. |  |  |
|  | **HI Fidelity** Quality of the implementation | Describes participants’ views on the quality at which HI is being implemented in their community. |  |  |
|  | **HI Sustainability** | **Parent code:** Explains the participants' perspectives on the changes observed in their communities as a result of the HI and their recommendations for how to better involve their communities in HI activities. This means that if a passage is coded with a child code, this parent code must also be applied. IF none of the child codes fits then ONLY the parent code applies. | All participants’ views in regard to the sustainability of HI being implemented in their areas. | Participants’ views on other interventions being implemented in their area for malaria prevention |
|  | **HI Sustainability:** Noticeable changes | Describes participant’s views and opinions on the changes observed due to HI intervention in their communities |  |  |
|  | **HI Sustainability**: Intervention Continuity | Describes health animators’ views on the continuity of their duties at the end of the project and their opinion if the HI committees will continue maintaining house improvement activities after the end of the project. |  |  |
|  | **HI Sustainability:** Recommendations | Describes participants’ recommendations on how to involve their community in HI activities more effectively and how they can recommend the intervention to other communities. |  |  |
