# Supplementary material for: Assessing the implementation fidelity, feasibility, and sustainability of community-based house improvement for malaria control in southern Malawi: a mixed-methods study
Source: BMC Public Health. 2024 Apr 2;24:951. doi: 10.1186/s12889-024-18401-4 (PMC10988826; doi:10.1186/s12889-024-18401-4)
Supplement: Supplementary file 7 — Supplementary Material 7 [file 12889_2024_18401_MOESM7_ESM.docx]

**Assessing the implementation fidelity, feasibility and sustainability of community-based house improvement for malaria control in southern Malawi: a mixed-methods study**

**Supplementary File 7: Inductive Coding Process**

**Abbreviations**

HA – Health Animator

HSA – Health Surveillance Assistant

FA-A – Focal Area A

FA-B – Focal Area B

FA-C – Focal Area C

FGD – Focus Group Discussion

IDI – In-depth Interview

KII – Key-informant Interview

1. **To determine the feasibility (practicality) of implementing house improvement as a malaria prevention intervention**

**Table S7: Inductive Coding Process showing theme, codes and quotes for Focus Group Discussions (FGDs), In-depth Interviews (IDIs) and Key-informant Interviews (KII)**

Theme: Workload involved in HI

| Codes | Quotes | Theme |
| --- | --- | --- |
| Heavy/Hard | “Previously, we thought the job is too much but when we started doing it, we realized that the work is simple. We accept to do the work and it became simple.” (P8 HA FA-A) |  |
| Heavy/Hard | “Um, initially, it was very tiring because most people had not realized yet what the benefit of closing open eaves was. But today people realize that house improvement is one way by which malaria can be reduced in our village or at the household level.” (P4 HA FA-B) |  |
| Heavy/Hard | “It was tiring because the time we went to give out gauze wire, we would give the community residents three days to close the windows with the gauze wire, then we would go back to check the work. When we followed up with their work, we would find that they did not screen their windows with the gauze wire. If we asked them why they had not put gauze wire on the windows, which was intended for malaria reduction in the village, they would say, “I can’t do this, is this not for drawing blood from us?” So, it would take a lot of time to convince them that “it’s not that, the reason for this is that they should have a healthy life, and if we have a healthy life, then the money that we used to spend on attending the hospital or buying drugs will be used for other things in the household.” So, we would go and talk to committee members to calm them down, so that they may continue working. So, for an animator, it was too much, settling disputes in the village with the help of the chief.” (P5 HA FA-B) |  |
| Manageable | “The house improvement work is not difficult depending on how the house was built. First, we check from the outside if the closure of open eaves is complete. Then we go inside to see if there are spaces/holes and close them if there are any.” (P5 HA FA-C) |  |
| Manageable | “This work is not tiring. Why? Because we support each other. An animator supports committee members. Committee members support villagers. If there are challenges, chiefs also get involved, ensuring everything goes well. So, because of the different roles that different groups have, the work is easier to do. (P3 HA FA-C) |  |
| Manageable | “Conquering with P3, um, this work is not difficult because everyone plays a part. An animator checks on committee members. Committee members check on how villagers are improving their houses. Therefore, it’s not tiring. There are houses that already had open eaves closed before the project came. So, when the project started, they just needed mud to plaster the walls, and it was only a matter of 2 days to complete plastering the house.” (P6 HA FA-C) |  |
|  |  |  |

Theme: Solutions to HI Workload

| Codes | Quotes | Theme |
| --- | --- | --- |
| Training the community | “When we train people, it has been a simple method because everyone is taking part and is working on their own. Once we tell anyone that we are coming tomorrow with the gauze wire, you should close the open eaves, in the next morning, we find them that they have done the work.” (P8 HA FA-A) |  |
| Sensitization | “With the sensitization meetings that we were conducting in the communities, the work started becoming simple because everyone understood that once they have done the job, they will protect his/her life and the life of their children.” (P5 HA FA-A) |  |
| Volunteering to help others | “if we gave them gauze wire and told them what to do, like closing open eaves and sealing openings, we would tell them that we would come back on such and such a date. If we came back and found that they did not do what we told them to do, with the help of committee members, we would look for broken bricks, fetch water for making mud, and then use the mud to seal the wall, helping them to close open eaves and other openings.” (P7 HA FA-B) |  |
| Collaboration amongst community members | “What could be done is that committee members and village chiefs should hold hands to make the work doable. Because if you are doing the work by yourself without involving others, then the work will be harder for you.” (P5 HA FA-C) |  |
| Collaboration amongst community members | “What we could do to make this work not difficult is that we need to work together, everyone must take part. Both a committee member and an animator should take part. Then the work won’t be difficult. If there are weaknesses, we need to find out what led to those weaknesses, and address the challenge that is there. And if we address the challenge, then everything will go smoothly.” (P2 HA FA-B) |  |

Theme: Resources required for HI

| Codes | Quotes | Theme |
| --- | --- | --- |
| Wire gauze, Bricks, Mud, Nails, Hammer, stones, timber | “Materials? You need to look for bricks or broken bricks for closing open eaves. If you receive gauze wire, or if you buy gauze wire yourself when the project phases out, you need to look for nails, a hammer, or a good stone for a hammer, and pieces of timber for sticking the edges of the wire to the wall, then plastering the edges of the window so that the gauze wire does not come out.” (P6 HA FA-B) |  |
| Wire gauze, Bricks, Mud, Nails, reeds | “One necessary step when improving the house is the house owner looking for bricks. When bricks have been organized, then you start closing open eaves, making sure the walls are in contact with the roof. When closing open eaves, you will need mud. For the windows, you will need nails for nailing the gauze wire. You will also need some baboons or reeds for sticking the gauze wire to the window flame so that it doesn’t come off when there’s a strong wind.” (P1 HA FA-C) |  |
| Scissors, Hammer | “You need nails, scissors, a hummer, so that the work should be simple.” (P1 HA FA-A) |  |
| Bricks, Water, Knife, gauze wire, scissors, tape measure, hammer | “This work, we need all necessary materials for this work to be simple. We need bricks, water, a building knife, gauze wire, scissors, tape measure and the hummer so that the builder should get it easy.” (P3 HA FA-A) |  |
|  |  |  |

Theme: Availability of materials

| Codes | Quotes | Theme |
| --- | --- | --- |
| Not all readily available | “These materials are not readily available. Like bricks, it was difficult to find them during the rainy period. Nails are particularly hard to find because we have to buy them. So, it is difficult to find the materials at the right time.” (P4 HA FA-B) |  |
| Not all readily available | “Um, these tools are not readily available. Why? Because the gauze wire has to come from a town with our project officers. A hoe or a panga knife or building knife is readily available because people have them in their homes, and so is the tape or the hammer because committee members keep them. Delays in receiving the gauze wire sometimes delay our work.” (P2 HA FA-C) |  |
| Not all readily available | “If one material is missing and there is a gauze wire, we need to get a building knife, level and the bricks. Once we put the gauze wire, we have done the work and when we are going to do the work, we have materials like level, building knife and the like, if we do not have a gauze wire, we report to the office.” (P3 HA FA-A) |  |
| Not all readily available | “They are not readily available because if we visit someone and find that their house does not have eaves closed, then there are a number of things that are required at that time. To have the open eaves closed means they have to look for bricks. The same goes for nails. In short, they are not found at once – it takes time to gather them.” (P5 HA FA-B) |  |
|  |  |  |

Theme: Solutions to the availability of materials

| Codes | Quotes | Theme |
| --- | --- | --- |
| Timely provision of resources at the local offices | “To ensure these things are readily available, we have offices at the Epicentre here as well as at Chapananga. If these offices could be supplied with the materials, it would be easier for us to come and collect them from here and distribute them to people.” (P2 HA FA-C) |  |
| Timely provision of resources at the local offices | “Some of the materials like a hammer, scissors, tape measure plus gauze wire were being supplied by the office, Majete malaria project.” (P6 HA FA-B) |  |
| Improvising materials | “Just to add on that, it can happen that the nails are missing, you need to get piece work and get money to buy nails and, in the process, malaria is getting worse. We then take bamboo and shape them like nails, then we use them when putting the gauze wire in the windows” (P2 HA FA-A) |  |
| Improvising materials | “If the nails are missing, for us to do a long-lasting thing, we cut bicycle wires and use it, bamboo cannot work on a window frame.” (P7 HA FA-A) |  |
| Sending reports in time | “What’s required is to report immediately if the gauze wire stock is finishing. That would ensure ready availability of the wire because when one supply is about to finish another arrives.” (P3 HA FA-C) |  |
| Purchasing | “We find materials for house improvement through various ways. Some of the materials we buy them. Nails for nailing gauze wire for example, we have to buy them.” (P2 HA FA-B) |  |
| Purchasing | “For nails, some get them from well-wishers, but not many give them for free, you have to pay.” (P7 HA FA-B) |  |
| Doing work in phases | “Responding to the first and second questions, I think this work is done in phases. We can’t supply gauze wire when open eaves have not been closed. Every part of the work has its own phase. If we are going to mould bricks, it means what will be supplied are materials for brick making. Similarly, if we are going to close open eaves, what will be supplied are materials for that work, and so if you are going to put gauze wire on the windows.” (P6 HA FA-B) |  |

Theme: Gauze wire purchase

| Codes | Quotes | Theme |
| --- | --- | --- |
| Expensive | “We can’t buy gauze wire on our own because we can’t raise money.” (P4 HA FA-C) |  |
| Expensive | “It’s hard for someone to buy gauze wire with their own money because the wire costs a lot of money. If someone fails to buy a nail that costs 50 or 100 Kwacha, they can’t buy gauze wire given how expensive it is.” (P7 HA FA-B) |  |
| Expensive | “I think there is no one in our community who can afford to buy the gauze wire because the money that we get in the villages is too little and the gauze wire is expensive.” (P1 HA FA-A) |  |
| Few can afford | “I think some can afford and some cannot afford because we get money differently, others get money easily and yet don’t get money easily. We cannot promise that people in our community can buy gauze wire easily.” (P8 HA FA-A) |  |
| Affordable | “If the project stopped providing free gauze wire, we can buy it as long as it’s available in our village stores and at a lower price because having it on the windows is protecting our lives from illness.” (P1 HA FA-C) |  |
| Affordable | “I have been talking to some people and they have been telling me that “We are ready [to buy on our own] even if the project stops providing gauze wire.” “Why?” “Because the money I spend on managing malaria-caused illness annually is more than I can spend on buying gauze wire, and the money that I save by preventing malaria illness I can use to buy a goat.” So, people are ready to buy because they realize the benefit of the gauze wire. And I know someone who went and bought gauze wire on their own before the project started providing it. So, there’s proof that people can buy it.” (P3 HA FA-C) |  |
| Purchasing in a group | “If the gauze wire was cheap, many people would be able to buy it. But since it’s expensive, maybe if people contribute money and buy as a group. But everyone on their own, especially if they have no means of support, can’t buy it.” (P5 HA FA-C) |  |
|  |  |  |

Theme: HI Burden on daily life

| Codes | Quotes | Theme |
| --- | --- | --- |
| No burden | “No, it doesn’t stop us. Why? Because we divide the work between days – we might Wednesday of this week as a day for distributing materials, then we might have Wednesday of the following week as a day for checking if people closed the windows with the gauze wire that was supplied. And we don’t go there in the morning, we go there around 2 o’clock, meaning you have been able to do all your chores in the morning. So, it doesn’t stop us.” (P6 HA FA-B) |  |
| No burden | “For us, it does make us not to do other work because we have a time table. We can do our work in the morning and around 2, we can do the house improving work.” (P7 HA FA-A) |  |
| No burden | “The house improvement work does not stop us from completing our household chores. All the house improvement activities are planned in advance, so we know in advance what we are going to be doing on such and such a date or in such and such a month. If you have a calendar of events, it’s easier to accomplish your daily activities.” (P1 HA FA-C) |  |
| No burden | “This work does not interrupt our household activities. Why? Because we have work plans. If we agree to meet on Wednesdays, then I ensure that all my household chores are completed before that day. And if we meet just once a week, it means I have more days for doing household chores.” (P3 HA FA-C) |  |
| Proves a burden sometimes | “Sometimes it does stop us from doing our routine activities. This happens for a number of reasons. You might plan to go out and distribute gauze wire, but on that same day you might receive an unexpected message – like the way it happened today, we had planned to conduct this research at Majete but because we had to be around to receive materials, the program sort of changed. So sometimes it interferes with other programs.” (P2 HA FA-B) |  |

Theme: Community willingness to pay for modern housing

| Codes | Quotes | Theme |
| --- | --- | --- |
| Depends on income | “Some can manage and some cannot because we have different financial statuses and because of that, the only person who can afford is the one who has money. As for me, I can manage because I have seen the advantage of this.” (P7 HA FA-A) |  |
| Depends on income | “Some elderly people cannot manage and they need someone to come and help them. For others, like those who do businesses, they can afford because they get money often.” (P5 HA FA-A) |  |
| Willing | “People would be willing. Why? Because of the benefits they are seeing. Malaria is a very serious condition; people would be willing to pay so that they are protected from malaria.” (P3 HA FA-C) |  |
|  |  |  |

1. **To determine the fidelity (adherence to the standards of implementing house improvement and quality of the program delivery) of implementing house improvement as a malaria prevention intervention**

Theme: HI done to Standard

| Codes | Quotes | Theme |
| --- | --- | --- |
| Not to the recommended standard | “No, some are properly closed, others are not properly closed for some reasons. There are houses, especially those with corrugated iron sheets when closed, they are fine unless something terrible happens like being blown off by the wind. But grass-thatched houses tend to have open spaces and need to be maintained frequently, and they are kinds of houses that give animators too much work, having to check them regularly. Because the wind leaves spaces between the roof and the walls. But for others, it’s just lack of commitment, of course, these are a few cases.” (P5 HA FA-B) |  |
| Not to the recommended standard | “No, some houses were properly closed in terms of the eaves, small holes on the walls etc. but some have not been properly closed. Some mentioned that there were problems with animals such as goats that entered the house. When they planned to chase the goats inside the house, the goats would find an escape route through the screened window thereby damaging the wire gauze.” (P3 HA FA-B) |  |
| Not to the recommended standard | “Yes, in our village open eaves are properly closed. Out of 100 houses just one or two may not have open eaves properly closed maybe because the owner declined to improve their house due to misunderstandings between them and the chief.” (P6 HA FA-C) |  |
| Good standard | “The houses are well improved because we were inspecting together. We could tell each other how to work until every house has improved.” (P3 HA FA-A) |  |
| Good standard | “In my village, I am satisfied with house improvement when malaria cases are minimized following the education that we gave to households. We pick maybe 10 households and conduct an assessment, asking the households questions about malaria, and they personally tell us, “There’re fewer mosquitoes entering the house, and we have malaria less frequently.” Based on that, we say that malaria cases are reducing and that they properly improved the house. You also ask them if you can check the house, and you inspect the entire house, seeing whether the windows are properly fixed, or open eaves are properly closed. So, in my village, as an animator, my satisfaction is when people tell me and I personally see it with my own eyes.” (P6 HA FA-C) |  |
|  |  |  |

Theme: Knowledge of standards for conducting HI

| Codes | Quotes | Theme |
| --- | --- | --- |
| Sealed gauze wire | “To tell whether a house has been improved properly, we look at how the gauze wire has been fixed, checking for spaces through which mosquitoes can enter the house.” (P6 HA FA-C) |  |
| Sealed gauze wire | “A house that has been done with HI properly, would have its wire gauze properly sealed to the windows, that you cannot be able to find any space where a mosquito can have access to entry.” (P6 HA FA-B) |  |
| Sealed gauze wire | “First, we check if the gauze wire has been fixed as required, there are no spaces through which mosquitoes can enter the house” (P3 HA FA-C) |  |
| Checking for openings | “Inside the house, we check for small openings and that if there’s any light from outside then it should be through the windows only.” (P6 HA FA-C) |  |
| Checking for openings | “We have to go inside the house and see where we have an opening. If a house has been well done with HI, we should not be able to see any opening. But for a house that has not been properly closed, you find various openings be it on the roof or walls. If we stand outside and look, we cannot be able to pick up this problem.” (P4 HA FA-B) |  |
| Checking for openings | “Then we go inside and close the doors to see if there’re any spaces letting light in, and we advise them to close the spaces if we identify any. So, we tell that by doing external and internal house inspection.” (P3 HA FA-C) |  |
| Setting a demonstration house | “In our community, every house was well managed because we have set up an exemplary house called a demonstration house where people go to see how they should do their houses as well. We do inspect as well if people have done like a demonstration house” (P4 HA FA-A) |  |
| Reports and stories | “The other thing is stories that people tell that there are fewer mosquitoes entering the houses. Quarterly reports [about malaria cases] from the clinics also match with our records.” (P6 HA FA-C) |  |
|  |  |  |

Theme: Compliance with HI procedures

| Codes | Quotes | Theme |
| --- | --- | --- |
| Procedures followed | “I think houses are well improved but due to heavy winds, it destroys the window where gauze wire was nailed, this becomes a drawback. We tell the house owners to keep on improving their houses if the houses were destroyed. We inspect the houses to see if all houses are improved and once, we get a house that is not well managed, we tell them to redo the work. To add to that, if we tell the owner to redo the house and he/she has done the same, we just do the work ourselves and when we do it, we punish them because when others are building, they leave the gaps for the cats to use the gaps when getting inside the house, we do not leave any gap. We then warn them that if they continue, the issue should be reported to the village headman and tell the village headman that you are destroying our gauze wire” (P8 HA FA-A) |  |
| Procedures followed | “Um, when they have improved the house, with the windows properly sealed, they should not ignore the swamps around them. We provide them with guidance on how to empty the swamps. Because they might be spending time outside the house with no idea that mosquitoes are breeding from the swamp, will bite them and return to the swamp.” (P2 HA FA-C) |  |
|  |  |  |

Theme: Complying with standards

| Codes | Quotes | Theme |
| --- | --- | --- |
| Demonstration house | “What would encourage people to use the house improvement methods is having houses for demonstration. Committee members should serve by example by being the first to improve their houses, so we can make reference to the houses when we have meetings with people.” (P2 HA FA-C) |  |
| Exchange visits | “Being an animator, you may have to visit another animator’s area to check how the work in that area is being done. You could get some ideas from there and use them to encourage people in your village.” (P5 HA FA-C) |  |
| Capacity building for trainers | “How we could encourage people is by the committee members having knowledge about house improvement. Why? Because during training there are some animators who can’t grasp everything in a day. And if an animator does not understand what’s taught in the training, it means they won’t be able to teach committee members. And if both the animator and committee members have no knowledge of house improvement, then villagers will also lack knowledge on how to improve their houses.” (P6 HA FA-C) |  |

1. **Sustainability for HI and Works for Animators**

Theme: Notable changes pertaining to HI introduction

| Codes | Quotes | Theme |
| --- | --- | --- |
| Reduction in mortality due to malaria | “In my village, in terms of malaria cases, there has been a change. One, infant mortality has reduced. Two, the mortality of pregnant mothers has been reduced. Three, deaths of elderly people have been reduced. Why? Because in recent years people have been sleeping in properly improved houses. Today, we are just expecting new gauze wire to replace the wire that has torn.” (P2 HA FA-C) |  |
| Reduction in mortality due to malaria | “There is a change because previously, the number of children who die of malaria was high. When the malaria project started training us, there is change because when the child has a fever, they are taking that child to the hospital” (P4 HA FA-A) |  |
| Reduction in cases | “There is a change because when we are conducting the community meetings, we ask people, ‘’how many here are diagnosed with malaria?’’ Only one or 2 people could raise their hands and I believe some of the evidence is here at the hospital. We come to ask how many people from Machokero village have been diagnosed with malaria this month, there can be 2 or 3 people only unlike in the past, and more people were diagnosed with malaria.” (P5 HA FA-A) |  |
| Reduction in cases | “The issue is that before we were educated and trained on how to prevent malaria as well as usage of the interventions, we had more malaria cases. We could have serious cases of malaria that children would faint and a number of them were taken to the hospital unconscious. This time around things is better, we have few malaria cases and those that lead to unconsciousness are very few if not present.” (P5 HA FA-B) |  |
| Behavioural changes | “In my village, there has been improvement because have changed their attitudes. In the past, people had negative attitudes towards the use of mosquito nets. But today they understand the benefit of sleeping under a mosquito net and improving their houses, and they are doing things sensibly – they ask and respond to questions when they attend meetings. And they show so much interest in things that take place in the village.” (P1 HA FA-C) |  |
| Behavioural changes | “Just adding on that, previously, if someone was sick, they would rush to a healer, saying, “I have been bewitched.” But today, when they feel unwell, they rush to the hospital.” (P5 HA FA-C) |  |

Theme: Actions to promote HI participation/involvement

| Codes | Quotes | Theme |
| --- | --- | --- |
| Encouragement | “We would tell them that they should continue closing their houses otherwise if not, there would be an increase in mosquito entry into their homes. Otherwise, they will start getting sick. There are some houses that have been damaged due to the heavy rains we experienced so we have encouraged them to repair the damage and fix the wire gauze to avoid other problems with malaria.” (P7 HA FA-B) |  |
| Encouragement | “The issue is to encourage people to keep on sleeping under the net because you can happen to have no malaria plasmodium but you have received a visitor who has malaria plasmodium and if you do not sleep under the net, a mosquito can bite the one with the plasmodium and bite you, you can catch malaria” (P2 HA FA-A) |  |
| Encouragement | “I would just encourage people in my village to keep improving their houses, closing opening eaves and sealing small holes found on their houses. That’s what would help to completely reduce malaria.” (P3 HA FA-C) |  |
| Take responsibility | “The other thing is that despite the malaria project coming to an end, or already ending, my request is that the house owner personally takes responsibility to improve the house. If they need support, they can contact the animator and committee members, both of whom are right there in the village.” (P6 HA FA-C) |  |
| Educate | “What I could say to people in my village or in this focal area C is that house improvement is one approach that helps to limit entry of mosquitoes into the house. If you have fewer mosquitoes entering the house, then there will be a reduction in malaria, because mosquitoes are one insect responsible for malaria transmission.” (P6 HA FA-C) |  |
| Educate | “We will first explain to them about the dangers of malaria and then tell them that this is preventable in their households. If we explain to them dangers of having malaria, this will stick in their heads. If they stay in an unprotected place, it will be easier for them to catch malaria.” (P5 HA FA-B) |  |
| Having meetings | “We need to have village meetings so that they can act as a source of reminding the community about the goodness of having a reduction in malaria cases and encouraging them that this should continue. We then have to tell them about HI and how important it is.” (P2 HA FA-B) |  |
|  |  |  |

Theme: Ways of approaching new rollout villages

| Codes | Quotes | Theme |
| --- | --- | --- |
| Teaching | “I was trained by the project and I have a certificate. I wouldn’t have a problem standing at a meeting, teaching about malaria. I will easily teach and people will clearly understand what the dangers of malaria are. I will teach people what leads to malaria and its end result so that they understand the danger of malaria as opposed to AIDS. If I have AIDS, I will be okay as long as I am taking my drugs. But if I catch malaria today, I could die in no time. That’s what I would teach to the new villages.” (P2 HA FA-C) |  |
| Teaching | “If you are trained and not follow whatever you have been trained, you will be wasting your money, there will be no development because instead of using money on the other important things, you will be buying medication or the malaria patient can die. We should tell them how danger malaria is soot het they can change to be like us. For them to be well trained, they should be tolerant to one another and moreover, they should know that this task is voluntary based, we do not get paid, that’s how I can tell them.” (P2 HA FA-A) |  |
| Teaching | “First of all, when we get to the area, we need to explain the goodness of HI and sleeping under a mosquito net. We need to explain that closing eaves, spaces, and screening of windows will minimize mosquito entry into the household.” (P1 HA FA-B) |  |
| Describe experiences | “I can start telling them like how we did here. I can tell them that malaria starts with small plasmodium which is found in the blood and when they go to the hospital, they should be getting the same message. I can tell them that we were taught how to prevent malaria by taking part in this project. We should work with our own hands by closing all open eaves because when we are sleeping, our smell gets outside to attract mosquitos which uses the walls to get us, if we close the open eaves, we can avoid that.” (P2 HA FA-A) |  |
| Develop trust | “I think we can tell them how we started this work and the one to lead the team should have humanity because we meet with people who are mad, others are rude, as a leader, you should not be angry because if you angry, the work can no longer be successful. A leader should be patient and the one who understands. The leader should unite people and should work with the community leader (chief) because if the animator is not working with the chief, the work cannot be successful. We have been trained and we followed whatever we were taught up to this time, malaria is no longer an issue.” (P5 HA FA-A) |  |
| Describing roles as an animator | “I would clearly teach them that the animator should take lead with support from committee members and the village chief. If there’s efficient communication between these groups of people, then the house improvement project will be successful. But if there’s no collaboration then the project will not succeed. When deciding on the demonstration house, it should be the house of the leader that received training in order to teach others. That’s how clearly I would teach to them.” (P1 HA FA-C) |  |
| Describing roles as animator | “Firstly, given the opportunity I would tell them about my role as an animator, assuming that other animators from those villages are present, and then I would highlight the work we were having with committees for HI when we were going through the villages.” (P6 HA FA-B) |  |
| Explain challenges | “Just adding on what P1 and P6 just said, for the new villages around the Majete Game Reserve that had no implementation of the project, um, what I would teach to them is about challenges: “When you are doing house improvement, there are different challenges. Therefore, you shouldn’t stay idle, thinking the project will solve challenges. It’s better to sit down and discuss so that the project runs effectively. The problem is particularly with committee members. Whenever someone joins a project or starts volunteering, they have expectations, and sometimes their expectations are not met. So, they may stop prematurely, or say discouraging things to the villagers, “There’s no benefit in that.”” This is something that can be addressed through dialogue. There should be a working relationship between the animator, village chief and committee members, so that should there be any challenges, they can dialogue.” (P6 HA FA-C) |  |
| Intended purpose | “The other thing I could mention is that house improvement is not for fun – we are not improving the house to make it look beautiful only, no. The idea is to limit entry of mosquitoes into the house. Mosquitoes are responsible for malaria transmission, and if their entry into the house is reduced, it means there will be a reduction in malaria.” (P6 HA FA-C) |  |
|  |  |  |

Theme: Project’s future after MMP

| Codes | Quotes | Theme |
| --- | --- | --- |
| Continuation (Skills acquired, knowledge on malaria risk, cost) | “I think this project will continue because we have skills and community members now know the danger of malaria, so it cannot stop because it is protecting our lives and we are not wasting money for medication on the malaria” (P5 HA FA-A) |  |
| Continuation (Habitual) | “I think that we will continue with the work because a lot of people now are used to sleeping in houses that have HI in them, so with this development they will continue with HI.” (P4 HA FA-B) |  |
| Continuation (Malaria risk reduction) | “House improvement will continue. After the Majete Malaria Project phases out, we’ll continue to work in the villages, protecting our lives from malaria infection through sleeping under a mosquito net, closing open eaves and sealing holes on the house. We’ll do all this for the protection of our lives and of our children.” (P1 HA FA-C) |  |
| Continuation  (Malaria risk reduction) | “It will continue because people have seen the goodness of HI, there are few malaria cases being encountered. If gauze wire is not available some have got used mosquito nets, so these can be used for screening windows instead.” (P3 HA FA-B) |  |
| Continuation | “Majete was a light for us because they are wishing us the best on our lives and we cannot stop because in the end, we shall die and others will have to carry on with the skills. We thank you for this program and we should not stop doing it because our children should also be taught about this.” (P2 HA FA-A) |  |
| Continuation (Malaria risk reduction) | “The work will continue because its benefits are not seen by the project but by villagers themselves. So will carry on with this work to protect friends and families that are at risk of malaria.” (P3 HA FA-C) |  |
|  |  |  |

Theme: Roles as animators

| Codes | Quotes | Theme |
| --- | --- | --- |
| Continuation  (Skills acquisition) | “I will continue doing this work. I won’t stop when the project stops. I will encourage people to improve their houses, sleep under a mosquito net, empty swamps, and break pots with unused water for safety from this dangerous female mosquito. I will continue doing this because I am the one in the village with skills for this work.” (P2 HA FA-C) |  |
| Continuation (Skills acquisition) | “We will continue because we have acquired skills in this work, so even if we do not want but the fact that we acquired those skills for a long-time people will still be finding us pertaining to problems of HI and other issues to do with malaria.” (P2 HA FA-B) |  |
| Continuation (Skills acquisition) | “If Majete leaves us alone, as volunteers we shall continue doing the job because we have skills and we have learnt a lot and we cannot stop training people because Majete has left us.” (P8 HA FA-A) |  |
| Continuation (Known the benefits) | “The work will continue because the Majete and the Hunger project are not doing these for themselves but for us. When we are doing this work, we should know that we are doing for the sake of our lives to have enough protection, so we will continue.” (P7 HA FA-A) |  |
| Continuation (Happy with the role) | Yes, we will continue the reason being that I like being an animator as I am able to clarify issues concerning malaria when I have been approached in the village even before the day of the meeting. (P5 HA FA-B) |  |
| Continuation (Skills acquisition, Collaborations) | “As P3 alluded to, our roles [as animators] will continue because the project mostly used us, villagers, to run the activities – they were not coming to teach people in the village – we were teaching people ourselves. So, we have the skills. The other thing is that health workers were already teaching in the villages about malaria and other health issues before the project came, and through the project, we have formed a collaboration with them. So, where the animator is weak, they will be there to teach about malaria.” (P6 HA FA-C) |  |
|  |  |  |

Theme: HI committee continuation

| Codes | Quotes | Theme |
| --- | --- | --- |
| Continuation  (Village Appointments) | “Yeah, committee members will continue their role even if the project closes out. Why? Because the committee was appointed by villagers – it was not appointed by an animator or the project or committee members themselves, no. If the committee does not function properly, there are supervisors responsible for its operations. There’s a village chief supervising it. There’s an animator supervising it. Committee members are also committee supervisors themselves. So, if the committee is not functioning, the different supervisors can dissolve the committee and replace the members with new members. So, the role of the committee will continue.” (P6 HA FA-C) |  |
| Continuation (Knowledgeable, skills acquisition) | “I think these people will continue working because they have been trained and they knew the truth that the work is voluntary based, so they will have to continue with this up until malaria will no longer be the issue here” (P4 HA FA-A) |  |
| Continuation (Supervisory structure in place) | “The committee will continue performing its role. Why? Um, we have the village chief, who’s responsible for the supervision of how the committee is operating. So, should any members slack in their performance just because the project is phased out, it will be the responsibility of the chief to appoint new members. In that regard, the committee will continue its role, it can’t stop on the basis that the project phased out, because there are new members taking over.” (P3 HA FA-C) |  |
| Continuation (Supervisory structure in place) | Our work will go on even if we have one committee member who is not willing, we can sit down with the chief to choose another person to replace that one, even if two people are not willing, we shall replace them with others who are willing to do the work. I hope the work will continue. (P2 HA FA-A) |  |
|  |  |  |
