# Supplementary material for: Assessing the implementation fidelity, feasibility, and sustainability of community-based house improvement for malaria control in southern Malawi: a mixed-methods study
Source: BMC Public Health. 2024 Apr 2;24:951. doi: 10.1186/s12889-024-18401-4 (PMC10988826; doi:10.1186/s12889-024-18401-4)
Supplement: Supplementary file 8 — Supplementary Material 8 [file 12889_2024_18401_MOESM8_ESM.docx]

**Assessing the implementation fidelity, feasibility and sustainability of community-based house improvement for malaria control in southern Malawi: a mixed-methods study**

**Supplementary File 8: Deductive Coding (Code reports for fidelity, feasibility, and sustainability of HI)**

**Description:** Describes participants’ views and perceptions on how feasible the implementation of HI as a malaria prevention intervention is.

**Abbreviations**

FGD – Focus Group Discussion

IDI – In-depth Interview

KII – Key-informant Interview

<Files\\Chief Maganga 2 Chichewa KII> - § 5 references coded [9.53% Coverage]

Reference 1 - 3.13% Coverage

Respondent: It requires materials which the MMP brings to animators, and these also have got committees which brings these materials. So, they go at each and every household and take measurements for windows and wires according to the window measurement, anywhere there is a deliberate gap for air entry, they close so that mosquitoes do not get in.

Reference 2 - 1.55% Coverage

For closing eaves, one requires bricks and builders, for closing windows, wire is used. Also hammers, measuring tape, and also the house owners need to buy nails for the wire

Reference 3 - 1.05% Coverage

The materials are found in time if the owner reports to the committee on time, the committee also responds on time.

Reference 4 - 0.62% Coverage

Respondent: For nails, the owners of the house purchase themselves.

Reference 5 - 3.18% Coverage

Yes, they are ready. Because the committee was given power. So during village gatherings they teach the people that one day hand out materials will be no more, so it will not be proper to stop experiencing the goodness of the strategy just because there are no hand out anymore. But people should help themselves just as the hand outs are helping them.

<Files\\Community participant chambo IDI> - § 4 references coded [9.29% Coverage]

Reference 1 - 1.51% Coverage

**INTERVIEWER:** want to go to another section. What resources are needed for house improvement?

**PARTICIPANT**: hammer nails mud small bricks

Reference 2 - 1.53% Coverage

**PARTICIPANT:** for the hammers we were been given by the government, for the nails some are been given and some we buy when it is not enough

Reference 3 - 4.23% Coverage

**INTERVIEWER:** thank you, do you think people in your village are willing to buy gauze wire on their own?

**PARTICIPANT:** based on the productivity of the wire people can buy and the house looks smart. Some people can manage based on the productivity to them.

**INTERVIEWER**: thank you so much. Do house improvement activities make you fail to work on your personal things?

**PARTICIPANT:** no

Reference 4 - 2.02% Coverage

are the people in the community willing to pay for improved quality housing to prevent malaria? *(Repeating the question)*

**PARTICIPANT:** yes, they can accept based on life standards

<Files\\Community participant Chaziya IDI> - § 4 references coded [10.71% Coverage]

Reference 1 - 1.80% Coverage

**PARTICIPANT:** the most important things are mud/sand and bricks

**INTERVIEWER:** anything else?

**PARTICIPANT:** if the house used cement, so cement, plaster; so that mosquitoes should not use space at the wall

Reference 2 - 1.70% Coverage

(Short pause) like at the windows any materials

**PARTICIPANT:** for the windows we use gauze wire

**INTERVIEWER:** any other materials

**PARTICIPANT:** I can say mosquitoes but we don’t consider that much

Reference 3 - 3.66% Coverage

**PARTICIPANT:** these materials sometimes are available on time. We should say they are available on time

**INTERVIEWER:** alright. So how do you find these materials?

**PARTICIPANT:** mostly we find them through malaria organisations. It helps us in the village and we don’t find problems because it gives us gauze wire and sometimes chemicals to spray the wells so that mosquito should be absent. So, this organisation helps us a lot

Reference 4 - 3.56% Coverage

**INTERVIEWER:** So, I want to hear from you, do you think people are ready to buy gauze wire on their own?

**PARTICIPANT:** aaa!

**INTERVIEWER:** suppose the Majete team has left us

**PARTICIPANT:** they are not ready. Most people here we are not working, we are poor so to think of wire is very difficult. Now we are very lucky with this organisation, on our own is difficult because understanding is also difficult

<Files\\Community participant chaziya-chapananga IDI> - § 3 references coded [5.18% Coverage]

Reference 1 - 1.55% Coverage

**PARTICIPANT:** things are like once finishing building the house should you should close the eaves and put gauze wire on the window so that we can prevent malaria

Reference 2 - 1.81% Coverage

**PARTICIPANT**: materials like frame, iron sheets timber doors cement.

**INTERVIEWER:** these materials are they available on time?

**PARTICIPANT:** they are available on time when you prepare

Reference 3 - 1.81% Coverage

**INTERVIEWER:** do you think people are ready to buy gauze wire on their own?

**PARTICIPANT:** with the goodness of it we have experienced people can buy to protect their life from malaria.

<Files\\Community participant Goliati IDI> - § 2 references coded [1.85% Coverage]

Reference 1 - 0.59% Coverage

**PARTICIPANT:** they use building knife and bricks and sand

Reference 2 - 1.26% Coverage

**PARTICIPANT:** no there are only those materials. The small opening, we close with mud if there is big opening we use bricks

<Files\\Community participant Kabwatika IDI> - § 4 references coded [6.81% Coverage]

Reference 1 - 3.91% Coverage

**PARTICIPANT**: It involves digging the mud/sand and closing the eaves and if the house has the holes at the wall, they advices us to close them so that the mosquitoes should not find any chance of entering the house. Once you do that sleep in the mosquito net.

**INTERVIEWER**: how does the work of house improvement be like? Do you look at tiresome work or not?

**PARTICIPANT**: it is good, no any problems associated with it. It doesn’t have hard labour since you are working at your house and you will work as one of the house activity/chores

Reference 2 - 0.40% Coverage

**PARTICIPANT**: hoe, shovel, building knife are required.

Reference 3 - 1.08% Coverage

**INTERVIEWER**: do you think people are ready to buy gauze wire on their own? Beside you and village members are…

**PARTICIPANT**: no, we cannot manage

Reference 4 - 1.41% Coverage

**INTERVIEWER**: are the people in the community willing to pay for improved quality housing for preventing malaria? *(Repeating the question)*

**PARTICIPANT**: yes they can accept without any problems

<Files\\Community participant Liwonde IDI> - § 1 reference coded [0.79% Coverage]

Reference 1 - 0.79% Coverage

**PARTICIPANT:** resources? Bricks water and other things are required

<Files\\Community participant Machokero IDI> - § 1 reference coded [1.45% Coverage]

Reference 1 - 1.45% Coverage

you use bricks and mud closing the eaves if you don’t have brinks but you want to close the eaves you can use the stones and mud as well

<Files\\Community participant MAGANGA IDI> - § 1 reference coded [2.44% Coverage]

Reference 1 - 2.44% Coverage

**PARTICIPANT:** for the bricked house it is too involving, it involves a lot of activities

**INTERVIEWER:** could you explain

**PARTICIPANT:** we need to make other bricks and gather them at the appropriate place that is when we spend a lot of money. We find the builder to build the house smartly and we find other person to do inside plaster then close the eaves but without doing these then it will be grass house with sticks

<Files\\Community participant Mtemera IDI> - § 3 references coded [3.83% Coverage]

Reference 1 - 0.63% Coverage

**PARTICIPANT:** using bricks, like building and on top put mud so that there should be no space

Reference 2 - 2.34% Coverage

**PARTICIPANT:** for the wire; first they do measurement tallying wire and window measurements and they give them to people and they put it for themselves (malaria team). They leave the house after everything is done accordingly. For the eaves they tell the owner of the house to do, then they supervise, if it has not done in order they advise them to collect

Reference 3 - 0.86% Coverage

**:** suppose the Majete team is phasing out, people are they ready to buy gauze wire on the own?

**PARTICIPANT:** they are not ready

<Files\\Community participant Weremu IDI> - § 1 reference coded [0.55% Coverage]

Reference 1 - 0.55% Coverage

**PARTICIPANT**: this work cannot make us fail to do our personal work

<Files\\FA-A Animators FGD> - § 5 references coded [10.69% Coverage]

Reference 1 - 4.09% Coverage

previously, we thought the job is too much but when we started doing it, we realized that the work is simple. We accept to do the work and it became simple

**Facilitator:** In what way did see this job to be tiresome previously?

**P8:** Because we were thinking that we could not manage to talk to people and listen to what we train them. When we train people, it has been simple method because everyone is taking part and is working on their own. Once we tell anyone that we are coming tomorrow with the gauze wire, you should close the open eaves, in the next morning, we find them that they have done the work.

**P2:** We did not know how important this method is as far malaria prevention is concerned. Once we noticed that this work was good, malaria is not spreading, we are feeling good. We are able to discuss with our wives that once they have noticed some gaps, they should close it if men are away to do the piece work

**P5:** In the past, this was not easy job to do because when building the house, we were leaving the open eaves unclosed. When this program came, it was not easy, thinking on how to get the bricks and close the open eaves, it had been the tough task. With the sensitization meetings that we were conducting in the communities, the work started becoming simple because everyone understood that once they have done the job, they will protect his/her life and the life of their children

**Facilitator:** As we proceed, I would like to hear from you, was the job tiresome or simple?

**P5:** As animator, the job is simple because we work as a team together HI committee. Since we work as team, the work is no longer tiresome

Reference 2 - 2.91% Coverage

What are the materials needed when doing house improvement task?

**P1:** you need nails, scissors, a hummer, so that the work should be simple

**P2:** Just to add, we need a tape measure for measuring the window to see how much gauze wire do we need

**P3:** This work, we need all necessary materials for this work to be simple. We need bricks, water, building knife, gauze wire, a scissors, tape measure and the hummer so that the builder should get it easy

**P8:** we need a brick maker (chikombole) so that we should be able to tell how many bricks are needed

**P6:** we need a level when building

**Facilitator:** Are all materials that you have mentioned available in time?

**P7:** Before the work, we gather all materials so that after we finish, we should put the gauze wire

**P5:** In most cases, we first go and measure the windows to see how much gauze wire is needed, for example in 50 houses, so materials can be available in different times. So we first measure the windows and submit the figures wherever it’s needed

**P2:** sometimes we find a person has already build a house and is remaining with closing of open eaves. We just measure the windows and put the gauze wires

Reference 3 - 1.57% Coverage

I think there is no one in our community who can afford to buy the gauze wire because the money that we get in the villages is too little and the gauze wire is expensive

**P4:** I think it is possible based on how someone has welcomed it and how one thinks on malaria prevention, he can spend even a lot when protecting his/her life

**P5:** Some can afford, some cannot afford because we have different economic status

**P8:** I think some can afford and some cannot afford because we get money differently, others get money easily and yet don’t get money easily. We cannot promise that people in our community can buy gauze wire easily

Reference 4 - 0.85% Coverage

for us it does make us not to do other work because we have a time table. We can do our work in the morning and around 2, we can do the house improving work

**P5:** This cannot make us not do our work because community meeting are not conducted daily. If it is time for community meetings, we do them and the rest of the days, we do our work

Reference 5 - 1.28% Coverage

Can people in your community pay in order for them to be staying in the modern improved house and prevent malaria?

**P7:** some can manage and some cannot because we have different financial status and because of that, the only person who can afford is the one who has money. As for me, I can manage because I have seen the advantage of this.

**P5:** some elderly people cannot manage and they need someone to come and help them. For others, like those who do businesses, they can afford because they get money often

<Files\\FA-A Community participants FGD 2> - § 21 references coded [17.86% Coverage]

Reference 1 - 0.29% Coverage

The materials required for closing of eaves are water and soil. We make mud and close the eaves. Thank you

Reference 2 - 0.31% Coverage

Besides that, we mix water and soil to make mud. Then we take bricks to close the eaves. We use builder’s tool.

Reference 3 - 0.27% Coverage

What is required is to find gauze wire to use close the eaves in the all the houses being built.

Reference 4 - 1.33% Coverage

This work is still difficult because some people are old that they cannot manage to close the eaves and tack the gauze wire. Instead, we do help them. We close the eaves and tack the gauze wire for them.

**Facilitator** Alright. Thank you, P3.

**P5** This work is really difficult. Adding on to what P3 has said, there are the elderly who do not manage closing the eaves and tacking gauze wire. When the committee members are distributing the gauze wire, they are the ones who help them.

Reference 5 - 1.65% Coverage

It is not a difficult work. We go there as a group, the committee. For the elderly, we tack the gauze wire for them. For young ones, we tell them to find nails and tack the gauze wire. If they say they do not have the nails, we cut bicycle spokes into small pieces to use tack the gauze wire. In one day…**speaks while laughing *(don’t laugh at me…)*** we go to all the houses. If we don’t finish, we go the following morning. We finish tacking the gauze wire off we go. We do not really get tired. If we find someone old, we do for them. If we find someone energetic, they tack the gauze wire themselves.

Reference 6 - 0.62% Coverage

The materials required for this work; a hoe, level to straighten the wall, builder’s knife. Sure.

**P5** The materials that are required; mud, bricks, builder. After the house is completed, measuring tape, gauze wire and hammer.

Reference 7 - 0.74% Coverage

The materials that are required are water as well as pair of scissors for cutting the gauze wire.

**Facilitator** Alright. Are there, may be, other materials that have been forgotten?

**P4** Thereafter, you should also tack a mosquito net so you should be seeping under it.

Reference 8 - 0.29% Coverage

One additional thing. There is also need to be nails for tacking the gauze wire after closing the spaces.

Reference 9 - 0.08% Coverage

Yes. A hammer was forgotten.

Reference 10 - 0.68% Coverage

These materials cannot all be available at once. You start solicit one at a time. For example, if you to construct a brick house, you cannot have the bricks, hoes and the builder all at once. So, the thing is we solicit these materials one by one.

Reference 11 - 0.72% Coverage

When you want to construct a brick house, the first materials needed are a builder, bricks, mud, measuring tape and builder’s knife. When you are done with constructing the house and closing the eaves, you will need a measuring tape, hammer and the gauze wire.

Reference 12 - 0.92% Coverage

When you are building a house, the materials are with the builder. When the house is done, we close the eaves without even using a measuring tape. But when we are tacking the gauze wire, the committee members do bring their measuring tapes as well as the pair of scissors to cut the wire. Then they leave it all to us to tack the wire.

Reference 13 - 0.50% Coverage

Tape, pair of scissors and wire are available when we have finished building the house and we want to close the windows with gauze wire. That’s the time these things are available.

Reference 14 - 0.95% Coverage

These materials to be available in time, it is good to tell the committee members to bring the wire so that we can close the eaves before we start sleeping it. If we have already built the house or the wire is getting damaged, we should tell the committee members to bring another gauze wire to close the eaves before it is completely damaged.

Reference 15 - 1.96% Coverage

What is needed is to find the materials. When they are available, you can tell the builder to start building.

**Facilitator** So where exactly do you find the materials?

**P4** Maybe buying the materials, if you do not have them, for the builder to start building.

**Facilitator** Buying?

**P4** Yes.

**Facilitator** Alright.

**P5** Some of the materials for tacking the gauze wire are brought by the committee members. Things like hammer, pair of scissors and tape.

**Facilitator** Alright. How about other materials? I really want you to open up and explain.

**P6** Some of the materials we have them already. Things like hoes and water. We borrow of the things from the builder when we want to close the eaves. Then we return them.

Reference 16 - 0.73% Coverage

Soil can be used for closing small spaces.

**Facilitator** Alright.

**P5** The materials are water, a hoe and soil. You mix water and the soil, and take the mixture to close the spaces.

**Facilitator** Alright. P7?

**P7** The materials we use are water and water to make mud.

Reference 17 - 0.41% Coverage

Some can manage while others cannot.

**P4** There are …who cannot buy or they are lacking money to buy the gauze wire. I think if they can get helped…

Reference 18 - 1.30% Coverage

People are ready to buy on their own regarding that malaria kills. But still, it is difficult for some people to find gauze wire on their own due to financial problems and the fact that others are old. However, for the people who can manage are ready.

**Facilitator** Alright. Any other opinions?

**P3** They are ready to buy gauze wire on their own because the money they can spend on buying the gauze wire is less than that which they can spend to take someone to the hospital.

Reference 19 - 1.00% Coverage

It does not prevent us from doing our household daily works. We do this work maybe in a day while the other daily works are done on several days. There are no any other issues that make us not to do household works.

**Facilitator** Alright.

**P5** This work is not difficult so it cannot prevent us from doing other works. It is also the work that protects our life.

Reference 20 - 0.68% Coverage

The work is not difficult because it makes to be happy people in our households.

**P6** The work is not difficult because it is the work we can include in our daily works. So it cannot prevent us from doing other works because it is already on plan.

Reference 21 - 2.44% Coverage

They can so that they have improved houses to prevent malaria. This is because they will save more money that can be spent when they fall sick.

**Facilitator** Alright. That’s P3’s opinion. L2? Let us perhaps start with P4.

**P4** It is possible because it everyone’s protection. Everyone has to sacrifice themselves to pay the money.

**P2** It is to lose money rather than losing a life. This is because the money is little. They cannot buy a life, for example child’s or mother’s life, rather than paying the little money to protect somebody’s life. Thank you.

**Facilitator** Okay. Let us go to the last section. I would like to know from you as people coming from villages which are doing House Improvement work. I would like to hear from every one of you in your village. Do you think all the houses in your villages are properly improved?

**P3** The houses in our village are properly improved.

<Files\\FA-A HI Committee FGD> - § 5 references coded [17.57% Coverage]

Reference 1 - 1.37% Coverage

just to add, we need bricks, cord and the building knife. Then we make sure that we have everything needed so that the owner should not regret when we have done the work.

**P3:** what is needed when doing the work is gauze wires, tape measure, and the hummer so that these can help us

**P8:** what is needed is bricks, water, level, building knife, builder’s and then we go for the work

Reference 2 - 1.91% Coverage

would like to know from you as committee members. Apart from closing of open eaves and gauze wires, what are the other methods that people can use for house improving?

**P3:** after we were trained this procedure, we welcomed this and we sleep under the nets, therefore, we don’t have any option other than these

**P1:** just to add, if we don’t have gauze wires, I saw in Mozambique, they don’t have such gauze wires, they use nets inn their windows as gauze wires

**P5:** if there is no gauze wires, we should use the nets in the windows

Reference 3 - 11.91% Coverage

What are the materials needed when doing the house improving work?

**P3:** When going we have to take bricks, knife, level and a cord. We then get mad and water

**P4:** Other materials are like gauze wire, tape and nails

**P6:** When doing house improving, we need gauze wire, tape, hummer and nails

**P7:** we need scissors for cutting gauze wires, then we need a hummer and nails, thank you.

**Facilitator:** Okay, you have mentioned different materials, I want to know from you, are these materials available in time?

**P8:** The materials are found at different times because when going, we don’t carry materials like gauze wires and the hummer. When we have done the job, the other day we go with only tape, ball pen and the exercise book. When we have done the measuring, we go next time to with gauze wires and the hummer, thank you.

**P1:** materials are found at different time, like bricks, they are needed at a time we close the open eaves while the gauze wires and other materials are needed when going to close the windows, thank you.

**Facilitator:** Are these materials available at once but you use them at different stage, is what you mean?

**P5:** materials are found at different stages like when we want to close the open the open eaves, we need water, mad and the knife and this is before gauze wires, thank you

**P2:** some of these materials we as committee members we don’t have material like gauze wires, the materials that we have are like hummer, tape and scissors but then we lack materials like level and knife

**Facilitator:** what can be done for these material to be readily available in time?

**P2:** I believe if we can buy these materials in such a way we did with the tape and the hummers, the committee then should be responsible for the materials, it can be better.

**P3:** sometimes when we have gone to do house improving work, it can happen that the person has no knife, level and the cord. We have to go to a builder who has such materials to ask for these materials so it’s time consuming.

**Facilitator:** You have mentioned a lot of materials, I would like to know, how you and the community members get such materials?

**P8:** there are some materials that the owner get himself like bricks. Some materials like level, a community member does not have and we don’t have them as well. Such material we go to the builder to borrow.

**P1:** materials are found at different times especially materials that are used for closing the open eaves. For example our committee, we don’t have a committee member who is also a builder, we need to go to the builder to borrow the material and some times we told the owner to get the builder and borrow.

**Facilitator:** Apart from these materials that you have mentioned, are there other materials that you think are necessary when closing the open eaves?

**P1:** In the small holes, we need only mad to close them

**P3:**  we need glue and saw dust for the door that has holes, thank you.

**Facilitator:** Do you think people are ready to buy gauze wires on their own when Majete project phases out?

**P7:** It seems people are ready but then we have different financial incomes in the communities, some people can afford but some cannot.

**P8:** Just to add, some can afford, some cannot afford, so it can be difficult.

**P3:** As others have said, some can afford, but majority cannot afford

Reference 4 - 1.08% Coverage

would like to know, is this house improving work makes you fail to do your daily activities?

**P6:** No, this does not make us not to do our daily work because we don’t do every day, thank you.

**P5:** We do not do this work of house improving daily, so every time we do our house activities, thank you.

Reference 5 - 1.31% Coverage

So lastly, I would like to know, can people pay money in order for them to have their houses closed in the open eaves as one way of preventing malaria?

**P8:** people can pay money because they will know that if they catch malaria, every activity is at the household cannot be done

**P5:** It can done because we have taught people on the advantage of this, thank you

<Files\\FA-B Animators FGD> - § 6 references coded [14.86% Coverage]

Reference 1 - 2.40% Coverage

house improvement activities include, some houses that are built with no frames, so we advise them to insert a frame so that they don’t have problems closing the window with gauze wire. Then there are others who build without leaving a window, they just drill holes on the wall, so we advise them to seal those holes with mud, so that mosquitoes find no entry into the house.

P4: most people build houses without closing open eaves, so, as animators, we encourage them to close those open eaves, so that mosquitoes have no entry into the house.

I: anyone with anything else?

P5: we also encourage them, because many don’t close open eaves when building their houses because of running short of bricks. So, if there are say 5 people, we encourage them to mold and burn bricks together for closing open eaves, because sometimes they tell that “I don’t have bricks.”

Reference 2 - 2.92% Coverage

um, what comments and expectations did people have about house improvement in the beginning?

P4: um, in our village, people had comments on the benefits of closing open eaves and sealing small openings, because there were now fewer mosquitoes in their houses than previously. So they would comment, saying, “Honestly, things are now better than before.”

P1: the expectations that people had were that the project was still going to provide gauze wire after phase out, they were wondering where the gauze wire was going to come from if the project had phased out, because the wire they had received was now rusting and wearing out.

P6: when people had received training on how to use gauze wire, and when they had given us gauze wire for demonstration to the villages, people had expectations that were also going to give them nails and hammers for nailing the wire. But when we explained to them they understood and would positively comment, saying, “Gauze wire is useful, it’s also stopping other small flies than mosquitoes from entering our house.”

Reference 3 - 0.82% Coverage

what she’s saying is a good idea, but plastering the walls will require us telling them how to mix cement with sand. But another way of closing openings or closing windows that we could teach to people is mixing sand with mud, we women call it ‘kutsutsuta’. That could be an alternative method.

Reference 4 - 1.60% Coverage

bricks for closing open eaves, gauze wire, nails and reeds for nailing the gauze wire.

P6: materials? You need to look for bricks or broken bricks for closing open eaves. If you receive gauze wire, or if you buy gauze wire yourself when the project phases out, you need to look for nails, a hammer, or a good stone for a hammer, and pieces of timber for sticking edges of the wire to the wall, then plastering the edges of the window so that the gauze wire does not come out.

P1: if bricks are not available, you can use stones for closing open eaves.

P5: you also need mud.

Reference 5 - 5.45% Coverage

okay, are these materials readily available?

P4: these materials are not readily available. Like bricks it was difficult to find them during the rainy period. Nails are particularly hard to find because we have to buy them. So it is difficult to find the materials at the right time.

P5: they are not readily available because if we visit someone and find that their house does not have open eaves closed, then there are a number of things that are required at that time. To have the open eaves closed it means they have to look for bricks. The same goes for nails. In short, they are not found at once – it takes time to gather them.

I: okay, what could be done to make the materials readily available if they are not readily available?

P5: before we ask for gauze wire, we need to talk to people about closing open eaves, so that everyone works on their houses at the right time, before rain starts – because malaria prevalence is higher during the rainy period, when there are plenty of breeding spots.

I: anyone with anything else? [Silence] okay. How do you find materials for house improvement?

P2: we find materials for house improvement through various ways. Some of the materials we buy them. Nails for nailing gauze wire for example, we have to buy them. But for bricks we can mold them ourselves and use them.

P7: sometimes we look for broken bricks, or you go to someone and ask for them, and you use them. For nails, some get them from well-wishers, but not many give them for free, you have to pay.

P6: some of the materials like a hammer, scissors, a tape plus gauze wire were being supplied by the office, Majete malaria project. But other materials like bricks, nails people have to look for them themselves.

P3: just as my colleagues mentioned, gauze wire and a hammer would come from the office. But nails we buy themselves ourselves. Mud we prepare it ourselves. Bricks we mold or look for them ourselves. That’s how we find the materials.

Reference 6 - 1.68% Coverage

okay, in your view, do you think people are prepared to buy gauze on their own? If yes or no, explain.

P4: for people in our area, it’s not possible for them to buy gauze wire on their own. They can’t buy because of lack of money.

P1: it’s true people in the village can’t buy gauze wire on their own, only those that have money can buy. But if the wire is expensive people can’t buy it.

P7: it’s hard for someone to buy gauze wire with their own money because the wire costs a lot of money. If someone fails to buy a nail that costs 50 or 100 kwacha, they can’t buy gauze wire given how expensive it is.

<Files\\FA-B community participants FGD> - § 9 references coded [23.52% Coverage]

Reference 1 - 3.68% Coverage

**Facilitator** Thank you very much. So, I would like to hear your opinions. Why do people leave open eaves in the houses? Let us perhaps start with Z1. ***(Question repeated for clarity)***.

**Z1** They leave the spaces because when they are constructing the house, they do not close the eaves right away. After finishing constructing, they then close the eaves.

**Facilitator** Alright.

**K2** May add to that? Leaving the eaves open is not the builder’s intention. As you know these works here in village are different from those done by the government**.** When constructing the government houses, they close the eaves before roofing. The problem we face is that when you want to construct a house, you will have to find a builder who will leave the eaves unfinished. Then, you will need to solicit money for roofing and later on, you will have to find money again to have the eaves closed. So, when you don’t have money, you just leave the eaves open through which mosquitoes enter the house. Leaving the eves open is not intentional. It is because building a house is not easy here in the village. That is why we leave the spaces.

**Facilitator** Alright. Perhaps we can say it is expensive for someone to build a house, as you are saying.

**K2** Yes.

**Facilitator** Alright. That’s K2’s opinion. C1? ***(Question repeated)***.

**C1** It happens that the owner of that house perhaps is not well prepared. When you hire builder, you can tell to finishing closing the eaves when roofing on the same charge you have agreed. However, if roofing is done separately, for example a year later, after the wall, the eaves are left open. That’s what happens.

**Facilitator** Alright. K3, do you have additions on this?

**K3** You leave the eaves open because you do not have enough bricks. Sometimes, it happens that you have done the wall and you have used all the bricks. In the end, you leave the eves open because you do not have enough bricks to finish everything.

Reference 2 - 1.48% Coverage

Alright. On the same issue, I would like you to explain to me the works that are done during House Improvement. When we say we are improving our houses, what are the tasks that are done?

**C1** Firstly, there is closing of eaves, putting glasses in the windows, tacking gauze wire, door, plastering and others works that are done when a house has been finished. Thank you.

**Facilitator** Alright. Are there any other opinions? K4?

**K4** No any other opinions.

**Facilitator** Is there anyone with other opinions on this? K2?

**K2** The other thing is an addition that the most important is to tack the gauze wire, and make sure the doors do not have any spaces in them through which mosquitoes can enter into the house. That is why there is need to find a carpenter who can help us.

Reference 3 - 1.60% Coverage

Alright. Are there any other expectations or issues concerning this HI project that people were talking about?

**K4** This method was a little difficult in our village at first because people were not understanding it. However, after they had been taught by the animators, people started developing interest to take part in this work. At first people were complaining that when they tack the gauze wire, it gets hot inside the houses. As a result, some people could remove the gauze wire and never tack it again. However, with the teaching and seeing the advantages of this work in the village in as far as malaria is concerned, they realised that it is important to tack gauze wire. There is been a reduction in the cases of malaria reaching this far. There is been a large decrease because people have taken part. Thank you very much.

Reference 4 - 1.12% Coverage

Alright, thank you very much. Let us proceed. Apart this method of closing spaces with mud and windows with gauze wire, what are other methods that can be employed in House Improvement? I only would like to get your opinions that apart from closing windows with gauze wire and closing eaves with mud, are there any other methods that people can employ in House Improvement?

**K4** The other method which can also help in House Improvement is to make sure we take care of our house so that it does not get damaged and have spaces again. So maintenance can also help us a lot. Thank you.

Reference 5 - 3.35% Coverage

Thank you. I would also like to know little more especially on how the work is done. K4, you already started. I would like to know if the work is fine or tedious?

**K4**  The work is just fine, and not tedious because you can plan that today you do one side and the other side on the next day as you see fit. So, we cannot say it is tedious. The work is just okay. Thank you.

**B1** I just want to add on the work. It is true that the work is not tedious. For example, as the committee members that go around tacking gauze wire, you plan how many houses you are doing on a day. So, you only target the houses you have planned to do. For example, you can say today we are doing ten houses. It means today you are doing only those ten houses. The next day you can do ten or fifteen houses until you finish all the houses that can be done according to the gauze wire available. So, the work is easy and good. It is not tedious. The most important thing is to discuss how you are going to do the work.

**Facilitator** Thank you. Are there any other additions?

**K2** Mine is just a word of encouragement because the work that the animators do is voluntary. This is because people say a lot of things when they are moving around helping people, especially the elderly. They say that they have left their works going around helping the elderly. So, when we hear that we have to be brave that we need to do the work of helping the elderly because in future someone else will help us. This is because the elderly do not choose to be weak. It is because they have lived for long time. I am talking this because there are old people in our villages who cannot manage the work. So we need to be brave even if we are taunted because some people say this out of ignorance.

Reference 6 - 7.24% Coverage

Thank you. So, let us continue on this issue; what are the materials that are required during House Improvement? If you may mention the materials that are required during House Improvement. I can see K3. ***(Question repeated)***.

**C2** The materials required during House Improvement work firstly are pair of scissors, then measuring the length of windows and getting nails as well as a hammer or anything to use for nailing the gauze wire.

**Facilitator** Are there any other equipment, K1, that you would need to add?

**K1** The addition thing is that when we are tacking the gauze wire, we use fanta bottle tops to prevent the gauze wire from detaching from the wall to prevent mosquitoes from entering the house.

**K2** Additionally, we also use small pieces of wood to help attach the gauze wire to the wall. Of course, the use of bottle tops is possible but there may be space between one bottle top to another. So, you just buy a small wood (thimba) to attach the gauze wire to the wall and start nailing. That way, mosquitoes cannot even try to.

**Facilitator** Z1, do you have any other materials that are required during House Improvement?

**Z1** Alright. In the windows for example they put some things to prevent them from opening.

**Facilitator** Alright. Is there anything else?

**K4** When closing eaves there is need for enough bricks so that no spaces are left. Thank you very much.

**Facilitator** So, one other thing that I need to know is that are all these materials you have mentioned, like pair of scissors, gauze wire, thimba, available in time of need?

**K4** Pair of scissors and measuring tape are available in time because the people who distribute the gauze wire do have these materials. So, it is easy to find; when they are moving around distributing gauze wire, they do have these materials with them. Thank you.

**Facilitator** Alright. Are there any other additional opinions on this issue?

**B1** What K4 is saying is true that when they are moving around measuring the dimensions of the windows to put the gauze wire, they carry all these materials with them. So, it is not difficult because they themselves cut the gauze wire depending on the measurements taken. It is not difficult because they have every required equipment with them.

**Facilitator** Alright. Are there any other opinions that can be added on this issue? In continuation, I would like to know how you these materials that are used in House Improvement. Various materials have been mentioned, right? So I would to know how we get these materials.

**K3** We are supposed to buy materials like nails. When we buy the nails, we use them to tack the gauze wire. We also need to buy thimba.

**Facilitator** Anyone with additions on this?

**C1** On the materials needed to tack the gauze wire, we can also use the bicycle spokes. When we do not have money to buy nails, we do use the spokes. We cut them in small pieces and use them for tacking the gauze wire, and it works.

**Facilitator** Thank you. Are there additions of this issue? Alright then, we can continue. I would like to know that apart from the materials that you have mentioned, are there any other materials you think of that can be used in closing small holes? You have mentioned various materials. From pairs of scissors, tape measures, nails to thimba. Are there any other materials you can think of to use for House Improvement, especially in small holes?

**K2** The additional materials for closing small holes are taking the soil and mixing with water to make mud, and close the small holes. These are found right here in the village. As I have said, we just mix soil with water to make mud and close the small holes. This is because there are some small holes which are small for ta brick to fit. So, we just use mud to close them. Those are the additional things.

Reference 7 - 3.03% Coverage

Alright. Are there any other opinions? Alright, let us continue. In your thinking, are the people ready to be buy gauze wire on their own?

**K2** It is difficult for people to manage buying gauze wire on their own because these are very expensive things. The one who can manage… For us, we do not know what will do when this project comes an end. This is because even if you are not old, you cannot manage to buy gauze wire. So, we ours is a request to the project managers that when they get done with their work… I have hope that only the well-to-do people will be able to buy gauze wire. However, we have the orphans, the old persons while others are young but they cannot find money for their households. So the way we are it will be difficult to buy gauze wire on our own. This is the reason we want to encourage the office people (managers of the project) that the mosquito nets…because even the mosquito nets we buy on our own are expensive. We cannot manage buying every time.

**Facilitator** Thank you. Alright. Z1, do you have any additions? Are the people ready to buy gauze wire on their own?

**Z1** Not audible.

**Facilitator** No. I would like to hear your opinion or your thoughts; how you look at it.

**Z1** Not audible.

**Facilitator** Is there anyone with other opinions?

**C2** My opinion is that currently money is scarce. People cannot find money to buy gauze wire. The other thing is a request to the organisation that when you will be leaving, leave a good ground where others will be able to continue from so that malaria should continue reducing here in Chikwawa. Thank you.

Reference 8 - 1.55% Coverage

Thank you. Let us continue. Do the House Improvement works deter you from doing your daily household works?

**K4** The House Improvement work cannot prevent us from doing our daily works because we do not do it every day. It is the kind of work that you can finish in a day. So it cannot make somebody’s works to halt. However, if it was the kind of work that was supposed to be done on daily basis, we would say it is preventing people from doing important works in their life. Thank you very much.

**Facilitator** I can see you B1 that you are smiling.

**B1** I just want to add on what K4 has said. It is true House Improvement works cannot prevent us from doing other works. There is just need for a proper planning of on which day to do what kind of work. So it cannot prevent us from doing our other works.

Reference 9 - 0.47% Coverage

The House Improvement works, as my friend B1 has said, cannot make someone not do other works. This is because you can set a date that on such a day I will tack the gauze wire or close the eaves. So, we can still do the household works. Sure.

<Files\\FA-B HI Commitee members FGD> - § 4 references coded [8.93% Coverage]

Reference 1 - 1.47% Coverage

what were the comments and expectations of people?

**P1:** people were thankful because they are sleeping comfortably than before. People now have forgotten that there is malaria disease.

**P3:** people from our community are asking that if it’s possible, this programe should continue because malaria will be eradicated here in Chikwawa

**P4:** comments from people are in 2 sections. The first one is for afternoon, a house that has gauze wire is looking good and because the house is looking good, it attracts others. At night, the gauze wire is protecting from the malaria. These are comments we hear from the community.

Reference 2 - 4.57% Coverage

okay, what are the materials needed for house improving work?

**P7:** the material needed when inserting the gauze wire are nails, timber, soil and bricks

**Facilitator:** okay, are these materials available in time?

**P7:** yes, they are available in the time that you need to do house improving

**P8:** the materials were not available in time because sometimes the bricks were not readily available. We had to search for the bricks and carry them in the sack then we start looking for water, so the materials were not available at one place

**P2:** just add, when we arrive at the household and see that the house has got open eaves, we were asking the place where they burned their bricks, then we were going there to pick the broken bricks and come back. As we go to fetch water, others were doing the measurements. After closing the open eaves, we were inserting the gauze wire on the same day

**Facilitator:** okay let’s proceed. If the materials were not available in time, what can be done for the material to be available in time?

**P4:** materials were not available in time but for them to be available, we the committee members should have our own materials without depending the household materials because the materials are in different categories like hummer, a panga knife, hoe, wheel barrow, shovel and tape. Some of the materials were provided by the office but we were depending on the household on some of the households like wheel barrow, shovel. It was difficult because we were using our hands such that the work was taking us too long. All these materials, we should have them as a committee

**P3:** if someone at the household says he/she has nowhere to take the bricks, we were referring them where they burned their bricks to collect the broken bricks. If he/she says that he/she cannot afford, we were discussing to do the work. If time is not allowing us that particular day, we were shifting it to the other day.

Reference 3 - 0.82% Coverage

apart from these materials, are there any other material that you think can be used for closing of open eaves?

**P3:** we can do with only mad, not the bricks if the hole is too small

**P4:** if the house was built using unburnt bricks, we could look for a builder who has the material and then we borrow. Some of the committee members are builders

Reference 4 - 2.08% Coverage

Was the house improving work preventing you from doing other health task?

**P6:** It was preventing us but still, as the volunteers we were committed to do because the work came in our village. We were doing our work in the morning, in the afternoon we were doing the other task, it was not painful

**Facilitator:** what tasks were suffered as a results of your house improving membership?

**P6:** there are lots of them but we just skipping others like farming. We were trying to balance them up in the way that morning, we were going to do farming and afternoon, we were doing the other work

**P4:** that’s true, the work prevented us from doing other task especially for us who work from morning to evening, because we accepted to be the volunteers, we were trying to do it voluntarily. For example, I have left the work and come here to participate in this developmental project

<Files\\FA-C Animators FGD> - § 7 references coded [11.50% Coverage]

Reference 1 - 0.40% Coverage

P4, do you have anything to add on this? What are the things that people do when closing open eaves?

P4: repeating what P6 just said, yes, we look for bricks. Then we make mud for laying the bricks. When they’re small spaces, we get mud and use it to close the spaces.

Reference 2 - 2.94% Coverage

alright. Moving on with the topic about house improvement, could you tell me what are the necessary materials when you are improving the house?

P1: one necessary step when improving the house is the house owner looking for bricks. When bricks have been organized, then you start closing open eaves, making sure the walls are in contact with the roof. When closing open eaves, you will need mud. For the windows, you will need nails for nailing the gauze wire. You will also need some baboons or reeds for sticking the gauze wire to the window flame so that it doesn’t come off when there’s strong wind.

P2: just adding on what P1 mentioned, the other thing is we provide advice to the house owner depending on the condition of the house. Especially a grass-roofed house, you can close open eaves but if the roof is broken we need to advise them to also work on the roof, so that both open eaves and the roof are properly fixed so there’s no entry of mosquitoes into the house.

P3: my friends have already discussed most of the things, but I wanted to talk more about materials that came from the project. Every committee was supplied with one hammer, a pair of scissors, one [measurement] tape. Now, we talked about some people not being able to improve their houses as required. For such households committee members are supposed to go and help. But with only a hammer, a pair of scissors, a tape, it was difficult for a committee of 10 members to work properly, all the members plus the house owner depending on the same tools.

P6: my friends have mentioned it all, but regarding materials needed when improving a house, first, there was need for a hoe, water for soaking the mud. There was also need for ‘mpeni womangira’ if you were closing open eaves using bricks. If you couldn’t find mpeni womangira, you would use a phanga knife. We also needed nails, a tape for measuring the exact length of the gauze wire before cutting to avoid misuse of the wire, and a hammer.

Reference 3 - 2.35% Coverage

you mentioned a number of tools, including a hoe, mipeni or zisenga, bricks, mud, nails, timber, gauze wire. I wanted to know are these tools readily available?

P2: um, these tools are not readily available. Why? Because the gauze wire has to come from town with our project officers. A hoe or a phanga knife or mpeni womangira is readily available because people have them in their homes, and so is the tape or the hammer because committee members keep them. Delays in receiving the gauze wire sometimes delay our work.

F: P2, if you could expand on that, you said gauze wire is not readily available. What do you think could be done to ensure the gauze wire is readily available?

P2: to ensure these things are readily available, we have offices at the Epicentre here as well as at Chapananga. If these offices could be supplied with the materials, it would be easier for us to come and collect them from here and distribute them to people.

P3: what’s required is to report immediately if the gauze wire stock is finishing. That would ensure readily availability of the wire because when one supply is about to finish another arrives.

P6: responding to the first and second questions, I think this work is done in phases. We can’t supply gauze wire when open eaves have not been closed. Every part of the work has its own phase. If we are going mold bricks, it means what will be supplied are materials for brick making. Similarly, if we are going to close open eaves, what will be supplied are materials for that work, and so if you are going to put gauze wire on the windows.

Reference 4 - 1.26% Coverage

Alright. A number of materials for house improvement have been discussed here. How do people in the village find these materials?

P6: most of us in the village are farmers, and the materials we use are those that we can find in our homes such as hoes. For mpeni womangira nyumba, we borrow them from builders. But for other materials that require purchasing such as scissors, hammers, measurement tapes and gauze wire, the project would support us with them. For nails, some of us would buy them from stores, others would make them out of spokes.

P3: for nails, people had to buy them from stores, those without money would make them out of bicycle spokes. For other tools such as hoes or phanga knives, we already had them in our homes so we just use them. Some of the tools came from the project such as scissors, measurement tapes and hammers.

Reference 5 - 1.95% Coverage

I also want to know. Are people ready to look for the gauze wire on their own? I know that the gauze wire is provided by the project. But I want to know if people are ready to buy the gauze on their own?

P4: we can’t buy gauze wire on our own because we can’t raise money.

P1: if the project stopped providing free gauze wire, we can buy it as long as it’s available in our village stores and at a lower price, because having it on the windows is protecting our lives from illness.

P3: I have been talking to some people and they have been telling me that “We are ready [to buy on our own] even if the project stops providing gauze wire.” “Why?” “Because the money I spend on managing malaria-caused illness annually is more than I can spend on buying gauze wire, and the money that I save by preventing malaria illness I can use it to buy a goat.” So people are ready to buy because they realize the benefit of the gauze wire. And I know someone who went and bought gauze wire on their own before the project started providing it. So there’s proof that people can buy it.

P5: if the gauze wire was cheap, many people would be able to buy it. But since it’s expensive, maybe if people contribute money and buy as a group. But everyone on their own, especially if they have no means of support, can’t buy it.

Reference 6 - 1.42% Coverage

alright. Does the house improvement work stop you from fulfilling your personal daily activities?

P1: the house improvement work does not stop us from completing our household chores. All the house improvement activities are preplanned, so we know in advance what we are going to be doing on such and such a date or in such and such a month. If you have a calendar of events, it’s easier to accomplish your daily activities.

P2: in our village, this work has been going on very well. We have a schedule of our activities. We inform committee members, chiefs and animators that our work plans should start at 1pm, because the morning is for doing our things.

P3: this work does not interrupt our household activities. Why? Because we have work plans. If we agree to meet on Wednesdays, then I ensure that I all my household chores are completed before that day. And if we meet just once in a week, it means I have more days for doing household chores.

Reference 7 - 1.18% Coverage

okay. The other thing I wanted to know is would people in the village be willing to pay for an improved house for malaria prevention?

P5: yes it’s possible, they can pay for the gauze wire because they are safeguarding their life from malaria.

P2: people can personally pay for gauze wire, but the problem would be for those who have no means of support – most of them will die because they can’t afford it. They are already struggling to buy maize from ADMARC, so how much harder will it be for them to buy gauze wire? It’s difficult. If the price was fair, or if they were forced to buy, they might buy.

P3: people would be willing. Why? Because of the benefits they are seeing. Malaria is a very serious condition, people would be willing to pay so that they are protected from malaria.

<Files\\FA-C community participants FGD 2> - § 10 references coded [20.91% Coverage]

Reference 1 - 1.78% Coverage

In House Improvement work, we take soil and mix with water and use it to close all the spaces through which mosquitoes could enter the house.

**Facilitator** Alright. That is P2’s opinion. Let us hear other opinions. P8?

**P8** The work we do during House Improvement includes drawing water and putting where we want to dig the soil. After digging the soil, we mix it with water. We then carry it into the house. We then start closing the spaces.

**Facilitator** Thank you.

**P6** The House Improvement work depends on the kind of space. If the space is big, we take bricks to close the spaces. If there is a small space, we use mud. When we do that we are done.

**Facilitator** Alright. Let us perhaps finish with P7.

**P7** The mud we use for House Improvement has to be sticky. If we take sandy soil, it does not stick to the wall…

Reference 2 - 5.18% Coverage

need mud. The improved house also looks good.

**Facilitator** Alright. The main thing I would like to know is; when people are doing this work, is the work tedious or just fine? How do we look at it?

**P6** Some people would need someone to do the work for them, for example unmarried women. These ones need to be paid.

**Facilitator** Alright. P7?

**P7** There is a lot of work. The wife can be going to draw water while you are making mud and carrying bricks and the mud to the person closing the eaves. Then you can be told to take something and put somewhere. It is a lot of work to do the whole house. When you are tired…

**Facilitator** Alright, that is P7’s opinion. Let us hear from P4.

**P4** It is tedious work especially to the committee members in households where there are elderly people or unmarried women. For example, the elderly cannot find money to get a builder to do the work for them. Instead, the committee members do the work. Even when the roof has been blown off by the wind or during the rainy season, it is the committee members that do the work. That is where it might seem to be tedious. But it is not very tedious if someone can understand it.

**Facilitator** P4, the way you are explaining, is the work tedious to you as an individual too or only to the committee members?

**P4** It is not a very tedious work to me. It can take me two days to do my house.

**Facilitator** Alright. I was a hand here. P1?

**P1** No.

**Facilitator** You did not raise up your hand? Alright. You have said this work is tedious, as P7 said. What can happen to make this work easy?

**P7** I think everyone should be closing the eaves and spaces the time they are constructing the house, whether it is iron-roofed or grass-thatched. They should be getting done with it the time they are constructing the house.

**Facilitator** Alright. That’s P7’s idea. Anyone with additions.

**P4** For us with grass-thatched houses, let us make sure the roof is not leaky in the walls during the rainy season. This will make the House Improvement work less difficult. However, it the roof is leaking along the wall, there will be a lot of work.

**Facilitator** Alright.

**P1** I think there is need for the owner of the house and those bringing the gauze wire to help each other with the work. When one is getting the bricks, another one should be getting the mud and the other tacking the gauze wire. That way, it will be easier.

Reference 3 - 4.25% Coverage

Alright. I think we should continue our discussions. I would like to know from you. What are the materials that are required during House Improvement? Would you mention all the materials that are required when we are doing House Improvement? Let us start with P8 then P2. Mention the materials required during House Improvement.

**P8** The materials required are; hoe, builder’s knife, level and measuring tape.

**Facilitator** Alright. P8 has told us some of the materials. ***(Materials mentioned again).*** P2, can you add other materials that are required?

**P2** My additions are; getting the wet soil and close the spaces. This is because you close the spaces after you had already built your house and you did not know what would come. So, you close where there are spaces using your hands. This does not require a builder’s knife.

**Facilitator** Alright. P2 says you need soil. Any other materials that we can add, P7?

**P7** The other materials we can add are; shovel, wheelbarrow and builder’s knife.

**Facilitator** Alright. Here, I would like to learn from you the materials that are used in the village. ***(Question repeated for clarity)***. Let us start with P4, then P1.

**P4** The materials that are required at first are; a hammer, nails or wires cut into small pieces, gauze wire, timber used for tacking the gauze wire, hoe, water, bricks as well as builder’s knife, which has been mentioned, to make it look good, and finally ladder to use for closing the eaves.

**Facilitator** Alright, that is P4. P1, do you have additions?

**P1** That is all.

**Facilitator** Are there any additions on the materials? Alright. Some materials have been mentioned here ***(Materials mentioned again)***. So, I would like to hear what you have been seeing in the village. Are the materials you have mention available in time they are needed? I would like to learn from you. Let us start with P5.

**P5** We forgot one material. A pair of scissors and gloves are required for cutting the gauze wire.

Reference 4 - 2.59% Coverage

Alright. That’s P4’s opinion. Let us hear other opinions. How do you get these materials in the village? One way is lending each other. Another one is getting from the committee members. How do you get other materials, P8?

**P8** When there is nowhere to borrow from, we do buy some of the materials if we have money. For example, hoe, shovel, measuring tape and builder’s knife. We buy when there is nowhere to borrow from.

**Facilitator** Alright. Are there any additions? Anything additional to this issue? Alright, let us continue. Apart these materials, are there any other materials you can mention that can be used for closing small spaces? We start with P4, then P7.

**P4** When there are small spaces, we can use builder’s knife and thick mud.

**Facilitator** Alright. You have talked about builder’s knife and thick mud for closing small spaces. Others? P7?

**P7** The materials that…like a pointed piece, piece of knife or else buying a small hoe to close the small spaces.

**Facilitator** Alright. P1?

**P1** If it is grass house, we plaster the house properly with soil to close every small hole. For our friends with brick houses, they buy cement and plaster the walls to close the small holes.

Reference 5 - 1.12% Coverage

Alright. Let us finalise on this. I would like to hear your opinion. In your own thinking, are the people in the village ready to be buying gauze wire on their own, if Majete Protect was to end today? I would like hear your opinions. Let us start with P1.

**P1** People are not ready to be buying own their own. This because when you are going to tack the gauze wire in their houses, they say they do not have nails. So, if they do not have nails, would they manage to buy gauze wire? So, I think they cannot manage.

Reference 6 - 1.06% Coverage

Alright. We have heard P1’s opinion. Let us have other opinions. Are the people ready? P3 has talked a lot. P2, are the people ready in your village? Are the people ready to buy gauze wire on their own?

**P2** People are not ready to buy gauze wire on their own because money in our area is a scarce. So, they cannot manage. It is the same what P1 has said that if people complain that they cannot manage to get nails to tack the gauze wire, it is difficult for them to buy a gauze wire.

Reference 7 - 0.25% Coverage

There are unmarried women and the elderly who do not manage to buy even a hoe. So, they cannot manage to buy a hoe.

Reference 8 - 0.42% Coverage

It is difficult here in our village because we lack a lot of things. So, buying gauze wire, we cannot manage. We do not even know how many metres are there per roll. So, it is a lot of money.

Reference 9 - 2.60% Coverage

Alright, we have heard your idea. Let us perhaps continue. Do the House Improvement activities prevent you doing your daily household works? Let’s start with P5?

**P5** They cannot prevent us from doing daily works because we are protecting our lives from mosquitoes.

**Facilitator** Alright. P7?

**P7** When doing this work, we choose a specific day for it. For example, Monday I have to do the House Improvement work and from Tuesday onwards, I have to do other works like farming, et cetra.

**Facilitator** Alright. So, the way you have explained, the the question is; do these works prevent you from doing your everyday works?

**P7** No. They cannot prevent us from doing everyday work.

**Facilitator** Alright. That’s P7’s opinion. P8, let us hear your opinion. ***(Question repeated)***.

**P8** Yes, they do. This is because when you are doing House Improvement work, you do not do the daily household works.

**Facilitator** Alright, that is P8’s idea. Let us hear from P1.

**P1** My opinion is that this work does not prevent us from doing daily household works. This is because we can do it today and work as group with the committee members. It may take two days. Then, we will do the household works on the other days.

Reference 10 - 1.66% Coverage

Alright. Perhaps, let us finalise this section with the following question; can the people in the village be willing to pay so that they should have improved houses to prevent malaria? ***(Question repeated)***. I would like to hear your opinions. Let us start with P6.

**P6** Yes, it is possible to pay money because they have seen its importance that it prevents everyone from malaria. So, when you are preventing malaria, you can pay money because it is life.

**Facilitator** Alright. That is P6’s opinion. Let us have other opinions. P1?

**P1** They can be willing because of how it’s done, how it looks as well as differentiating it with where they are staying so that they can reduce malaria; that when they move to the improved house, they will perhaps reduce malaria.

<Files\\FA-C HI Committee members FGD> - § 3 references coded [9.36% Coverage]

Reference 1 - 1.80% Coverage

Let’s here from P8. What are the activities that you do when doing house improving?

**P8:** First of all, when we see that a house was not properly closed, we close those gaps to prevent mosquito from getting in the house. If there are gaps on the doors, we close those gaps as well. We then finish with the windows where we insert the gauze wire

**P6:** what happens on HI, if the house shows small gaps, we close with mud so that there should be no gap, we have therefore prevented malaria. Sometimes mosquito can get in the house when we have left the door opened. After that, mosquito get in the house and gets a net. That’s how we prevent malaria

Reference 2 - 2.84% Coverage

there should be at least 3 hummers at the team then we can share and the work can be done fast.

**Facilitator:** you have mentioned a hummer, I wanted you to mention other materials that are needed.

**P1:** okay, gauze wires, hummer, scissors and tape, these are materials that are needed as well as gumboots so that we can be safe when we are traveling

**P8:** materials are gauze wires, scissors, tape, gumboots, uniform is finished, we need the bicycle to carry the gauze wires rods, I think these are what I can remember so far

**P7:** the materials which they have mentioned, I am adding hoe and shovel because mud needs a hoe and the shovel

**P3:** we need hoe and the panga and the level

**P2:** hats that should prevent us from the sun

**P3:** A wheel barrow is needed because others refuse to have holes at their household, so they tell us to bring the soil from somewhere, so we need the wheelbarrow

**P2:** what makes this job to be difficult is how to get nails. Most of the times we use the bicycle wire instead of the nails

Reference 3 - 4.73% Coverage

I would like to hear from you, are these materials available on time?

**P7:** these materials are not available, we get but few of them. It’s difficult to force ourselves to have them

**Facilitator:** I want you to clarify on which materials are not available on time and what can happen for these materials to be found.

**P7:** we miss materials like wheelbarrow, shovel, hummer, nails and the carrier that we can use to carry the materials when going to do the work. We need these materials as group, the secretary could be the custodian of these material and on the working day, we can just go and take them

**Facilitator:** what can be done for these materials to be available?

**P7:** we ask the organization that helps us in this program to act on this so that we can have these materials

**P8:** there are some materials that can be found at the house where we want to work, for example a hoe, the owner can borrow us. Some materials cannot found in the communities, for such materials, we ask the organization to help

**Facilitator:** okay lets proceed. How do people in the community outsource the materials?

**P3:** We get hoe from the owner and if he/she refuses, we cannot miss the hoe in the community. We need bricks and if the owner has no bricks, we do ourselves. The building materials like a building knife, most of the times we use a panga knife. We get soil from far and instead of wheelbarrow, we just use a sack bag. We therefore use the simple materials as long we do the work

**P6:** other materials like nails and the tape measure ,we can get them from the shops. Other materials we ask your help

**P7:** the materials that we use, we bought them. We bought what we could afford. Some we could not afford

<Files\\GVH KAMOGA> - § 7 references coded [18.85% Coverage]

Reference 1 - 2.40% Coverage

do you into the activities involved in improving the house

Respondent: **aaa it is very big job aaaa but it is not like that big. Aaaaa so when building the house there are so many things involved. So we try to make what eee to make it possible aaa we try ee to make it possible eee**

Reference 2 - 3.05% Coverage

what resources are needed for house improvement? Mention the resources

Respondent: uhumm resources needed, here we use the same soil since it is difficult to find money for buying cement and others. We take the same soil, that’s what we use. Since the issue of money is scarce here in the village, we cannot buy cement for improving the houses. We use soil only

Reference 3 - 2.22% Coverage

a house needs doors, maybe nails if fixing iron sheets, wire when building the wall of the house. These are needed when improving a house

Interviewer: these resources, are they readily available?

Respondent: we struggle to find them since this is the village

Reference 4 - 3.29% Coverage

the resources, some buy, others use grass, we cut some small trees and fix the roof with bamboos although now bamboos are not readily available, and we like using some small trees. Others if they have money they buy iron sheets and fix them, others fail to buy iron sheets, they just mould bricks, burn them and build a house and take some small trees and fix the roof and thatch with grasses

Reference 5 - 1.40% Coverage

other resources, if there would have been cement, it would have been better because the house is strong when closing gaps with cement unlike if it is done with soil

Reference 6 - 2.12% Coverage

they cannot afford on their own because it needs money. To find money here, we depend on pigeon peas and this crop sometime has not been doing fine. So I cannot guarantee that everyone can find money and buy for their selves. aaaahh they cannot manage

Reference 7 - 4.37% Coverage

alright. Do house improving activities affect your day to day routine work activities?

Respondent: aaahh it can affects us, house improving activities affect because you are busy building the house

Interviewer: what kinds of work are affected or you fail to fulfil when you are engaged in house improving activities?

Respondent: farming activities. Sometimes we are found at our gardens farming; sometimes we do house improving activities, sometimes going to the gardens, so you involved with those kinds of work

<Files\\GVH MAKANDE(1)> - § 9 references coded [20.13% Coverage]

Reference 1 - 3.64% Coverage

What are the comments and expectation made by people about this intervention?

Respondent: Comments are like just encouraging people to be going forward while the malaria group people are adding other villages which they were outside this project (house improvement intervention) I just want to encourage these people to move forward on this intervention to prevent malaria.

Reference 2 - 2.54% Coverage

Respondent: aaaa there are the same methods mmmm because the brick house is supposed to have closed eaves and every hole on the walls has to be closed with mud. And if the house has iron sheets make sure has no space on it. These are the methods which we follow.

Reference 3 - 4.10% Coverage

thank you. We are proceeding to another section of questions. What are the materials required in improving the house? Hmmm am saying materials needed for house improvement. Mention them

Respondent: Closing eaves involves building knife, bricks and mud, for the window it involves gauze wire, nails and bamboo.

Interviewer: Are these resources available in time?

Respondent: Yes they always been available when needed

Reference 4 - 1.34% Coverage

Respondent: Gauze wire is been sponsored by malaria project but these timbers are locally available and for the nails we buy at the shops.

Reference 5 - 0.37% Coverage

Respondent: Knife and mud for building

Reference 6 - 1.89% Coverage

Alright chief I want to know, do you that people ready to buy these materials on their own?

Respondent: hmmm aaaa hmm for that I cannot have the best idea because for the gauze wire we receive

Reference 7 - 2.13% Coverage

Alright. Do these activities make you fail to do your everyday activities? Let me ask again do you think these activities involved in house improvement make you fail to do your everyday activities?

Respondent: hmm no

Reference 8 - 2.82% Coverage

Alright what duties are forgone by engaging in house improvement activities?

Respondent: [long pause] like closing the house is not that difficult but when you want building the house it is busy work since it involves a lot of activities but just improving the house is not that busy work.

Reference 9 - 1.31% Coverage

Are the people in the community willing to pay for improved quality housing for preventing malaria?

Respondent: Yes it can be possible

<Files\\HSA Bwalo Transcript KII> - § 7 references coded [16.86% Coverage]

Reference 1 - 2.49% Coverage

**Interviewer**: How can you explain the workload/activities involved in the implementation of HI?

**Respondent**: I feel like the activities involved are alright because the health animators are always making supervisions in the villages looking at houses that have been improved and those that have not. They check if those that were provided with wire gauze have placed it in the windows if not they tell them that it will be taken and be given to others that are serious with using the wire gauze.

Reference 2 - 3.52% Coverage

How can you describe the workload involved in HI activities?

**Respondent**: At first when the project was starting it was agreed that some houses will be selected as demonstration houses so that people would go there to benchmark on how they have been improved. So like the first demonstration houses were improved by the committee, they selected 2 houses per village. After these were improved they called people in the community to come and learn how the houses have been improved. It was stipulated on this encounter that each and every family in the village should come to learn and benchmark. So every family in the village would do this task on their own without the involvement of the committee.

Reference 3 - 1.01% Coverage

The way I see the work itself, it is alright because a majority of the people in the village did house improvement. If it was a demanding task then we could have less people doing house improvement.

Reference 4 - 1.21% Coverage

When someone is doing house improvement firstly you need bricks, also sand for them to use, when it comes to the windows we need nails for placing the wire gauze on the window. Also some people use small timber when placing the wire gauze.

Reference 5 - 3.24% Coverage

So on this issue of resources required, are these resources readily available?

**Respondent**: Yes, a lot. Because when it comes to bricks people mold and burn bricks on their own. They don’t need to buy. Some even borrow from their friends. If someone does not have we encourage them to mold and burn so that they do HI. Even the sand is readily found. The nails are not that expensive as well, people normally buy the small nails which are cheap. So we just encourage them to find these equipment that they are cheap and they should find them, even for some people they even use sticks and just sharpen them instead of nails. They even look nice.

Reference 6 - 1.38% Coverage

In your opinion are people ready to buy wire gauze on their own?

**Respondent**: I don’t think so. Most people complain as you know how life in the villages is, many people are not working so if we tell them to buy wire gauze it would be a difficult thing, they can’t manage.

Reference 7 - 4.00% Coverage

What duties/chores are foregone by engaging in HI activities?

**Respondent**: No. Because we tell them that when they are doing HI they should not take it as a demanding task, for instance we can tell them that in the morning that they should do their personal tasks but in the evening most of them are free so that’s when encourage them to do the exercise of house improvement. For instance closure of eaves is not a task like you are building the whole house, it’s very little task maybe for even an hour one can be done. So we encourage them that in the morning they can go and do their daily chores but in the afternoon hours when they are free they can talk to their spouse that they should do house improvement. The ladies would help in drawing water whilst the men would be doing the building.

<Files\\HSA Liwonde- Kapichira Health Center KII> - § 9 references coded [23.07% Coverage]

Reference 1 - 4.77% Coverage

Alright. Let us perhaps go to another section about the work. I would like to know how you look at the work that is involved in House Improvement.

**HSA** As I have pointed out earlier on, the work that takes long during House Improvement is closing of eaves because they need to find bricks. Otherwise, there is no problem with the issue of gauze wire.

**Facilitator** Alright. Perhaps in continuing with our chat, I would like to know if the work is either tedious or not.

**HSA** It is simple work. It does not take a lot of time especially when all the required materials are available. It is so easily done that you can work on some houses in a day.

Reference 2 - 1.67% Coverage

when doing House Improvement, there is need for the gauze wire and snaps, which the Majete Malaria Project team already provided us with, nails or small pieces of wire, and small pieces of wood to use in tacking the gauze wire.

Reference 3 - 1.16% Coverage

Some of these materials are available in time while others such as bricks are not. However, materials like gauze wire and nails are made available in time.

Reference 4 - 3.47% Coverage

As I pointed out, currently people are closing the eaves of the houses they are constructing. This means they understood the message that when constructing the houses, they having to be closing the eaves right away while they still have the bricks. This is different from what was happening previously when people thought it was not important to close the eaves when constructing houses. Nowadays, people are closing the eaves right when they are constructing the houses.

Reference 5 - 2.27% Coverage

On the part of nails, people try their best to source some money to buy them. For those who constructed their houses some time back and left the eaves open, they borrow bricks from their friends who have them and close the eaves. For the gauze wire, it is distributed by the Majete Malaria Project committee.

Reference 6 - 0.93% Coverage

Mainly, small spaces other than windows which are in the walls are closed using mud. We cannot tell to be closing using cloth.

Reference 7 - 4.79% Coverage

Alright. In your opinion, are the people in your village ready to buy gauze wire on their own if Majete Malaria Project was to end?

**HSA** If the Majete Malaria Project was to phase out and they have stopped distributing the gauze wire, I think it would be difficult for people to buy because many people would not manage. However, some people would manage to buy. The people, however, know the importance of the gauze wire. This is the reason people are currently coming earlier to report to the committee about damaged gauze wire which needs to be replaced. This means they know its importance. The only problem would come due to financial problems.

Reference 8 - 2.47% Coverage

Alright. Do the House Improvement works prevent you from doing other usual daily works?

**HSA (*Laughs*)**. No. This program does not prevent me from doing other works. In fact, it makes the work interesting because it is part of the job that I, as a Health Surveillance Assistant, need to do to prevent the spread of malaria in the village.

Reference 9 - 1.54% Coverage

Paying so that they stay in properly improved houses so that they do not catch malaria.

**HSA** Renting?

**Facilitator** Yes. Whether renting or building a house that is properly improved.

**HSA** Yes. That is possible

<Files\\KII Chief Chambo FA-C> - § 12 references coded [25.61% Coverage]

Reference 1 - 2.07% Coverage

What are the work that people do when improving the houses

Respondent : People do works like closing the eaves with bricks, measuring the window with the wire and locking and searching for the cracks in rooms and closing with the mud as the owner of the house

Reference 2 - 3.70% Coverage

All right, what are the comments and expectations generally people have towards this house improvement intervention?

Respondent : these comments and works

Interviewer : mhmm

Respondent : people are recommending the malaria project for the great job done, which has reduced malaria in our area and they are encouraging that this work should be sustainable, we are independent to do this job even after malaria project leaves up until malaria is greatly reduced

Reference 3 - 0.46% Coverage

it was a tough job at first but now it is a simple work

Reference 4 - 4.41% Coverage

What is it involved in improving the houses

Respondent : the work starts with closing the eaves, after that we look for nails and wire, which was been given to the leaders; they come to measure our house. then they come to place them or we also have a responsibility to place them for ourselves and then we make sure that the eaves and windows are closed for mosquito to pass through. We then enter the house to look for the openings or cracks to close. When we see that there are some open spaces showing off light, we take the mud to cover such places

Reference 5 - 1.30% Coverage

All right, is the work of improving the house good or tiresome?

Respondent : it is not tiresome because the work is been done once in a time; we do not do it dairy

Reference 6 - 2.09% Coverage

is through meeting, by calling people and explaining to them and this work will be easier because everyone will understand and welcome the thing rather than hearing it from somewhere, the person understands when called and explaining in details about the news.

Reference 7 - 1.82% Coverage

tools like bricks and soil are easy to find but maybe wire, we may find it since there are many houses, and it becomes finished. The wire gauze is been received as soon as we submit the report to Majete malaria project head office

Reference 8 - 2.88% Coverage

what should been done so that the materials should be found in time if they are not available in time?

Respondent : There should be timely reporting when the wire is about to be finished

Interviewer : how do you find the materials for improving the houses?

Respondent : Mesh, measurement tool, scissors and humour are been found through malaria project

Reference 9 - 2.31% Coverage

Are there other things that you think can be used to close the small openings and the gaps

Respondent : Aaaa Nothing, because these are the most important things that are been needed for the houses like these because we cannot talk about cement, it cannot be possible, we cannot afford

Reference 10 - 1.92% Coverage

In your opinion, do you think people are prepared to buy wire gauze on their own?

Respondent : They are prepared if they can be cooperative because they told us that wire gauze is cheap when we contribute the money as a group, we can manage

Reference 11 - 1.23% Coverage

All right, were you unable to do dairy chores due to your engagement to the works of improving the houses

Respondent : No, they were not restraining us

Reference 12 - 1.45% Coverage

Are the people in the village willing to pay money to have quality houses for preventing malaria?

Respondent : some of them can afford but some cannot due to financial challenges

<Files\\KII Chief Goliati> - § 9 references coded [9.34% Coverage]

Reference 1 - 1.97% Coverage

What are other alternative methods which can be used for house improvement

Respondent : ohoo, the only method which we recommend is the use of wire gauze because using the mud as we were doing in the past,

Interviewer : mhmm

Respondent : Mosquito was still entering the houses with closed window grasses

Interviewer : mhmm

Respondent : the coming of the wire gauze

Interviewer : mmmm

Respondent : we see that it is better than using mud

Reference 2 - 0.88% Coverage

aaah, I see that the house improvement work is not tiresome, it is a good work because since morning up to this time we could have covered four houses. At first, I was moving around with the committee

Reference 3 - 1.15% Coverage

aaaah, the recommended materials, some were using the bamboo to tighten the wire gauze instead of nails

Interviewer : mhmm

Respondent : and using them on traditional houses

Interviewer : mhmm

Respondent : so I stopped that after I saw that it is not helping

Reference 4 - 1.06% Coverage

mhmm

Respondent : eeheee, to use when closing such places, if the owner of the house decided to construct the whole house with cement

Interviewer : mmm

Respondent : He also use cement to close the eaves, those are the important materials

Reference 5 - 0.64% Coverage

These materials are available when the person is ready to do the work

Interviewer : mhmm

Respondent : yeah, that when he gathers the materials

Reference 6 - 0.67% Coverage

eeeh, what should be done is that when the person has been told, and he knows that his windows are supposed to be closed so he prepares the bamboo early

Reference 7 - 0.28% Coverage

When the house has smaller openings, we just close with the mud

Reference 8 - 1.17% Coverage

Alright, I would also like to know, are the house improvement activities delay you from doing your dairy activities?

Respondent : No, they don’t delay us from doing our dairy activities, our dairy activities cannot be left unattended to due to house improvement

Reference 9 - 1.53% Coverage

Are people in the community willing to pay for the quality improved houses to prevent malaria?

Respondent : Ahhh, it may be possible that…. There was a program to discuss if it is possible to have a bank so that we will be able to buy wire on our own after we have been left. That is when other people said they cannot afford due to their poverty

<Files\\KII Kalinjala> - § 6 references coded [9.06% Coverage]

Reference 1 - 1.78% Coverage

what activities are involved in improving the house?

Respondent: they use mud to close the holes and put the gauze wire over the window.

Reference 2 - 0.84% Coverage

eee for closing the spaces we use hoes, shovels water and bucket

Reference 3 - 0.71% Coverage

where do you find these materials?

Respondent: we buy

Reference 4 - 0.58% Coverage

Respondent: eee knife for building the house.

Reference 5 - 2.02% Coverage

okay. Want to know, do you think people are willing to buy gauze wire on their own

Respondent: yes but with our season here it is difficult to find money

Reference 6 - 3.13% Coverage

what type of work.., okay are people in the community willing to pay for improved quality housing for preventing malaria

Respondent: they may accept

Interviewer: please explain

Respondent: if they may wish to sleep in the improved houses

<Files\\KII MACHEKERO(1)> - § 7 references coded [7.42% Coverage]

Reference 1 - 1.31% Coverage

mm

Respondent: activities that they do in relation to gauze wire when the committee has given to you, the wire is measured depending on the sizes of the windows and put them on the windows.

Interviewer: alright.

Respondent: in case we don’t have nails for pasting the wire on the window, we use sharpened bamboo to fix the wire

Reference 2 - 1.06% Coverage

aaa alright. Maybe some houses have no glasses while others have, so those without glasses we follow the same procedure. Maybe other houses don’t have windows; we also follow the same procedure so that the bamboo should act like a window to avoid the wire from falling.

Reference 3 - 0.74% Coverage

this work, I can say, to me as a chief I don’t think it is tiresome because am taking care of my life. If one has given you maize, you cannot expect the same person to go maize mill for you

Reference 4 - 0.70% Coverage

the resources availability during the work?

Interviewer: yes

Respondent: for the wire, its readily available. If they give us, there is a fixed time when it might decompose

Reference 5 - 2.23% Coverage

so how do you find the resources used for house improvement?

Respondent: resources for improving the house?

Interviewer: mmm

Respondent: for the fire when fixing it, aahhhh when those who were giving the gauze wire, they also gave us a hammer, machine for cutting the wire, tape measurer. If these resources haven’t reached us yet, we use stones, our knife and a string to measure our window and cut the wire using the measurements we have made using a scissor bought from the grocery. We take a knife and sharpen some sticks and fix the wire using the stone.

Reference 6 - 0.88% Coverage

buying wire on their own, that depend on money because even as a chief you may say I will buy but maybe the people may not afford and may say we have given them a burden and wont manage or maybe we will just manage a net..

Reference 7 - 0.50% Coverage

can people accept, in the villages, to pay money with the aim of living in the improved houses so that they do not get malaria?

<Files\\KII MDZACHI(1)> - § 1 reference coded [1.32% Coverage]

Reference 1 - 1.32% Coverage

Respondent: other resources are available. Resources like sharpened stick, if there were bamboos, we would have used bamboos but because we don’t have, we use sticks to pin the wire

Interviewer: mmm

Respondent: after that they fix the wire using the stick

**Description:** Describes the participants' views and opinions on program quality, program delivery standards, and adherence to HI implementation standards.

<Files\\Chief Kuzambo - KII> - § 2 references coded [2.53% Coverage]

Reference 1 - 1.19% Coverage

# Chief: When a house has been improved to standard it shows, and majorly, there’s a decrease of malaria episodes in that housesold.

Reference 2 - 1.34% Coverage

# Chief: Yes, people are following the standard way of house improvement, but its not all of them. Some do what they have been told while others don’t.

<Files\\Chief Maganga 2 Chichewa KII> - § 3 references coded [5.16% Coverage]

Reference 1 - 2.64% Coverage

# Respondent: Some of the house improvements are not to standard, because sometimes the owner of the house is the one who does the work, so some do not have husbands and live alone with other women while others are elderly. So for them to do the work by themselves produces sub-standard work.

Reference 2 - 1.12% Coverage

# When a house has been improved to standard it shows, and majorly, there’s a decrease of malaria episodes in that household.

Reference 3 - 1.40% Coverage

# Respondent: Yes, people are following the standard way of house improvement, but its not all of them. Some do what they have been told while others don’t.

<Files\\Community participant Chaziya IDI> - § 1 reference coded [6.60% Coverage]

Reference 1 - 6.60% Coverage

**INTERVIEWER:** do you think all houses in your area do house improvement in standard?

**PARTICIPANT:** yes, they are been done to the standard

**INTERVIEWER:** which standard do you check if the house has been done accordingly?

**PARTICIPANT:** for the gauze wire they make more measurement than of the measurement of the window so that the wire should be a bit free with the window frame

**INTERVIEWER:** want to know from you do people in your community follow/stick to these standards?

**PARTICIPANT:** yes they follow

**INTERVIEWER:** still I want to know, do you think it is possible to follow these standards?

**PARTICIPANT:** yes it is possible when these standards are been followed; the diseases here at Chanzia village will be reduced only if we follow these standards

<Files\\Community participant chaziya-chapananga IDI> - § 2 references coded [6.00% Coverage]

Reference 1 - 2.51% Coverage

**INTERVIEWER:** I want to learn from you do you think all houses are done to the standard?

**PARTICIPANT:** yes

**INTERVIEWER:** what standards do you see if the house has done rightly?

**PARTICIPANT:** first the put wire and put it in order, and mosquito nets inside

Reference 2 - 3.50% Coverage

**INTERVIEWER:** do people of your community stick to the standard of house improvement?

**PARTICIPANT:** some people don’t follow some follow

**INTERVIEWER:** do you think is possible to stick to these standards? What can make them to follow this?

**PARTICIPANT:** yes is possible because we have committee who makes sure that the houses have been done in accordance

<Files\\Community participant Garonga IDI> - § 1 reference coded [2.67% Coverage]

Reference 1 - 2.67% Coverage

alright. Do you think all house in your area do house improvement of standards?

**PARTICIPANT:** every house that has gauze wire it is properly improved house

**INTERVIEWER:** what standards do you check if it has been done accordingly?

**PARTICIPANT:** firstly even if we are outside, we check for the gauze wire and then check inside you find there is darkness that is when you know it is properly done

<Files\\Community participant Goliati IDI> - § 2 references coded [3.00% Coverage]

Reference 1 - 1.96% Coverage

**PARTICIPANT:** house are been done in standard manner, once we have closed the eaves we enter in the house to see if there is light (mberewera) so if there is such light we re-do the exercise

Reference 2 - 1.04% Coverage

**INTERVIEWER:** do you think the standards of improving the house can be adhered to?

**PARTICIPANT:** yes

<Files\\Community participant Liwonde IDI> - § 1 reference coded [1.83% Coverage]

Reference 1 - 1.83% Coverage

**INTERVIEWER:** do you think house improvement is done in standard in all houses?

**PARTICIPANT:** yes. For some who understood the advice they did in accordingly

<Files\\Community participant Mtemera IDI> - § 2 references coded [1.35% Coverage]

Reference 1 - 0.72% Coverage

**PARTICIPANT**: because some house after done house improvement standards the rain dilute some mud creating space

Reference 2 - 0.63% Coverage

**PARTICIPANT:** we see at top of the house, closing the space, putting gauze wire on the window

<Files\\FA-A Animators FGD> - § 1 reference coded [2.58% Coverage]

Reference 1 - 2.58% Coverage

They do understand because we tell them to go and see how the sample house was done. They are allowed to get inside the sample house and compare with theirs. They are able to go and do the same with their houses

**P8:** I think houses are well improved but due to heavy winds, it destroys the window where gauze wire was nailed, this becomes a draw back. We tell the house owners to keep on improving their houses if the houses were destroyed. We inspect the houses to see if all houses are improved and once we get a house that is not well managed, we tell them to re do the work. To add on that, if we tell the owner to redo the house and he/she has done the same, we just do the work ourselves and when we do it, we punish them because when others are building, they leave the gaps for the cats to use the gaps when getting inside the house, we do not leave any gap. We then warn them that if they continue, the issue should be reported to the village headman and tell the village headman that you are destroying our gauze wire

<Files\\FA-A Community participants FGD 2> - § 3 references coded [7.34% Coverage]

Reference 1 - 4.72% Coverage

The houses in our village are properly improved.

**Facilitator** Alright. P3, perhaps you can explain to me what makes us realise that this house is properly improved?

**P3** What makes us realise that the house has been properly improved is that when we get inside the house, we do not see light getting through spaces. It is only when you get inside that you realise that the house has been properly improved. It does not show any spaces.

**Facilitator** Alright. That is P3’s idea. I would like to hear opinions from other villages. P7, are all the houses in your village properly improved?

**P7** The houses in our village are properly improved because the committee member come to check. If they see that there are still spaces, they tell us to close with mud. Currently all the houses are good condition.

**Facilitator** Alright. P4, you have additions?

**P4** It I true. In our case, the committee members come to check how we have tacked the gauze wire. If there are remaining spaces, they tell us get mud and close them.

**P2** The houses are really being properly improved. But we should not hide the fact that heavy rains we had in 2019, many houses fell. So you cannot say a house is properly improved when, for example, a brick house has a fallen wall replaced with grass wall which has even been finished closing the spaces with mud. So we cannot say all the houses are in good condition. Thank you.

**Facilitator** Alright. P1, yes?

**P1** In Kamoga village, three houses fell. I have given out of my houses. So we cannot say they are being bitten by the mosquitoes because some of them are sleeping in houses with a fallen wall. Many houses have fallen down because of the rains. They now have to make bricks and build the house.

Reference 2 - 1.19% Coverage

Yes, they follow. They have seen the advantage of improving houses and sleeping in proper houses.

**P7** They have seen the advantage of closing eaves because they are not frequently getting malaria.

**Facilitator P3?**

**P3** They are seeing the advantage because before they did the House Improvement work they were frequently suffering from malaria while now they are being protected. So they think it is important to improve their houses.

Reference 3 - 1.43% Coverage

So I would also like to know from you. Do you think it is possible for everyone in the village to follow these methods? Let us perhaps start with P7.

**P7** Yes, it is possible to follow these methods because they are protecting them from malaria.

**P6** It is possible for every one of the people to improve their houses because they are seeing its advantage. They are being protected from malaria.

**P5** Yes, it possible because they have differentiated between the previous and current situation after improving the houses.

<Files\\FA-A HI Committee FGD> - § 2 references coded [5.27% Coverage]

Reference 1 - 3.13% Coverage

How do you know that the house is been well managed when closing the open eaves?

**P2:** For us to know, we first inspect the outside and if it’s okay, we say ‘’chikome kome cha mkuyu’’ (*appearance deceives*) then we get inside and spend a little time so that our eyes should be seeing properly. Then if the house has holes, we are able to see them but if we don’t see them, we then conclude that the house is properly managed.

**P3:** Every house in Mtemera is properly closed and has been inspected as P2 has said that for us to confirm that the house is properly managed, we the committee members do get in and close the door then. When there is darkness, we look where there is light, that’s how we did it

**P7:** The houses are properly managed because even the owner was taking part, if there is somewhere that it’s not managed, we were discussing with the owner to do it

Reference 2 - 2.14% Coverage

Okay let’s proceed. I would like to know, do community members follow the right house improving procedure?

**P2:** people follow because we teach them because when they have done it today, the mud starts cracking, and then we tell them to repeat the same. After they repeat, we tell them to get inside the house and see if there is any hole, so they follow it and they accept it. When we go for inspection, we find exactly what’s supposed to be done

**P4:** people follow it because we taught them at the beginning

**P1:** just too add, people follow it because we taught them, it’s no longer a new thing

<Files\\FA-B community participants FGD> - § 1 reference coded [1.91% Coverage]

Reference 1 - 1.91% Coverage

Thank you. Are there any additions on this issue? Alright. I would also like to know that; do the people in your villages follow these standard methods of House Improvement? ***(Question repeated)***.

**C2** People follow the standard methods for improving houses because they see they how the leaders have done their houses and tack the gauze wire.

**Facilitator** Alright. Are there any additions, K2?

**K2** I do not have much additions. It requires the people who were first taught how to do the work to do the best. This is because this person will be an example to the people. They can discourage people if they tell the people to improve their houses properly when theirs is not properly done. So we want to encourage the people who were chosen to be inspecting the houses that they should have properly improved houses so that when they tell people, they should be an example. For example, you cannot not be an agricultural advisor when you cannot have food yourself. It means you are failing your work.

<Files\\FA-B HI Commitee members FGD> - § 2 references coded [1.79% Coverage]

Reference 1 - 1.25% Coverage

what shows that the house has been properly closed in the open eaves?

**P2:** what shows is that the gauze wire is properly inserted as well as open eaves are properly closed, thank you

**P8:** such house shows when you get in the house, there is no hole where mosquito can get in the house through

**P5:** if the house is closed in the open eaves and when get in, it’s not only open eaves that we look for, also in the gaps between the roof and the poles. We close such open eaves so that there should be only darkness in the house

Reference 2 - 0.54% Coverage

they follow because we were closing 3 or 4 houses then we were calling people to come and see how the houses have been improved

**P6:** That’s true because we were calling people to coma and see how the houses have been improved.

<Files\\FA-C Animators FGD> - § 4 references coded [5.93% Coverage]

Reference 1 - 0.51% Coverage

okay. In your opinion, do you think there is proper closure of open eaves in all the houses in your village?

P6: yes in our village open eaves are properly closed. Out of 100 houses just one or two may not have open eaves properly closed maybe because the owner declined to improve their house due to misunderstandings between them and the chief.

Reference 2 - 2.01% Coverage

if you could expand on that, how do you tell if a house has been improved properly?

P6: to tell whether a house has been improved properly, we look at how the gauze wire has been fixed, checking for spaces through which mosquitoes can enter the house. Inside the house we check for small openings and that if there’s any light from outside then it should be through the windows only. The other thing are stories that people tell that there are fewer mosquitoes entering the houses. Quarterly reports [about malaria cases] from the clinics also match with our records.

P3: open eaves are properly closed in all the houses. How do we know that open eaves are closed properly? First, we check if the gauze wire has been fixed as required, there are no spaces through which mosquitoes can enter the house. Then we go inside and close the doors to see if there’re any spaces letting light in, and we advise them to close the spaces if we identify any. So we tell that by doing external and internal house inspection.

P2: first, we inspect all the houses, checking how open eaves have been closed. If they are not properly closed, we advise them to close and leave no spaces between the wall and the roof. After the inspection, we call for a meeting where we tell them “If you need gauze wire we have it, we’ll start distributing it on such and such a date.”

Reference 3 - 2.38% Coverage

you have talked about how you tell whether open eaves have been properly closed. I want to hear from you that do people in your villages follow house improvement methods after seeing the demonstration house?

P1: people in the village, after seeing the demonstration house, use the demonstration house as an example of how their houses should be improved. We made sure that houses for demonstration be those of committee members because we wanted the houses to be accessible and belong to someone with knowledge of the project and who can improve their houses accordingly.

P6: when people see the house for demonstration, there are some who think will receive gauze wire quickly if they act quickly on improving their house. Of course, the idea of house improvement is not to make the house beautiful, but rather to limit entry of mosquitoes into the house. Now, when we are doing internal house inspection, if there are spaces identified, there are some who want to seal the spaces in no time and anyhow just so they can access the gauze wire quickly. Because they think delay will cause them to miss out on receiving the gauze wire. So, what’s required is that committee members regularly perform the scheduled house inspection visits.

P3: when people see the house for demonstration, they are able to improve their houses based on how the demonstration house is done. After working on their houses, they think they are done. But as committee members and animators, we conduct household visits. During the visits we identify spots that have not been fully sealed and advise that they complete them.

Reference 4 - 1.03% Coverage

do you think it’s possible for people to follow these methods?

P6: yes it’s possible for people to follow these methods of house improvement because it’s one way of limiting entry of mosquitoes into the house, because mosquitoes are responsible for malaria spread. But they not only need to improve the house they also need to sleep under a mosquito net.

P2: um, when they have improved the house, with the windows properly sealed, they should not ignore the swamps around them. We provide them with guidance on how to empty the swamps. Because they might be spending time outside the house with no idea that mosquitoes are breeding from the swamp, will bite them and return to the swamp.

<Files\\FA-C community participants FGD 2> - § 4 references coded [6.86% Coverage]

Reference 1 - 0.45% Coverage

Some houses are not properly improved. This is because there are some people who do not understand it and do not improve their houses properly. However, those who understand it improve their houses properly.

Reference 2 - 0.48% Coverage

Some people use grass to hold the gauze wire. When the grass is not holding it properly, the gauze wire will open on one side and close on the other side. So, not all the houses are properly improved due to lack of nails.

Reference 3 - 2.70% Coverage

Alright. That is P6’s opinion. Let us hear opinions from other villages. So, I would like you to tell me if people follow the standard ways of House Improvement when you are explaining. Do you think it is possible to follow these methods? Let us hear other opinions.

**P7** The people in the village follow these methods depending on how you explain to them on the procedure of the House Improvement. It becomes possible for them to use the methods.

**Facilitator** Alright. Okay. Let us hear pinions from other villages. P2?

**P2** My opinion is what I have explained already at first that when you are closing the door, it looks dark inside the house and no spaces through which mosquitoes can enter are seen. You have to make sure it is dark inside the house when you close the door. That way we say the house has been properly improved.

**Facilitator** Alright. P2, what I wanted to know is that do the people in your village follow these methods of House Improvement as you have explained?

**P2** Yes, they are following.

**Facilitator** Alright. Any other opinions? Let us hear from other village.

**P4** These methods are being followed. This is because mostly when the houses are improved, they are done properly as instructed by the committee members.

Reference 4 - 3.23% Coverage

Alright. Let us finalise this question by asking this question; what can encourage people in the village to follow these standard methods of House Improvement? ***(Question repeated for clarity)***. Let us start with P5 then P1.

**P5** Many people have seen the reduction in malaria prevalence because mosquitoes do not enter the house due to the improvement. If the mosquitoes do enter the house, they are protected from the mosquito nets. Their friends can see the change till now.

**Facilitator** Alright. Let us hear from P1.

**P1** When you improve the house properly, the passers-by admire it and the people sleeping in that house do not frequently fall sick. So, people admire such a family.

**Facilitator** Alright. P6?

**P6** There is need for encouragement from the committee and amongst ourselves instructing each other how to best improve the house. We just need to encourage each other.

**P7** The committee should be having some of the materials which are scarce in this work we are doing. These materials should be available with them so that when we say we need certain materials, we should be able to find them with the committee members. This can encourage other people.

**Facilitator** Alright. P4?

**P4** I think what can encourage them is that they can differentiate previous times and current time. They can see that they were having a lot of mosquito bites previously compared to the times after improving the house. This can encourage them to maintain the house when it has been damaged.

<Files\\FA-C HI Committee members FGD> - § 3 references coded [3.95% Coverage]

Reference 1 - 1.24% Coverage

Do you think every house in your community properly closed?

**P7:** all houses are properly closed because when someone has closed the house, we tell him/her to come and report for us. When we are told, we the committee members go to inspect the house. We close the door to verify if what he/she is telling us is true or not. If we see that there is a gap, we tell that person that there is this gap and that gap then the person maintains the gaps

Reference 2 - 1.85% Coverage

okay, can you clarify on what tells you that this house has properly closed in the open eaves?

**P7:** we know by getting inside the house and then we close the door. We then look onto the walls and where there is light, we know that there is a whole that needs to be closed. We also look up to the roof to see is there is some light, we know that there is a gap. So where there is darkness in the house, we know that the house is properly managed

**P1:** All houses in our community are properly closed because we the committee members take part to inspect the houses

**P5:** All houses are properly managed because we do plastering the wall so that there should no gaps

Reference 3 - 0.86% Coverage

procedures of house improving?

**P6:** when we tell people what to do, they follow and we go to inspect. We close the door to see if they have done it properly. We do this with every house

**P7:** To add on what P6 has said, people follow it because they see the advantage of it and they started following it easily

<Files\\GVH KAMOGA> - § 1 reference coded [3.04% Coverage]

Reference 1 - 3.04% Coverage

do people in your village, chief, follow proper standards for improving houses?

Respondent: they follow, fine

Interviewer: what is it that can encourage people to follow these ways?

Respondent: what we see is that when building the houses, someone sees that my friend is building this house and you also wish to build a similar house, and it spreads that

<Files\\GVH MAKANDE(1)> - § 2 references coded [5.49% Coverage]

Reference 1 - 1.78% Coverage

Respondent: aaa it is not that hard work. If it is a newly built house we just encourage the owner to improve the house to the standards of this intervention, aaa it is the good job

Reference 2 - 3.71% Coverage

Alright. Do you think House Improvement is done to the good standards in all houses?

Respondent: Yes they are done to the best

Interviewer: Alright. What are the indicators that the house has been done to the good standards?

Respondent: For the committee they move around checking the houses, if they are satisfied we the chiefs we know that our people are been protected

<Files\\HSA Bwalo Transcript KII> - § 2 references coded [5.01% Coverage]

Reference 1 - 3.86% Coverage

These houses have house improvement done to standard.

Interviewer: Can you please describe what you know when you say this house has a good standard for HI in the villages?

**Respondent**: What happens is that when people have done house improvement, we make follow-up visits to see the quality of work on the houses so when we arrive at any house, we check at all the openings that are remaining, even if they have closed the eaves and placed wire gauze on windows, we just need to find the openings. If we find any opening we tell them right away. So that they should close the openings. They have to know that the openings left can cause the mosquitoes to enter and this may result in them suffering from malaria. The key is providing these supportive supervisions.

Reference 2 - 1.15% Coverage

They HI is done to standard and again we provide some follow up visits for us to see if everyone has done HI to standard. Of course there would be some that will have houses with house improvement not done to standard. It happens.

<Files\\HSA Liwonde- Kapichira Health Center KII> - § 1 reference coded [6.27% Coverage]

Reference 1 - 6.27% Coverage

Alright. Let us go to the last section. I would like to get your opinion. If you look at all the houses in your village, are they all properly improved?

**HSA** There are some houses which are not properly improved perhaps because of the designs of the windows. There are windows which open to inside while others open to outside. So, it is difficult to tack a gauze wire. However, they still close it with gauze wire.

**Facilitator** Alright. I would like to know from you about what indicates that a house has been properly improved?

**HSA** When you get close to the house you check if there are no spaces around the margins of the gauze wire through which mosquitoes can enter the house. If there is a space, the house has not been properly improved. A well improved house requires that the gauze wire touches the window frame without leaving any spaces.

<Files\\KII Chief Chambo FA-C> - § 2 references coded [7.48% Coverage]

Reference 1 - 4.41% Coverage

Thank you very much; do you think all the houses in your area are been improved according to standards?

Respondent : Yes, they are been improved according to standards

Interviewer : what are the indications that the house has improved according to standards?

Respondent : We make sure that the eaves have been closed, when the house is grass thatched, we make sure that the eaves and the grass are in contact and closed and that the wire gauze has been properly placed. Some leaders may also enter the house to see the parts that are remaining

Reference 2 - 3.07% Coverage

All right, are people in your area follow the standards for improving the houses

Respondent : Yes, they do follow

Interviewer : Do you think it is possible to follow the standards?

Respondent : it is possible

Interviewer : what can encourage people in the community to follow these standards?

Respondent : regular meetings and people can follow for what malaria project is doing

<Files\\KII Chief Goliati> - § 4 references coded [6.81% Coverage]

Reference 1 - 1.88% Coverage

Are they improved according to the standards?

Respondent : ohoo, some of them fail to do it, because when they take it on their own hoping that they will manage after they have seen from other houses, so when they take and do what they know, I then send the committee to supervise people who have been given the wires to do the job on their own. So they become prepared with the humour which they received from the Organization

Reference 2 - 0.54% Coverage

the committee start organising and helping him up to the end. They find that others who understood have done if correctly

Reference 3 - 2.40% Coverage

house has been improved according to the standard, what do you notice if the house has been improved according to the standards?

Respondent : Eyeahh, we notice when we have entered the house because it is different from looking at it from the outside, when we look at it from the outside we may mistakenly assume that it is fine, but when we have entered

Interviewer : mhmm

Respondent : when we have entered we then look what is outside, so we spot the places which were left if there is light and that mosquito can enter

Interviewer : mmm

Reference 4 - 1.99% Coverage

Are people from your community follow these standards for house improvement?

Respondent : They do follow

Interviewer : mhmm

Respondent : Absolutely

Interviewer : Alright, I want to know further, do you think it is possible for everyone to follow these guidelines?

Respondent : It is very possible

Interviewer : mhmm May you explain further

Respondent : It is possible for everyone to follow this strategy, for his or house to be protected

<Files\\KII Kalinjala> - § 1 reference coded [6.53% Coverage]

Reference 1 - 6.53% Coverage

alright, moving to the last section. Do you think House improvement is been done in better standard in all houses of your community?

Respondent: yes

Interviewer: what are the indicators of improved house?

Respondent: we check for eaves and windows, if they are well closed we recommend

Interviewer: alright does the community stick to these standards?

Respondent: yes

Interviewer: what can influence the community to stick to these standards?

Respondent: they can do this to prevent malaria.

<Files\\KII MDZACHI(1)> - § 4 references coded [4.61% Coverage]

Reference 1 - 0.66% Coverage

in your opinion, is house improvement done to standard in all the houses in your village?

Respondent: they are done to standard

Reference 2 - 0.68% Coverage

Respondent: not all are to standards

Interviewer: mmm

Respondent: there are some houses that are covered to according to standards

Reference 3 - 1.63% Coverage

alright. So I still want to know on the same chief, how do you know that this house has been well covered or well improved?

Respondent: you see it. If you see you are able to know that this house has been covered well. And you ask the owner, who covered the house for you? Aaaa they bare the same boys from this village

Reference 4 - 1.64% Coverage

alright. I still want to know, you have said that you enter inside the house and see if there are holes or what, I want to know there chief, do people in your village follow these standards we have discussed here?

Respondent: others follow since people are difficult

Interviewer: mmm

Respondent: others do not follow

**Description:** Explains the participants' perspectives on the changes observed in their communities as a result of the HI and their recommendations for how to better involve their communities in HI activities.

<Files\\Community participant chambo IDI> - § 4 references coded [12.26% Coverage]

Reference 1 - 1.77% Coverage

**INTERVIEWER**: how satisfied with the house improvement of your village? Like your village Chambo

**PARTICIPANT:** we very much satisfied because we work accordingly

Reference 2 - 2.59% Coverage

**INTERVIEWER:** based on malaria, what changes have you since this project start?

**PARTICIPANT:** we don’t see people frequently suffering from malaria

**INTERVIEWER**: what is the biggest change?

**PARTICIPANT:** we are not suffering malaria

Reference 3 - 3.32% Coverage

**PARTICIPANT:** just encouraging those that they are lazy in house improvement activities that they should be sleeping in the mosquito nets and do proper house improvement standards. We are working on our activities very hard because of not going to the hospital because of malaria, we are staying healthy.

Reference 4 - 4.58% Coverage

**INTERVIEWER**: thank you so much. How can you explain house improvement intervention to the village which is starting this intervention?

**PARTICIPANT:** house improvement is very good because it is simple. Once the committee say want to improve your house it takes small time because committee contains a lot of people. Just encouraging the people that they should accept so that they should not frequently suffering malaria

<Files\\Community participant Chaziya IDI> - § 2 references coded [2.67% Coverage]

Reference 1 - 1.61% Coverage

**INTERVIEWER:** so how satisfied are you with house improvement of your village?

**PARTICIPANT:** am satisfied because people who come to improve our house are very unique and trained people

Reference 2 - 1.07% Coverage

**PARTICIPANT:** in my area before we had high prevalence rate of malaria, now with this experience malaria has been reduced

<Files\\Community participant chaziya-chapananga IDI> - § 1 reference coded [0.90% Coverage]

Reference 1 - 0.90% Coverage

**PARTICIPANT:** because of wire and mosquito nets, there is the change of the diseases burden

<Files\\Community participant Garonga IDI> - § 3 references coded [4.81% Coverage]

Reference 1 - 0.80% Coverage

**PARTICIPANT:** am satisfied, because before we were frequently suffering from malaria but now malaria has been reduced

Reference 2 - 2.13% Coverage

still I want to know, what changes have you observed/seen in terms of malaria since house improvement started

**PARTICIPANT:** now we have seen the change (laughing)

**INTERVIEWER:** tell me

**PARTICIPANT:** explain again

**INTERVIEWER:** on malaria what changes have you seen in your area since house improvement started?

Reference 3 - 1.88% Coverage

**PARTICIPANT:** we have seen the benefits because since we started house improvement malaria has been reduced people are not frequently suffering from malaria. Before house improvement, different insects were entering the house but after house improvement we have experienced benefits

<Files\\Community participant Goliati IDI> - § 2 references coded [3.60% Coverage]

Reference 1 - 0.99% Coverage

encouraging them to close the eaves because malaria is very dangerous you can die because of it.

Reference 2 - 2.61% Coverage

I can tell them that if they want to reduce prevalence of malaria in your community, you are supposed to put gauze wire at the window, close the eaves and that there should be no light upside of the house and close the eaves properly, I can give such message

<Files\\Community participant Kabwatika IDI> - § 5 references coded [14.49% Coverage]

Reference 1 - 0.65% Coverage

am satisfied based on the advises which they give to us, and am very much satisfied

Reference 2 - 1.85% Coverage

**PARTICIPANT**: I have seen changes because malaria cases have been reduced. The house should be built properly put gauze wire all the eaves and small holes should be closed. There is change than before and we have used the advices and malaria has reduced.

Reference 3 - 5.66% Coverage

**PARTICIPANT**: personally I have seen the change, at my house my children were frequently suffering from malaria because the father was not willing to improve the house so every time when my children go to the hospital were found malaria positive. And the doctor would keep on asking ’you came last week found malaria positive what is happening? Is the house improved? ’ I was not hiding anything by telling him that the house is not built properly. So what you have to know there will be malaria all the times in your house and malaria will be only the disease in your house. So the father understood the advices from the committee. Later on the child was not found with malaria meaning to say malaria has at least reduced and we have used what the committee has advised us to do.

Reference 4 - 1.69% Coverage

**PARTICIPANT**: I can tell them that malaria to be reduced we should improve the house and build it properly and put gauze wire and close all the eaves and sleep in the mosquito net; problems in terms of malaria are likely to reduce..

Reference 5 - 4.64% Coverage

I can tell them to build the house smartly, close all the eaves and there shouldn’t be seen holes and put gauze wire and sleep in the mosquito net, hence the person can understand and improve the house but if you undermine disease can be in the house always, but if we understand this malaria can be reduced because we have some people in the villages who do not abide the law and the committee keeps on advising them hence their houses are still struggling with malaria. If the person understand the message can do better because there are some people even if they hear the message they don’t follow hence you experience a lot of problems

<Files\\Community participant Machokero IDI> - § 2 references coded [1.42% Coverage]

Reference 1 - 0.93% Coverage

**PARTICIPANT:** I said there is change because now malaria cases it seems have decreased.

Reference 2 - 0.49% Coverage

**PARTICIPANT:**  decrease in malaria prevalence

<Files\\Community participant Mtemera IDI> - § 1 reference coded [0.96% Coverage]

Reference 1 - 0.96% Coverage

**INTERVIEWER:**  how satisfied are you on quality of house improvement of your village

**PARTICIPANT:** quality of house improvement in my village

<Files\\Community participant Weremu IDI> - § 1 reference coded [1.49% Coverage]

Reference 1 - 1.49% Coverage

**INTERVIEWER:** are you satisfied with house improvement?

**PARTICIPANT:** yes

**INTERVIEWER:** how satisfied are you?

**PARTICIPANT:** we understand each other on HI and every house seem very good

<Files\\FA-A Animators FGD> - § 8 references coded [12.47% Coverage]

Reference 1 - 0.87% Coverage

We are going into another section. As animators, is every house in your community improved under HI project?

**P3:** The houses are well improved because we were inspecting together. We could tell each other how to work until every house has improved

**P1:** Every house is improved because as a leader, you make sure that everything is done accordingly

Reference 2 - 1.60% Coverage

you need to get inside and look up, look on the walls to see if there are gaps. There should be total darkness inside and no light should be getting inside. You should look if the gauze wire was well nailed. If everything is okay, the house was well managed

**P4:** In our community, every house was well managed because we have a sample house where people go to see how they should do their houses as well. We do inspect as well if people have done like a sample house

**P2:** As my friend has said, for use to see if the house is well managed, we get inside the house and close the door and if there is somewhere light, we are able to tell.

Reference 3 - 0.61% Coverage

We should be having community meetings so that people should get involved

**P5:** we should keep on conducting the community meetings because some people are not willing to do the right things, as leaders, we should keep on doing our job on wards

Reference 4 - 1.65% Coverage

There is a change because when we are conducting the community meetings, we ask people, ‘’how many here are diagnosed with malaria?’’ Only one or 2 people could raise hand and I believe some of the evidence is here at the hospital. We come to ask how many people from Machokero village have diagnosed with malaria this month, there can be 2 or 3 people only unlike in the past, and more people were diagnosed with malaria

**P4:** there is a change because previously, we the number of children who die of malaria was high. When the malaria project started training us, there is change because when the child has fever, they are taking that child to the hospital

Reference 5 - 1.59% Coverage

People now know the signs of malaria, when the child faints, they know that it is severe malaria. If the child has fever, they should rush to the hospital for the child to be tested to see whether it is malaria. In the past, when the child has fever, people were only thinking of witchcraft, but now, they know that it is malaria

**P2:** The issue is to encourage people to keep on sleeping under the net because you can happen to have no malaria [plasmodium](https://www.google.com/search?client=firefox-b-d&sxsrf=ACYBGNSxywH_q79YQ60gkVtMCsAejwATiw:1568349960577&q=malaria+plasmodium&spell=1&sa=X&ved=0ahUKEwiPlaLy_szkAhWyQEEAHZYXDuIQBQgtKAA) but you have received a visitor who has malaria plasmodium and if you do not sleep under the net, mosquito can bite the one with the plasmodium and bite you, you can catch malaria

Reference 6 - 1.73% Coverage

The projet will continue because it is not benefiting Majete staff who came here to train us, they came only to train us and we should continue with the project

**P5:** I think this project will continue because we have skills and community members now know the danger of malaria, so it cannot stop because it is protecting our lives and we are not wasting money for medication on the malaria

**P2:** Majete was a light for us because they are wishing us the best on our lives and we cannot stop because in the end, we shall die and others will have to carry on with the skills. We thank you for this program and we should not stop doing it because our children should also be taught about this

Reference 7 - 2.43% Coverage

If Majete leaves us alone, as volunteers we shall continue doing the job because we have skills and we have learnt a lot and we cannot stop training people because Majete has left us

**P7:** The work will continue because the Majete and the Hunger project are not doing these for themselves but for us. When we are doing this work, we should know that we are doing for the sake of our lives to have enough protection, so we will continue

**P3:** we will continue doing this because you have given us enough skills, we shall continue teaching our children for them to continue also doing this job and see the benefits of this than hearing the history about this work, they should be doing this to them and for others as well

**P5:** We shall continue because this is voluntary based work. If it was a paid job, we would have said that there will be no one to pay us, but this is voluntary work just to improve our livelihood, so together with the chiefs, we will continue this work

Reference 8 - 1.98% Coverage

I think these people will continue working because they have been trained and they knew the truth that the work is voluntary based, so they will have to continue with this up until malaria will no longer be the issue here

**P5:** These committee will continue because as animators, we are working together with the committees, so this will continue together with the chiefs as well as the community members

**P1:** If you stop even today, the committee members will continue and this will continue as well

**P2:** Our work will go on even if we have one committee member who is not willing, we can sit down with the chief to choose another person to replace that one, even if two people are not willing, we shall replace them with others who are willing to do the work. I hope the work will continue.

<Files\\FA-A Community participants FGD 2> - § 11 references coded [10.33% Coverage]

Reference 1 - 1.17% Coverage

What is needed is you to be vigilant encouraging the people. When they see your hardworking, they will be encouraged.

**Facilitator** What do you mean when you say “you”.

**P4** I mean us as committee members we should be vigilant encouraging the people.

**Facilitator** Alright. Any other opinions?

**P6** I think it is possible if we can be helped with the gauze wire and other materials needed for closing eaves. People can work hard.

Reference 2 - 1.43% Coverage

We are satisfied with the House Improvement method because we see that the house looks better than one with spaces through which mosquitoes can enter. The improved house looks good.

**P3** We are satisfied. The committee members do come after the work to check inside the house if there are any spaces. When there are no spaces, they accept that it has been properly done. So all the houses are checked and approved by the committee. If a house still has spaces, the committee tell the owners to properly close the spaces.

Reference 3 - 1.21% Coverage

We are satisfied because houses are looking good. There no any other problem. People are not falling sick frequently. Thank you.

**Facilitator** Alright.

**P1** We closed the eaves of our house in the village. It is an exemplary house in the village. We told the committee to be showing the people how the house has been improved. When every person comes, even when the gauze wire is there, we tell them to look at the house. There are no spaces.

Reference 4 - 0.32% Coverage

We have seen the change. There is been a reduction in malaria prevalence from the time this work started. Thank you.

Reference 5 - 0.90% Coverage

The main thing which has changed is that when we sleep in improved houses as well as under mosquito nets, we are not getting many mosquito bites. So there is a reduction in malaria prevalence. About ten people could get sick previously while now about two people are being found with malaria. That’s the change we are saying.

Reference 6 - 0.64% Coverage

The time that House Improvement program started, from 2015 till now, there has been a change. People are not frequently getting malaria. When they get to the hospital and get tested, they are not being found to have malaria. Thank you.

Reference 7 - 0.96% Coverage

Besides that, there is a big change. People are no longer dying due to malaria since that time. Things have changed now. Thank you.

**P1** We see that there is a difference between nowadays and previous days. We are now protected from malaria. When we get a child with body aches to the hospital, they are telling us the child does not have malaria.

Reference 8 - 0.28% Coverage

I can tell them to be responsible for improving their houses for them to prevent malaria. Thank you.

Reference 9 - 0.78% Coverage

I can explain to them the importance House Improvement to prevent mosquitoes from entering the house.

**Facilitator** Alright.

**P1** I can tell that once they are done building their house, they should close the eaves and tack the gauze wire to prevent mosquitoes from entering the house.

Reference 10 - 1.13% Coverage

I can explain to them that this work is very important and that they should not take it lightly. It helps in terms of malaria. I can also explain to them the advantage we have seen since we started closing the eaves and spaces as well as tacking gauze wire. The advantage that there is reduction in cases of malaria and that there are no more deaths of children and pregnant women due to malaria in our area.

Reference 11 - 1.51% Coverage

I can explain to them the importance of closing eaves and tacking gauze wire as the mosquitoes enter the house through open door only. If you sleep under the mosquito nets, you will be bitten. You prevent malaria that way. Thank you,

**Facilitator** Alright. Any other opinions? How can you explain to other villages which are just starting the House Improvement work, P7?

**P7** We can explain to them the good things we have seen in our village. Then we can teach them how we were doing the work of closing eaves as well tacking gauze wire in windows.

<Files\\FA-A HI Committee FGD> - § 6 references coded [12.61% Coverage]

Reference 1 - 1.95% Coverage

How satisfied are you with the closing of open eaves in your communities, I should start with P6:

**P6:** I believe because I do research. I inspect the house, then I ask the owner’s permission to get in the house for the inspection then I get satisfied that the house is properly closed

**P3:** I am satisfied because in Mtemera village, malaria is been reduced than before project came, thank you

**P2:** I would like to add, I can differentiate now, in terms of malaria. People are not getting sick now comparing with the past, so I am satisfied

Reference 2 - 1.76% Coverage

Lastly, II would like to know, what change have you noticed from the time when the house improving started?

**P2:** I have noticed the change as I said that malaria is reduced and the money that we spend at the hospital when we had malaria, is now being used on other thing

**P4:** In our community, I don’t hear people complaining on the issue of malaria these days.

**P5:** There is a change because malaria is been reduced and people are able to use money that could have been spent on malaria.

Reference 3 - 1.92% Coverage

What comments can you tell people in your community so that they can take part in the house improving work?

**P8:** In community, we think we have to encourage them to keep on closing their open eaves so that if their houses are properly closed and they sleep under the nets, malaria can be reduced

**P3:** On this issue, it’s so beneficial to them because if they close the houses on their own, we feel good as committee member and now, we conduct the meeting more often so that we can have the chance to remind them, thank you very much

Reference 4 - 2.33% Coverage

First of all, I will explain the challenges that we had in Mtemera in terms of malaria and how it was reduced. Then I can start train them how to close the open eaves as well as how to insert the gauze wires. Then Ii can tell them the danger of malaria.

**P6:** I can start with the meetings with people in such communities. I will then tell them the advantages of malaria reduction then I will train them how to close the house and inserting the gauze wires, thank you

**P8:** First of all, I can explain to them how we started the work in our community, then I would tell them the advantage of this work, then I can train them how to do it, thank you.

Reference 5 - 2.51% Coverage

okay, let’s proceed. Do you think the work will proceed when the project shall phase out?

**P5:** In our community, this will continue because it’s helping us. We shall continue with the meetings

**P3:** In Mtemera, this will proceed because the danger that was there before, could come back if we stop this

**P7:** This will continue based on the 5 points that hunger project tell, thus mind change, commitment, good leadership, so following that, we the owner should know that it’s benefiting us, thank you

**P8:** This will continue although it will need us to buy gauze wires, it will still proceed. For those that cannot afford, there is another way, they can cut the net to use instead of gauze wires

Reference 6 - 2.14% Coverage

Finally, you as committee members, will you proceed to work after the project phase out?

**P1:** we will proceed because we have been trained and we have noticed the advantage of preventing malaria in our community.

**P4:** The project will continue because people have seen the advantage of malaria prevention

**P3:** This will continue because if we don’t continue, we will go back to the same problem of malaria and we will wrong people who are just come in the community and are not trained

**P5:** we will continue because we have seen the benefits, so we will continue with the meetings, thank you.

<Files\\FA-B community participants FGD> - § 3 references coded [7.90% Coverage]

Reference 1 - 2.12% Coverage

Thank you. Alright, let us continue. On the other part, I would like to know is; what changes have you seen in your village since House Improvement work started in as far as malaria is concerned? ***(Question repeated)***. Perhaps let us start with K3. We want to know what mainly has changed on malaria.

**K3** The main change is that many people were frequently suffering from malaria previously. Additionally, most of the people were suffering from severe malaria. When some starts feeling unwell in the morning, for example, they could be losing consciousness by afternoon. However, nowadays with the coming of House Improvement project, most of the malaria cases are mild.

**Facilitator** Thank you. Any other additions?

**K4** There is been a great reduction of malaria prevalence in our area. For example, in every ten individuals, perhaps only three or two are found to have malaria in a month. This is because people are properly following the methods being taught by the Majete Malaria Project team. Thank you very much.

**Facilitator** Are there any other additions? C2, do you have any additions on this issue?

Reference 2 - 1.93% Coverage

B1? Alright, let us continue. How can you explain to the people in your village for them to take part in this House Improvement work?

**K2** We have to encourage people through animators. As we have explained earlier that frequently calling for gatherings, is one way through which people keep learning. In this way, you keep reminding the people now and again. Or else, animator should know how many houses are in his or her village and how many of the houses have not been improved. They should not get tired of the work. That will be one way to frequently remind people of this work.

**Facilitator** Thank you.

**K4** We can also encourage people to be attending the gatherings that the Majete Malaria Project team and animator call for so that everyone should learning or hearing on their own whatever is being said or taught in these meetings rather than being told another person. This is because they get the first-hand information of everything that is said when they attend the meeting. Thank you very much.

Reference 3 - 3.85% Coverage

We can explain to other villages that tacking gauze wire is advantageous in that it reduces malaria prevalence, the house looks good inside and dangerous animals such as snakes do not get into the house.

**Facilitator** Thank you.

**B1** I only want to add. We can encourage people from other villages which are just starting the work that they have to know the importance of closing eaves as well as the importance of tacking gauze wire. They might not know because it has just started. The other thing is that the animators in such village should have some organisation. For example, they can meet me as B1 and I can talk to them if I know the things. When I teach them, they will teach the people in their village. They can teach the people by asking the chief to gather the people and then they can explain the importance of tacking gauze wire and closing eaves, what gauze wire protects from and what it closing of eaves entails. So we can encourage them in such a way when we meet them we should be able to explain to them the importance of tacking gauze wire and closing eaves so that we prevent mosquitoes from biting us in our houses. Thank you very much.

**Facilitator** Thank you. Are there any final opinions on this issue?

**K2** There is need for animators from villages which have been doing this work to be going out to these villages and hold meetings with them to encourage and help them because these are the people who have just started and they do not have much knowledge. So it will be like an example to teach them until they know how the work is done.

**B1** In addition to that, the Majete Malaria Project team should be organizing to meet animators in such villages, teaching them and guiding them on what they can tell the people in the village so that the people in the village should have the expertise. So I am asking that the project to reach to the people in villages which have newly been chosen or they have not been chosen so that they should also be protected from malaria. Thank you.

<Files\\FA-B HI Commitee members FGD> - § 6 references coded [13.11% Coverage]

Reference 1 - 0.87% Coverage

how satisfied were you with the closing of open eaves in your respective communities?

**P5:** we were satisfied because it’s one way of reducing malaria. We therefore ask for Majete malaria project to continue, thank you

**P9:** we were satisfied because when they did it, we went out and look from the outside and from inside, there was no light, so we were satisfied.

Reference 2 - 1.64% Coverage

what are the changes have you noticed since the house improving work started as far as malaria is concerned?

**P3:** there iis a change because in Kudzambwe, malaria is reduced because when the child has fever, he/she was diagnosed with malaria but now that does not happen. May be he can faint, thinking that it’s malaria, but the child is not diagnosed with malaria. If this will continue in future, we will not have malaria

**P6:** that’s true that in community, malaria is reduced because of these intervention of closing of open eaves and the distribution of mosquito nets

**P2:** adding on what number 6 has said. When this program was introduced, there was a change because malaria is reduced

Reference 3 - 1.34% Coverage

do you have any comments or idea that you can tell your fellow community members to take part in the house improving work?

**P1:** The advice is for those whose houses collapsed. They should be closing the open eaves when building the house and they should be sleeping under mosquito

**P7:** we should encourage them to sleep under mosquito nets as they wait for the rebuilt of the walls

**P4:** we should tell people that they should be sleeping under nets, closing of open eaves and using gauze wire because this has reduced the number of people suffering from malaria

Reference 4 - 5.42% Coverage

what can be followed for the community members to take part in the house improving work?

**P8:** we should encourage community members to close the open eaves and be sleep under the nets the use of gauze wire

**P4:** Another thing is that we had a showcase where we were explaining people on how this has benefited us. This should come in the communities because not everyone went there. There should be that everyone who answers the question should be given a t-shirt so that people will be encouraged

**Facilitator:** how can you explain to the communities that are starting the house improving work?

**P7:** we can tell them the benefits that we have seen following this programme. In the past, the money was spent at the hospital, but now it’s no longer the case

**P1:** we cann explain or we will explain the advantages that we have seen here comparing with the past. We can explain too to our friends

**Facilitator:** okay I will read again the question. how can you explain to the communities that are starting house improving work?

**P5:** we as people who once worked on this, firsly we can ask from their village chief to welcome us, and then we can sit down with them and tell them that first of all before someone put the gauze wire, we ask permission from the house owner. As a chief, he/she explain about the programe. It is the group that chose the committee of 10 people. We advice the house owner to get the bricks and close the open eaves and after that, one of the committee members ask permission from the house owner to get in the house to see if the house if properly closed in the open eaves. If the open eaves are been closed, the malaria project brings tape, sizzlers and gauze wire. Before cutting the gauze wire, we measure how many centimeters, we start with the length, then in the width then measure the gauze wire according to the window measurement then we cut the gauze wire. We get the nails of there are no nails, we get the bicycle spokes. We cut them into small pieces, then we get the small wood then we are ready to insert the gauze wire. We take the other 2 community members to be looking what is happening. holes, we tell them to close with the **saw dust.** We tell them that there will be mosquito distribution, so you should be sleeping under mosquito net

Reference 5 - 1.15% Coverage

okay, do you think the house improving will continue when Majete Malaria project will phase out?

**P4:** I think the programe will continue because we are trained and we are still working and as long as the gauze wire will be available, we will be working

**P6:** the programs like closing of open eaves will continue but with gauze wire, we will not afford because we cannot get the gauze wire.

**P2:** the work will continue apart from that of gauze wire because nobody can afford that

Reference 6 - 2.70% Coverage

As committee members, will you continue with the work when the project will phase out? If yes, you will tell us what procedure will you follow and if not you explain why not

**P1:** we will continue because we started this work and if leave it on the way before someone is trained, that person will not afford. We will continue this work for those who are just building the houses so that we can help them preventing malaria

**P9:** we will continue because it’s protecting our bodies. The concern is that where are we going to get the gauze wires, for brick and mad we can get them but the gauze wire. That’s where we need to think on how we can get the gauze wire and we don’t have money

**P8:** the work of will continue but with gauze wire, others will continue while others will not because the gauze wire is expensive. Malaria project is the one that buy this gauze wire for us

**P3:** As others have said, this will continue apart from that gauze wire but still on that one, everyone can make a decision based on the challenges that you are facing related to disease. You can make a budget to buy a gauze wire according to how you feel.

<Files\\FA-C Animators FGD> - § 4 references coded [16.73% Coverage]

Reference 1 - 2.74% Coverage

okay, winding up on this subject, I just want to hear your opinions, how satisfied are you as animators with how open eaves are closed in your villages?

P4: we are satisfied because the houses look good when we look at them.

F: what I want to hear is how satisfied you are with how people have closed open eaves when you look at the houses.

P2: One, I am satisfied with closure of open eaves if the roof and the walls are in contact. Two, if the house does not have holes - when I go inside I shouldn’t see holes on the roof, I shouldn’t see a torn plastic paper, and the grass on the roof should be tight together. Then I am satisfied with the house. If the house windows do not have gauze wire, I tell the owner that “Tomorrow I will start with you, handing out gauze wire.”

P6: in my village, I am satisfied with house improvement when malaria cases are minimized following the education that we gave to households. We pick maybe 10 households and conduct an assessment, asking the households questions about malaria, and they personally tell us, “There’re fewer mosquitoes entering the house, and we have malaria less frequently.” Based on that, we tell that malaria cases are reducing, and that they properly improved the house. You also ask them if you can check the house, and you inspect the entire house, seeing whether the windows are properly fixed, or open eaves are properly closed. So, in my village, as an animator, my satisfaction is when people tell me and I personally see it with own eyes.

P3: I am satisfied if people improve their houses according to the instructions that we gave, to prevent entry of mosquitoes. Also, when I check the house from inside and find that all the holes are properly closed, that’s when I am satisfied – the external appearance of the house and also the internal inspection of the house.

Reference 2 - 3.17% Coverage

thank you. Let’s now go to our last topic. What changes have you observed in your villages since commencement of the house improvement project?

P2: in my village, in terms of malaria cases, there has been change. One, infant mortality has reduced. Two, mortality of pregnant mothers has reduced. Three, deaths of elderly people have reduced. Why? Because in the recent years people have been sleeping in properly improved houses. Today, we are just expecting new gauze wire to replace the wire that has torn.

P1: in my village, there has been improvement because have changed their attitudes. In the past, people had negative attitudes towards use of mosquito nets. But today they understand the benefit of sleeping under a mosquito net, improving their houses, and they are doing things sensibly – they ask and respond to questions when they attend meetings. And they show so much interest in things that take place in the village.

P: in my village, I think there has been a lot of change. Concerning cases of severe malaria, before the project, and before we started teaching people, severe malaria used to be a very serious challenge. There used to be cases of people collapsing while out gardening, and people would attribute that to witchcraft or something else. But after we taught people about malaria, and when people had improved their houses, cases of severe malaria have reduced, or have completely stopped in this area. Um, the other thing is that we teach people to attend the hospital when they feel unwell. Now, when we are holding meeting and ask who has been to the hospital this month and who was diagnosed with malaria, most of them report of attending the hospital but only a few or two of them say were diagnosed with malaria. That’s what proves that malaria cases have reduced.

P3: changes contributed by the HI [house improvement] project can be seen in the numbers of people being infected with malaria, which have reduced.

P5: just adding on that, previously, if someone was sick, they would rush to a healer, saying, “I have been bewitched.” But today, when they feel unwell, they rush to the hospital.

Reference 3 - 1.62% Coverage

okay, so what recommendations would you give to people in your villages so that they participate in the house improvement project?

P6: what I could say to people in my village or in this focal area C is that house improvement is one approach that helps to limit entry of mosquitoes into the house. If you have fewer mosquitoes entering the house, then there will be a reduction in malaria, because mosquitoes are one insects responsible for malaria transmission. The other thing is that despite the malaria project coming to an end, or already ending, my request is that the house owner personally takes responsibility to improve the house. If they need support, they can contact the animator and committee members, both of whom are right there in the village. If the gauze wire is completely torn, the committee members or the animator might have some pieces of the wire and can help.

P3: I would just encourage people in my village to keep improving their houses, closing opening eaves and sealing small holes found on their houses. That’s what would help to completely reduce malaria.

Reference 4 - 9.20% Coverage

Now, suppose you were recruited again to work in the new villages, how would you teach the villages about the project?

P2: I was trained by the project and I have a certificate. I wouldn’t have a problem standing at a meeting, teaching about malaria. I will easily teach and people will clearly understand what the dangers of malaria are. I will teach people what leads to malaria and its end result, so that they understand the danger of malaria as opposed to AIDS. If I have AIDS, I will be okay as long as I am taking my drugs. But if I catch malaria today, I could die in no time. That’s what I would teach to the new villages.

P1: I would clearly teach them that the animator should take lead with support from committee members and the village chief. If there’s efficient communication between these groups of people, then the house improvement project will be successful. But if there’s no collaboration then the project will not succeed. When deciding on the demonstration house, it should be the house of the leader that received training in order to teach others. That’s how clearly I would teach to them.

P3: being recruited again to go and help in the new villages would be very exciting. Why? Because I already have experience gathered through working in my village, plus the training I received. So, it wouldn’t be a problem at all, because I have done the job for 3 years.

P6: just adding on what P1 and P6 just said, for the new villages around the Majete Game Reserve that had no implementation of the project, um, what I would teach to them is about challenges: “When you are doing house improvement, there are different challenges. Therefore, you shouldn’t stay idle, thinking the project will solve challenges. It’s better to sit down and discuss, so that the project runs effectively. The problem is particularly with committee members. Whenever someone joins a project or starts volunteering, they have expectations, and sometimes their expectations are not met. So they may stop prematurely, or say discouraging things to the villagers, “There’s no benefit in that.”” This is something that can be addressed through dialogue. There should be a working relationship between the animator, village chief and committee members, so that should there be any challenges, they can dialogue. The other thing I could mention is that house improvement is not for fun – we are not improving the house to make it look beautiful only, no. The idea is to limit entry of mosquitoes into the house. Mosquitoes are responsible for malaria transmission, and if their entry into the house is reduced, it means there’ll be a reduction in malaria.

I: okay. In your view, do you think house improvement will continue after the Majete project phases out?

P1: house improvement will continue. After the Majete Malaria Project phases out, we’ll continue to work in the villages, protecting our lives from malaria infection through sleeping under a mosquito net, closing open eaves and sealing holes on the house. We’ll do all this for the protection of our lives and of our children.

P2: I will continue doing this work. I won’t stop when the project stops. I will encourage people to improve their houses, sleep under a mosquito net, empty swamps, and break pots with unused water for safety from this dangerous female mosquito. I will continue doing this, because I am the one in the village with skills for this work.

P5: to continue doing this work won’t be strange because we are going to be the same people doing what was being done previously.

P3: the work will continue because its benefits are not seen by the project but by villagers themselves. So will carry on with this work to protect friends and families that are at risk of malaria.

I: okay, of course, P2 and P3 sort of responded to my next question, but I will ask it anyway just so I hear your thoughts. In your view, do you think you’ll continue your role as animators after the Majete project phases out?

P6: as P3 alluded to, our roles [as animators] will continue because the project mostly used us villagers to run the activities – they were not coming to teach people in the village – we were teaching people ourselves. So we have the skills. The other thing is that health workers were already teaching in the villages about malaria and other health issues before the project came, and through the project we have formed a collaboration with them. So, where the animator is weak, they will be there to teach about malaria.

P2: this work will continue. Why? Because I received training and have skills. So, even when those who trained me stay in Blantyre, I am confident to play the role of an animator because I received animator training. Even if they ask me to teach people how to take LA, I will be able to help health workers with that, because I feel at the same level as health workers – we are not different [others laugh].

I: any other additions on this? [No response] okay, and do you think house improvement committee members will continue with their role after the Majete project phases out?

P6: yeah, committee members will continue their role even if the project closes out. Why? Because the committee was appointed by villagers – it was not appointed by an animator or the project or committee members themselves, no. If the committee does not function properly, there are supervisors responsible for its operations. There’s a village chief supervising it. There’s an animator supervising it. Committee members are also committee supervisors themselves. So, if the committee is not functioning, the different supervisors can dissolve the committee and replace the members with new members. So the role of the committee will continue.

P3: the committee will continue performing its role. Why? Um, we have the village chief, who’s responsible for supervision of how the committee is operating. So, should any members slack in their performance just because the project phased out, it will be the responsibility of the chief to appoint new members. In that regard, the committee will continue its role, it can’t stop on the basis that the project phased out, because there are new members taking over.

<Files\\FA-C community participants FGD 2> - § 4 references coded [6.11% Coverage]

Reference 1 - 2.15% Coverage

Alright. So, I would like to know from you as people coming from different villages which are doing House Improvement work. How satisfied are you with the way houses in your villages are improved? ***(Question repeated for clarity)***. Let us start with P1.

**P1** We are satisfied with the way our houses are improved because wind does not enter the house. This is because when wind enters the house, it blows off the roof. So, since we improved the houses, wind does not enter the house any longer.

**Facilitator** Thank you. That’s P1. P8, do you have any additions on this? ***(Question repeated)***.

**P8** We are satisfied. When we improve the house, we get inside and look for any remaining spaces. If we find any, we close it. When we don’t find any, we are satisfied that the house has been improved properly. The other advantage is that wind no longer enters the house.

**Facilitator** Alright.

**P7** We do not hear any noises of rats or snakes when we get into the house when it is properly improved.

Reference 2 - 1.66% Coverage

us start with P6.

**P6** The change is in the way we are falling sick. People were frequently falling sick before this project. Malaria prevalence reduced with House Improvement.

**Facilitator** Alright. P1, I saw your hand was up.

**P1** People are infrequently falling sick with the coming of this project. People are doing development work. Besides, when we greet our neighbours, they say they are fine. However, previously the neighbours would say they are going to the hospital with the child, perhaps two children or a child and parent. So, we can now see the difference.

**Facilitator** Alright. That’s P1’s opinion. Let us hear from others. ***(Question repeated).*** P8?

**P8** The change is that we are not frequently falling sick and we are having physically fit bodies.

Reference 3 - 1.16% Coverage

I can tell them that the house they are staying in should be improved. It should not have spaces through the walls to prevent malaria. They should also be sleeping under mosquito nets.

**Facilitator** Alright. That is P2’s opinion.

**P7** When they come in our village, we need to explain to them the principles in our village. For example, on housing we can show them the exemplary houses which they need to copy when they are constructing theirs. We can tell them that we will not allow them to construct a house which is not improved

Reference 4 - 1.14% Coverage

Alright. I can explain to them the materials which are needed during House Improvement, for example in the eaves and the windows. Additionally, I can tell them that we they improve their house, they need to be sleeping under mosquito nets. I can also tell them that the advantage is you infrequently suffer from malaria with perhaps one person at a time. However, if you sleep in unimproved house, you suffer from malaria one person after the other. So, I can tell them the work need; to get bricks and hoe. The things we have

<Files\\FA-C HI Committee members FGD> - § 5 references coded [12.01% Coverage]

Reference 1 - 1.23% Coverage

How do you think the house improving work can be sustained?

**P7:** The way we have been working can be sustained because people’s lives are happy because the diseases are been reduced. There is development because malaria is reduced and this can be sustained.

**P3:** This can be sustained especially we the committee members and the village headmen work together. If the village headmen don’t take part, then others will not accept this project

Reference 2 - 2.08% Coverage

okay, is there something to add on this? Another follow up question is that how satisfied are you with the way houses in your communities are improved?

**P7:** we are satisfied because when we have finished the work, we sit down and reflect whether we have good job or not. If we haven’t done the good job, we get in the house and close the doors to see if there is light, then you redo the work if there is no light you are satisfied

**P8:** we are satisfied with the work because when we have finished the work, we inspect the house to see where there is light, if there is light somewhere, we close that gap

**P1:** when have finished closing the gaps, we close the door and when there is light, we are not satisfied unless there is darkness in the room

Reference 3 - 5.20% Coverage

Thank you very much. Less go to the last part. As committee, what do you think has improved on the issue of malaria since the house improving work started?

**P6:** in our community, only few people have gone to the hospital unlike before. This means house improving is going on well

**P4:** The change is that when the animator has conducted the meeting, there was a register to track every malaria patient an now, only one or there can be none

**P7:** the number of malaria patients is reduced because at the beginning, when people go to the hospital, most of them were malaria case but when this project started, only few people are diagnosed with malaria

**Facilitator:** what are your comments or ideas that you would like to tell people in your community for them to take part in the house improving work?

**P3:** the comment is just to encourage them that although we do not visit them, they should proceed doing house improvement so that they may be using their money on other issues and not malaria

**P8:** the comment is that when people have built the house, they are asking is the gauze wire, so this should not stop

**P7:** it’s better everyone to be the advisor on his own. If you were trained, you should become the advisor

Facilitator: okay, if you happen to be chosen to go to another community that is just starting this work of house improving, what can you tell such communities?

P7: I can tell my fellow committee members the advantages and the disadvantages because if I just tell them only advantages, they will be disappointed. But I should tell them that you will be facing these challenges and when you face them, be patient. If I have to talk at the community meeting, I can tell people the advantages and the disadvantages of this to the committee because some committee members are not patient, they can get someone who is not willing, they should be patient

Reference 4 - 1.57% Coverage

okay, is there anything to add? Okay you as committee members, do you think the house improving work will continue when the Majete project phases out?

**P6:** The work will continue because they have seen the advantage and the advantage is that few mosquito is getting in the house because of the gauze wires

**P4:** this will continue. Village headman, animator and the committee members will sit down and discuss on the continuation

**P1:** this work can continue, we can agree with the village head and the fellow committee members so that our village should be exemplary

Reference 5 - 1.93% Coverage

You as committee members, will you proceed working when Majete project will phase out?

P7: in our community it will continue because everyone will be aware of this and there is nothing that can worry the committee member because we are the owner of the community and Majete are not the owner, we should therefore protect our lives

P2: we will work because we have been trained

**Facilitator:** which procedures will you follow?

**P2:** we will sit down and reflect on which organization can help us because some materials are expensive

**P8:** as my colleagues have said, Majete people are visitors here, we are the owner. Majete came and trained us and if we don’t proceed, we have killed the community

<Files\\GVH KAMOGA> - § 1 reference coded [2.38% Coverage]

Reference 1 - 2.38% Coverage

how satisfied are you?

Respondent: I am satisfied. When they are closing, I go there and see if they have covered well or not. So I see that this person has covered well if he has not covered well I tell him that you did not cover well. Do this this so that things should be good

<Files\\GVH MAKANDE(1)> - § 1 reference coded [1.14% Coverage]

Reference 1 - 1.14% Coverage

Alright. Are you satisfied with the house improvement intervention is been done in your community.

Respondent: Yes

<Files\\HSA Liwonde- Kapichira Health Center KII> - § 1 reference coded [2.46% Coverage]

Reference 1 - 2.46% Coverage

Alright. Lastly, how satisfied are you with the way houses are improved in your village?

**HSA (*Laughs*)**. Currently, I am satisfied that the houses being improved currently are being done following the recommended methods because I, and the committee, am present when some people are improving their houses to help them with the work.

<Files\\KII MDZACHI(1)> - § 1 reference coded [1.40% Coverage]

Reference 1 - 1.40% Coverage

: how satisfied are with the way houses are been improved in your village?

Respondent: I do get satisfied because the house which is covered is a good one, not bad

Interviewer: mmm

Respondent: it is not bad

Interviewer: mmm

Respondent: because mosquitoes cannot enter
