# Supplementary material for: Assessing the implementation fidelity, feasibility, and sustainability of community-based house improvement for malaria control in southern Malawi: a mixed-methods study
Source: BMC Public Health. 2024 Apr 2;24:951. doi: 10.1186/s12889-024-18401-4 (PMC10988826; doi:10.1186/s12889-024-18401-4)
Supplement: Supplementary file 9 — Supplementary Material 9 [file 12889_2024_18401_MOESM9_ESM.docx]

**Assessing the implementation fidelity, feasibility, and sustainability of community-based house improvement for malaria control in southern Malawi: a mixed-methods study**

**Supplementary File 9: House characteristics in HI and non-HI houses at the Focal Area Level**

**Table S9: Table showing house characteristics in HI and non-HI houses in all focal areas**

| **House Characteristics** | **Focal Area (n)** | | | | | | **Total** | |
| --- | --- | --- | --- | --- | --- | --- | --- | --- |
|  | **Focal area A** | | **Focal area B** | | **Focal area C** | |  | |
|  | **HI** | **Non-HI** | **HI** | **Non-HI** | **HI** | **Non-HI** | **HI** | **Non-HI** |
|  |  |  |  |  |  |  |  |  |
| Houses visited | 499 | 372 | 439 | 469 | 190 | 748 | 1128 | 1589 |
|  |  |  |  |  |  |  |  |  |
| **Eaves** |  |  |  |  |  |  |  |  |
| Houses that attempted to have eaves closed | 452 (90.6%) | 286 (76.9%) | 251 (57.2%) | 138 (29.4%) | 128 (67.4%) | 369 (49.3%) | 831 (73.7%) | 793 (49.9%) |
| Houses with eaves completely closed on all 4 sides | 398 (79.8%) | 70 (18.8%) | 137 (31.2%) | 69 (14.7%) | 90 (47.4%) | 291 (38.9%) | 625 (55.4%) | 430 (27.1%) |
|  |  |  |  |  |  |  |  |  |
| **Windows** |  |  |  |  |  |  |  |  |
| Houses with windows | 456 (91.4%) | 328 (88.2%) | 391 (89.1%) | 350 (74.6%) | 125 (65.8%) | 537 (71.8%) | 972 (86.2%) | 1215 (76.5%) |
| Houses with no windows | 43 (8.6%) | 44 (11.8%) | 48 (10.9%) | 119 (25.4%) | 65 (34.2%) | 211 (28.2%) | 156 (13.8%) | 374 (23.5%) |
| Houses with windows that can be closed* | 401 (87.9%) | 166 (50.6%) | 281 (71.9%) | 147 (42.0%) | 109 (87.2%) | 394 (73.4%) | 791 (81.4%) | 707 (58.2%) |
| Houses with screened windows with gauze wire* | 412 (90.4%) | 32 (9.8%) | 213 (54.5%) | 29 (8.3%) | 90 (72.0%) | 21 (3.9%) | 715 (73.6%) | 82 (6.7%) |
|  |  |  |  |  |  |  |  |  |
| **Door Material** |  |  |  |  |  |  |  |  |
| Wood | 423 (84.8%) | 296 (79.6%) | 434 (98.9%) | 443 (94.5%) | 177 (93.2%) | 656 (87.7%) | 1034 (91.7%) | 1395 (87.8%) |
| Reed | 70 (14.0%) | 72 (19.4%) | 3 (0.7%) | 19 (4.1%) | 12 (6.3%) | 90 (12.0%) | 85 (7.5%) | 181 (11.4%) |
| No Covering | 1 (0.2%) | 3 (0.8%) | 1 (0.2%) | 5 (1.1%) | 1 (0.5%) | 4 (0.5%) | 3 (0.3%) | 12 (0.8%) |
| Other material | 5 (1.0%) | 1 (0.3%) | 1 (0.2%) | 2 (0.4%) | 0 (0) | 1 (0.1%) | 6 (0.5%) | 4 (0.3%) |
| Houses with doors containing spaces |  |  |  |  |  |  |  |  |
|  |  |  |  |  |  |  |  |  |
| **Roof Material** |  |  |  |  |  |  |  |  |
| Natural material | 379 (76.0%) | 285 (76.6%) | 250 (56.9%) | 346 (73.8%) | 98 (51.6%) | 398 (53.2%) | 727 (64.5%) | 1029 (64.8%) |
| Iron Sheets | 120 (24.0%) | 87 (23.4%) | 187 (42.6%) | 119 (25.4%) | 92 (48.4%) | 350 (46.8%) | 399 (35.4%) | 556 (35.0%) |
| Iron and Tiles | 0 (0) | 0 (0) | 2 (0.5%) | 4 (0.9%) | 0 (0) | 0 (0) | 2 (0.1%) | 4 (0.3%) |
| Cement | 0 (0) | 0 (0) | 0 (0) | 0 (0) | 0 (0) | 0 (0) | 0 (0) | 0 (0) |
|  |  |  |  |  |  |  |  |  |
| **Wall Material** |  |  |  |  |  |  |  |  |
| Mud/dung | 152 (30.5%) | 106 (28.5%) | 6 (1.4%) | 8 (1.7%) | 0 (0) | 2 (0.3%) | 158 (14.0%) | 116 (7.3%) |
| Sun-dried brick | 20 (4.0%) | 25 (6.7%) | 82 (18.7%) | 143 (30.5%) | 58 (30.5%) | 216 (28.9%) | 160 (14.2%) | 384 (24.2%) |
| Fire-baked brick | 324 (64.9%) | 241 (64.8%) | 349 (79.5%) | 316 (67.4%) | 132 (69.5%) | 528 (70.6%) | 805 (71.4%) | 1085 (68.3%) |
| Iron Sheets | 2 (0.4%) | 0 (0) | 0 (0) | 0 (0) | 0 (0) | 1 (0.1%) | 2 (0.2%) | 1 (0.1%) |
| Wood | 0 (0) | 0 (0) | 0 (0) | 0 (0) | 0 (0) | 1 (0.1%) | 0 (0) | 1 (0.1%) |
| Other material | 1 (0.2%) | 0 (0) | 2 (0.5%) | 2 (0.4%) | 0 (0) | 0 (0) | 7 (0.6%) | 1 (0.1%) |
|  |  |  |  |  |  |  |  |  |
| **Floor Material** |  |  |  |  |  |  |  |  |
| Dirt/mud/sand/dung | 477 (95.6%) | 355 (95.4%) | 347 (79.0%) | 431 (90.2%) | 164 (86.3%) | 639 (85.4%) | 988 (87.6%) | 1425 (89.6%) |
| Wood/ Plank | 0 (0) | 0 (0) | 2 (0.5%) | 1 (0.2%) | 0 (0) | 5 (0.7%) | 2 (0.2%) | 6 (0.4%) |
| Cement | 22 (4.4%) | 17 (4.6%) | 89 (20.3%) | 37 (7.4%) | 26 (13.7%) | 103 (13.8%) | 137 (12.1%) | 157 (9.9%) |
| Tiles for main floor | 0 (0) | (0) | 1 (0.2%) | 0 (0) | 0 (0) | 1 (0.1%) | 1 (0.1%) | 1 (0.1%) |
